# Differences between experimental and placebo arms in manual therapy trials: a methodological review

**DOI:** 10.1186/s12874-022-01704-8

**Published:** 2022-08-08

**Authors:** D.’Alessandro Giandomenico, Ruffini Nuria, Aquino Alessandro, Galli Matteo, Innocenti Mattia, Tramontano Marco, Cerritelli Francesco

**Affiliations:** 1 Clinical-Based Human Research Department, Foundation C.O.ME. Collaboration, 65121 Pescara, Italy; 2 Centre Pour L’Etude, La Recherche Et La Diffusion Ostéopathiques “C.E.R.D.O”, 00199 Rome, Italy; 3 Foundation C.O.ME. Collaboration, National Centre Germany, 10825 Berlin, Germany; 4 Department of Health Sciences, University of Milan, 20142 Milan, Italy; 5 Research Department, SOMA, Istituto Osteopatia Milano, Milan, Italy; 6 Fondazione Santa Lucia IRCCS, 00179 Rome, Italy

**Keywords:** Manual therapies, Placebo, similarity, Sham therapy, Manual placebo, Systematic review

## Abstract

**Background:**

To measure the specific effectiveness of a given treatment in a randomised controlled trial, the intervention and control groups have to be similar in all factors not distinctive to the experimental treatment. The similarity of these non-specific factors can be defined as an equality assumption. The purpose of this review was to evaluate the equality assumptions in manual therapy trials.

**Methods:**

Relevant studies were identified through the following databases: EMBASE, MEDLINE, SCOPUS, WEB OF SCIENCE, Scholar Google, clinicaltrial.gov, the Cochrane Library, chiloras/MANTIS, PubMed Europe, Allied and Complementary Medicine (AMED), Physiotherapy Evidence Database (PEDro) and Sciencedirect.

Studies investigating the effect of any manual intervention compared to at least one type of manual control were included. Data extraction and qualitative assessment were carried out independently by four reviewers, and the summary of results was reported following the PRISMA statement.

**Result:**

Out of 108,903 retrieved studies, 311, enrolling a total of 17,308 patients, were included and divided into eight manual therapy trials categories. Equality assumption elements were grouped in three macro areas: patient-related, context-related and practitioner-related items. Results showed good quality in the reporting of context-related equality assumption items, potentially because largely included in pre-existent guidelines. There was a general lack of attention to the patient- and practitioner-related equality assumption items.

**Conclusion:**

Our results showed that the similarity between experimental and sham interventions is limited, affecting, therefore, the strength of the evidence. Based on the results, methodological aspects for planning future trials were discussed and recommendations to control for equality assumption were provided.

**Supplementary Information:**

The online version contains supplementary material available at 10.1186/s12874-022-01704-8.

## Background

‘Manual Therapy’ (MT) is an umbrella term used and variously defined by different professional groups [1–4]. The definitions differ mainly for type of operator, presence of a hand-guided instrument, co-presence of exercises, target tissue of the treatment [5], clinical goals, and the active/passive role of the patient in the process of care. Consequently, it is possible to consider a more extensive sense of MT including manipulation, mobilisation, massage [6], but also acupressure, nerve manipulation [7] and gentle skin touch [8, 9] applied with a therapeutic intent [7] on the patient’s body [10]. MT is one of the oldest known forms of medicine and has been practised worldwide since ancient times [6, 11–13], and the interest in MT has grown in the last years, with patients expressing a growing satisfaction for the offered service [14] In analogy to other fields of clinical research, the randomised control trial (RCT) is also regarded as the gold standard [15] in manual therapy research due to its robust methodology and ability to conduct systematic reviews and meta-analyses. One of the pillars of an RCT is the use of a control group or placebo intervention, known in manual therapy RCT (mtRCT) as ‘sham therapy [16]. The use of a placebo arm is crucial to disentangle the specific effect of the experimental treatment from the non-specific or not distinctive effects of a given treatment [17–19]. There are currently no guidelines addressing how to conduct appropriate sham therapy to ensure the robustness of mtRCT’s methodology and results.

It is worth noting that the placebo effect is considered more relevant in non-pharmacological treatments [20, 21] including complementary alternative medicines (CAMs) [20, 22]. It depends on several conditions, including the significant role of interpersonal touch [9], the multiplicity of treatment sessions [23], and the optimisation of the patient-physician relationship [24–26,27]. In light of the science of placebo [28] has been proposed that one fundamental pillar of an RCT is the guaranteed similarity between non-specific factors in both intervention and sham arms. The entire paradigm has been recently described by Annoni and Boniolo [29] and can be defined as follows: *“the specific efficacy (SE) of a treatment (x) is equal to the overall improvement measured in the experimental group (Ix) minus the improvement measured in the control group (Ic)”*, thus SEx = Ix – Ic [29]. One of the elements ensuring strength to the equation is the robustness of the “equality assumption” (EA), that is the overlap of non-specific aspects between groups, e.g. the same patient-operator relationship in the experimental and placebo groups. Although some authors in MT research claim a similarity between the experimental and sham arms of the trial [15, 27, 30–34], there is not an organic perspective that takes into account the science of placebo. A recent systematic review demonstrated an incongruity among sham and experimental treatment procedures in osteopathic trials, which hinders the evaluation of the actual magnitude of the specific effect of a therapy [16]. This might lead to skewed results with potentially detrimental consequences for healthcare decision making [35].

The purpose of this review is to systematically report the similarity of non-specific factors between experimental and placebo arms in mtRCTs in other research fields, outlying EA in 3 macro-areas—patients, operators and context. Moreover, differences between manual therapies and/or manual approaches were highlighted and evaluated.

## Methods

The present review followed the PRISMA (Preferred Reporting Items for Systematic Reviews and Meta-Analyses) statement [36], and included multi-centre, single-centre, quasi-randomised and randomised clinical controlled trials, interrupted time series, and controlled clinical trials. All included studies investigated the effect of any manual intervention compared to at least one type of manual control, sham and/or placebo intervention with direct contact between practitioner and subjects.

### Inclusion/exclusion criteria

No limit of population, study outcome, and language restriction [37], was applied. Non-peer reviewed papers, conference proceedings, editorials, letters, abstracts, case reports, and case series, were excluded. Studies investigating the effect of osteopathic manipulative treatment were also excluded as previously explored by Cerritelli and colleagues [16]. Research utilising either control without direct touch, i.e., interposing any material between the operator's hand and the patient, or non-manual control interventions only were excluded.

### Search methods, selection and evaluation of studies

Relevant studies were identified through a comprehensive computerised bibliographic search on the following databases: EMBASE, MEDLINE, SCOPUS, WEB OF SCIENCE, Scholar Google, clinicaltrial.gov, the Cochrane Library, chiloras/MANTIS, PubMed Europe, Allied and Complementary Medicine (AMED), Physiotherapy Evidence Database (PEDro) and Sciencedirect. The search strategy used is detailed in Supplementary Information S1, available online. All searches were carried out from inception to 2021. Duplicate records were identified and removed using the software EndNOTE.

GDA and NR developed and ran the search from March to April 2019 with an update in February 2022, and included studies until 2021. The first screening of titles and abstracts gathered through bibliographic searches was independently carried out by two reviewers (GDA and NR), based on the pertinence and relevance of each study to inclusion and exclusion criteria. Discrepancies were resolved by consensus with FC as an arbiter. Full texts were subsequently assessed for inclusion. Reviewers were able to translate to English from French, Spanish, German, and Italian. For other languages, a translation to English was required from the authors. In case of unsuccessful contact, the study was excluded.

Data extraction and the qualitative assessment of included studies were carried out independently by four reviewers (GDA, MT, AA, NR). Extracted and summarised data included: type of intervention, type of control, sample size, study outcomes, and other potentially relevant characteristics. Authors were contacted twice, separated by three weeks [34] when provided information was insufficient, and, where possible, the reasons for their omission were reported (details in Supplementary Table S2, available online). All data were archived on a shared fully encrypted server, accessible only to the four reviewers. Disagreements were discussed and resolved by consensus.

### Data synthesis

Data were reported as means, point estimates, percentages and ranges. X^2^ test was used to compare groups. The distribution of Chi-square residuals was also used to determine which categories leads the eventual significant difference.

### MTs classification in the present review

As pointed out by Farrel and Jessen, MT is not specific to any profession [38]. Indeed, the same approach or technique could be used by different MTs [39]. Therefore, the included papers were grouped according to 3 criteria:

- (1) Single category: when an MT uses techniques that are unique to a discipline, it was considered as a single category. It is the case of ‘acupressure’, ‘reiki’, ‘reflexology’ and ‘therapeutic touch’.
- (2) Grouped by therapies: when different MTs showed common features, a broader category has been considered, as in the case of ‘massage’.
- (3) Grouped by techniques: when authors used manual techniques that are not distinctive for a specific MT (e.g., thrust or high-velocity low-amplitude techniques could be used in physiotherapy, chiropractic and orthopaedics), the following categories were used, based on Coulter et al. [40]: manipulation’ (or ‘thrust’) and ‘mobilisation’ (or ‘non-thrust’). The latter included neurodynamic techniques, Muscle Energy Techniques, and tender/trigger point. Studies encompassing both thrust and non-thrust techniques were grouped into the ‘mixed-method’ category.

### Studies with more than one sham group

When a study had two manual sham groups used to control for two interventions, it was considered as two different studies.

### EA score

To evaluate the EAs related to the three macro-areas, the authors assigned one point per item investigated.

The patient-related EAs were described based on the following characteristics: patients’ expectations, deblinding questionnaire or interview, credibility questionnaire or interview, patients’ previous experiences with the given therapy, psychological traits and reimbursement to patients (score range 0 to 6).

The context-related EAs was based on the following characteristics: frequency of sessions, treatment period, description of the pre-treatment phase, detailed description of the sham therapy protocol, overlap of body areas treated between intervention and sham therapy, duration of experimental and sham intervention, description of the post-treatment phase, setting for interventions, time-points assessment, and side effect (score range 0 to 10).

Regarding the practitioners-related EAs, the following characteristics were considered: the number of practitioners, type of practitioners, years of practitioners’ experience, pre-trial training for practitioners, mean age of practitioners, and gender of the practitioner (score range 0 to 6). The determination of EA was performed by two reviewers (GDA, NR), and the discrepancies were resolved by consensus with a third reviewer (FC) as an arbiter.

## Results

**Fig. 1:**
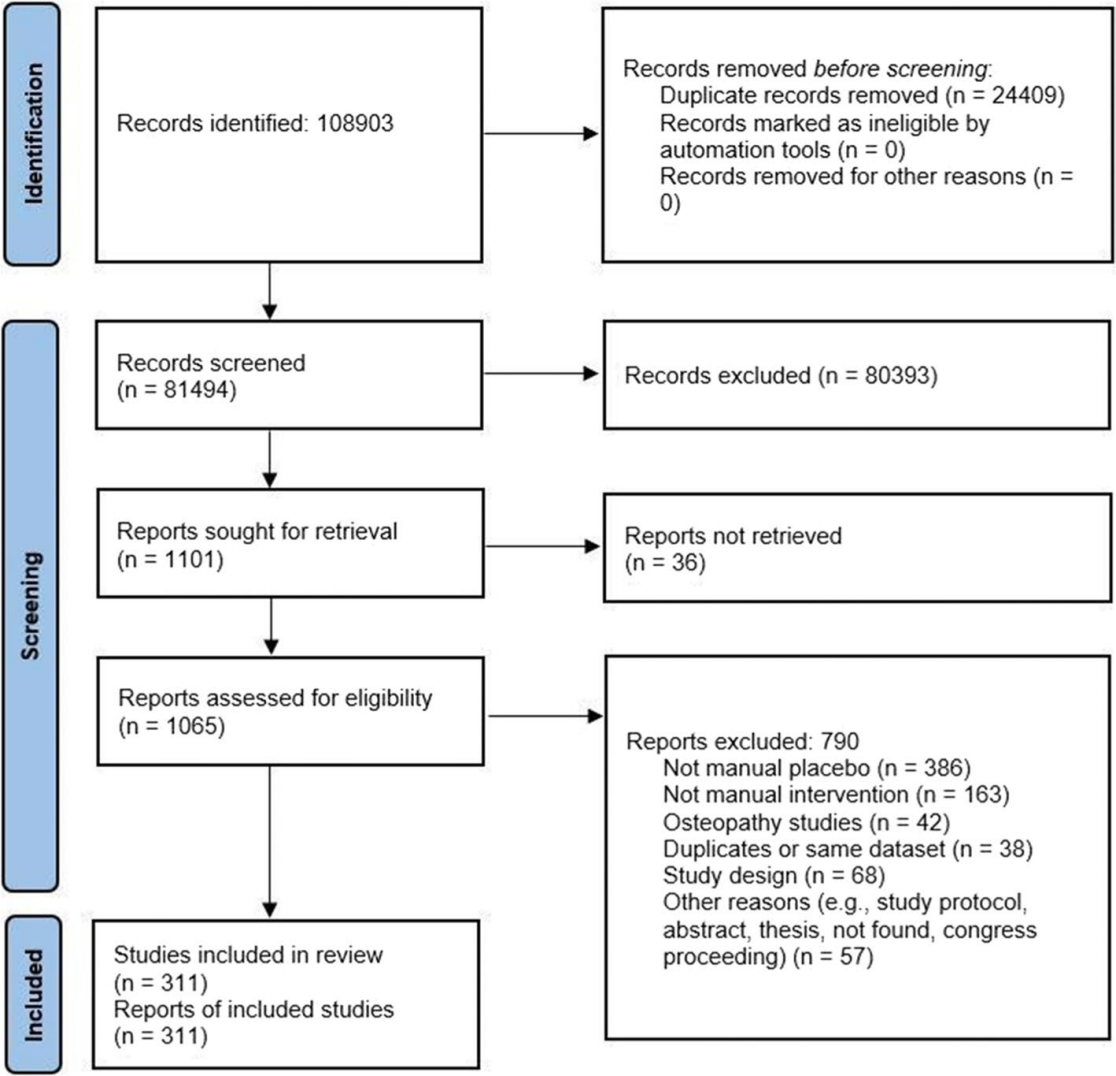
Flow-chart of the study

A total of 108,903 records were identified through database searching and other sources. After the removal of duplicates, 81,494 titles and abstracts were screened. 1101 full-text articles were consequently assessed for eligibility. 790 articles were excluded for not respecting the inclusion criteria, or because full-texts were unavailable. Data and publications from the same study were considered as duplicates and therefore excluded from the systematic review. The final sample included 311 studies, enrolling a total of 17,308 patients, of which 6053 were males (35.0%) (Fig. 1). Thirteen studies did not report the gender of their participants.

The first analysis showed that four studies included two sham groups. Three studies [41–43] were considered as double because they used two different sham groups to control for two different intervention groups: in Geisser et al. 2015 [41] the two interventions (manual therapy + adjuvant physical exercises; manual therapy + non-specific exercises) were compared to two sham groups (sham manual therapy + adjuvant physical exercises; sham manual therapy + non-specific exercises). Haik and colleagues [42] investigated the effect of thoracic spine thrust manipulation on symptomatic and asymptomatic subjects, compared to sham thoracic spine thrust manipulation on respectively symptomatic and asymptomatic subjects. In Nansel and colleagues [43] two different techniques (upper cervical and lower cervical adjustment) were respectively compared to two different sham therapies (sham upper cervical and sham lower cervical manipulation).

In Bialosky and colleagues [44] basic and enhanced sham therapy were used as a control for only one intervention on the same kind of population, it was considered as double because of the number of sham arms.

Based on the number of sham therapy arms, the total number (N) of the studies included in the review was, therefore, 315. The latter was used as N for the analysis of the EAs, whereas 311 studies were considered for describing the general characteristics of the studies.

All the results are reported in the tables, and only statistically significant results have been highlighted in the main text.

The included sample comprehended a number of different therapeutic approaches, descriptively: 77 studies investigated the effect of acupressure (24.8%); 8 were relative to massage (2.6%); 2 to reiki (0.6%), 20 to reflexology (6.4%), 3 considered therapeutic touch (0.96%), 108 mobilisation (34.7%), 89 manipulation (28.6%) and 4 used a mixed-method approach (1.3%).

206 studies (66.2%) investigated symptomatic subjects, 104 studies (33.4%) included asymptomatic participants and 1 study (0.3%) included both symptomatic and non-symptomatic patients.

The global mean age for the participants in the studies was 37.4 years (Table 1).

**Table 1:** General characteristics of the population and methodological characteristics of the studies included in the review

	**Acu**	**Mas**	**Rei**	**Ref**	**TT**	**Mob**	**Man**	**Mix**	**Total**
*N studies*	77	8	2	20	3	108	89	4	311
*sample size*	6155	506	289	1192	105	4537	4221	303	17,308
*mean age (years)*	35.6	30.9	61.5	41.3	50.5	32.5	29.8	17	37.4
*Male* *(%)*	1609 (26.1)	424 (83.8)	8 (2.8)	314 (26.3)	29 (27.6)	1712 (37.7)	1772 (42.0)	185 (61.1)	6053 (35.0)
*- Parallel*	74	8	2	19	2	79	76	4	264
*- Crossover*	3	0	0	1	1	29	13	0	47
*PROM (yes)*	29	1	2	11	0	39	24	3	109
*PROM (no)*	35	4	0	7	1	40	47	1	135
*PROM (both)*	13	3	0	2	2	29	18	0	67
*Operator dependent measurements ( yes)*	10	1	0	9	0	26	21	1	68
*Operator dependent measurements (no)*	65	6	2	7	1	71	63	3	218
*Operator dependent measurements (both)*	2	1	0	4	2	11	5	0	25
*Blinding (double)*	15	2	1	7	1	43	15	2	86
*Blinding (single)*	35	3	0	6	2	36	29	1	112
*Blinding (not declared)*	27	3	1	7	0	29	45	1	113
*Source of enrolment (yes)*	56	7	2	15	3	77	65	3	228
*Source of enrolment (NR)*	21	1	0	5	0	31	24	1	83

*N* Number, *Acu* Acupressure *Mas* Massage, *Rei* Reiki, *TT* T herapeutic Touch, *Mob* Mobilisation, *Man* Manipulation, *Mix* Mixed-Method, *NR* Not reported

As per methodological design, 264 (84.9%) trials used a parallel design and 47 (15.1%) used a crossover-design. The Chi-squared analysis showed a significant difference among therapies, with acupressure and mobilisation choosing a parallel design more than the other therapies (X^2^ = 24.034.62, *p* = 0.001). Additional details regarding the intervention and control arms are summarised in Supplementary Table S3, available online.

Of the 311 included trials, 86 (27.7%) declared to use a double-blind design, 112 studies (36.0%) were defined as single-blinded, and 113 studies (36.3%) did not define the type of blinding. The Chi-square analysis showed that significantly more mobilisation studies reported a double blind design, and manipulation not reporting the kind of blinding (X^2^ = 26.19, *p *= 0.02).

109 (35.1%) studies utilised patient-reported outcomes (PROMs), 135 (43.4%) used exclusively outcomes measured using devices, 67 (21.5%) used both PROMs and instruments. 68 studies (21.9%) considered operator-dependent outcome measurements. In 218 studies (70.1%), the outcome was not operator dependent. In 25 studies (8.0%), both types of outcomes were assessed. The Chi-square analysis showed a significant difference among therapies (X^2^ = 37.578, *p* = 0.006), with therapeutic touch using mostly both types of outcome measurement, and reflexology choosing operator-dependent outcomes.

73.3% (*N* = 228) of studies described the source of enrolment, whereas 83 (26.7%) studies did not give any information (Table 1).

### Patients’ EA

A total of 26 studies (8.3%) investigated patients’ expectations about the treatment. The chi-squared analysis showed that massage and reiki investigated patients’ expectations significantly more than the other categories (X^2^ = 46.296, *p* < 0.0001). In all 26 studies, patients’ expectations between treatment and sham arms were homogeneous at the baseline.

The majority of studies (272/315, 86.4%) did not perform any deblinding procedures. The Chi-Squared analysis showed a prevalence of acupressure not investigating the deblinding and of manipulation performing a deblinding procedure (X^2^ = 18.022, *p* = 0.01). Among the 43 studies that fulfilled the deblinding process, 40 showed homogeneity between study arms, whereas the remaining 3 trials demonstrated heterogeneity (X^2^ = 31.837, *p* < 0.001).

The credibility of the provided treatment, according to patients, was not investigated in the majority of studies (291/315, 93.6%). In 23 studies, the credibility of the provided treatment according to patients, between treatment and sham arms was homogeneous, except for Bialosky et al. 2014 [45].

Patients’ previous experiences with the investigated intervention were not reported by 76.8% of studies (242/315). Among the remaining 73, 68 studies (93.2%) included only patients who were naive to the investigated intervention, being therefore homogeneous at baseline. In the remaining 5 studies, participants had previous experiences with the given therapy. 4 of them were manipulation studies, hence determining a statistically significant difference among treatments (X^2^ = 11.012 *p* = 0.05). Furthermore, two out of five papers did not report whether the groups were homogeneous about this characteristic.

Regarding psychological features, 282/315 studies (89.5%) did not investigate the psychological features of patients. The Chi-squared analysis showed that massage, reflexology and therapeutic touch considered the psychological features of subjects significantly more than other categories (X^2^ = 31.916, *p* < 0.0001). In all remaining 33 studies, patients' psychological features between treatment and sham arms were homogeneous at the baseline.

**Fig. 2:**
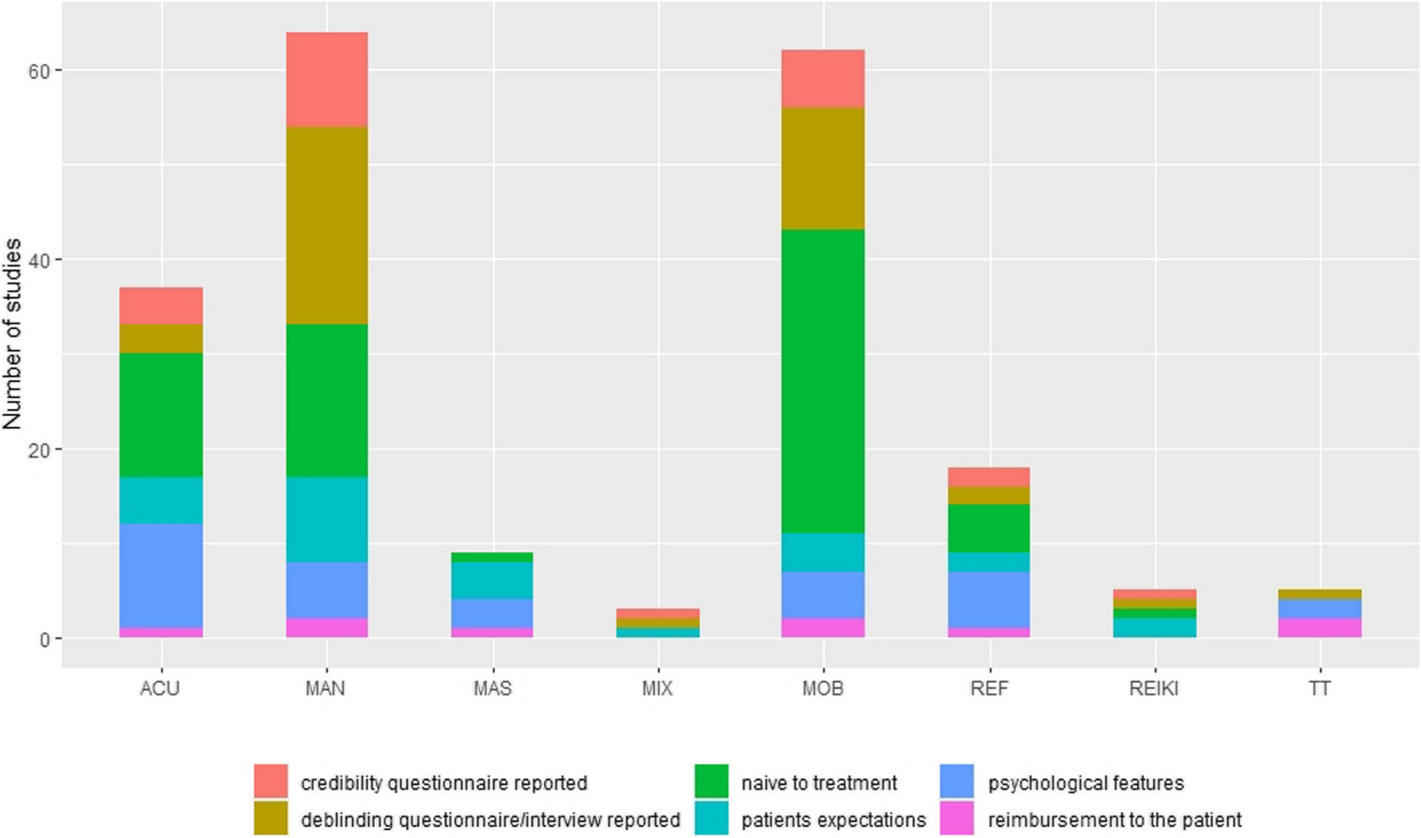
Equality assumption for patient-related characteristics of the included studies

Regarding the reimbursement to patients, the quasi-totality of trials (306/315, 97.1%) did not declare whether reimbursement was issued. Therapeutic touch reported this information more than the other therapies (X^2^ = 48.136, *p* < 0.0001). The issued reimbursement was homogeneous among groups in all the studies that reported the information but Zeidabadinejad et al., where only the sham group was offered the real intervention after the trial’s ending (Fig. 2).

The patient-related EAs score was 0/6 in 195 studies (61.9%), 1/6 in 83 studies (26.3%), 2/6 in 23 studies (7.3%), 3/6 in 9 studies (2.9%), 4/6 in 3 studies (0.9%), 5/6 in 2 studies (0.6%) (Table 2).

**Table 2:** Patient-related equality assumption score

	**Acu** (*n* = 77)	**Mas** (*n* = 8)	**Rei** (*n* = 2)	**Ref** (*n* = 20)	**TT** (*n* = 3)	**Mob** (*n* = 109)	**Man** (*n* = 92)	**Mix** (*n* = 4)	**Total** (*n* = 315)
*0/6*	54 (70.1%)	5 (62.5%)	0 (0%)	10 (50.0%)	0 (0%)	65 (56.6%)	58 (63.0%)	3 (75.0%)	195 (61.9%)
*1/6*	19 (24.7%)	2 (25.0%)	1 (50.0%)	7 (35.0%)	3 (100.0%)	34 (31.2%)	17 (18.5%)	0 (0%)	83 (26.4%)
*2/6*	1 (1.3%)	1 (12.5%)	1 (50.0%)	2 (10.0%)	0 (0%)	7 (6.4%)	11 (12.0%)	0 (0%)	23 (7.3%)
*3/6*	0 (0%)	0 (0%)	0 (0%)	1 (5.0%)	0 (0%)	2 (1.8%)	6 (6.5%)	0 (0%)	9 (2.9%)
*4/6*	1 (1.3%)	0 (0%)	0 (0%)	0 (0%)	0 (0%)	1 (0.9%)	0 (0%)	1 (25.0%)	3 (0.9%)
*5/6*	2 (2.6%)	0 (0%)	0 (0%)	0 (0%)	0 (0%)	0 (0%)	0 (0%)	0 (0%)	2 (0.6%)

Numbers in table are referred to the actual number of studies reporting the respective item

*N* Number, *Acu* Acupressure, *Mas* Massage, *Rei* Reiki, *TT* Therapeutic Touch, *Mob* Mobilisation, *Man* Manipulation, *Mix* Mixed-Method

No studies scored 6/6

### Context related EA

302/315studies (95.9%) reported the same frequency of session for different intervention groups. The quasi-totality of studies (300/315, 95.2%) reported a similar treatment period among groups.

The pre-treatment phase, intended as the protocolled process preceding the treatment (e.g., baseline measurements, preparation of the patient), was described as the same for both experimental and sham interventions in 255/315 studies (81.0%), 1 study (0.3%) used different pre-treatment phases, and 59 studies (18.7%) did not report sufficient information to evaluate this specific EA, especially in reflexology (X^2^ = 26.19, *p* = 0.02).

308/315 studies (97.8%) reported adequate details to establish the similarity of the applied technique between experimental and sham arms. There is, however, a significant difference among therapies, with therapeutic touch describing the sham technique less than the other categories (X^2^ = 24.142, *p* < 0.001).

Concerning the areas of intervention, in 280/315 studies (88.9%) intervention and sham techniques targeted the same bodily regions and/or tissue; in 25/315 studies (7.9%) experimental and sham therapy were applied to different areas, and 10/315 studies (3.2%) did not report sufficient or clear information. The Chi-squared analysis showed that reiki and therapeutic touch gave less information than other MTs (X^2^ = 30.445, *p*0.007).

In 212/315studies (67.39%) experimental and sham intervention had the same duration. 98/315 studies (31.1%) reported insufficient or unclear data, especially in manipulation studies (X^2^ = 108.43, *p* < 0.0001).

The post-treatment phase, intended as the process following the intervention (e.g., post-treatment measurements), was described as the same for both experimental and sham interventions in 196/315 studies (62.2%), 119/315 studies (37.8%) did not report sufficient information, mostly in reflexology (X^2^ = 17.61, *p* = 0.01).

The setting for intervention was reported as the same among groups in 214/315 studies (67.9%).

The number of time points assessments was reported as the same among intervention and sham groups in all included trials.

**Fig. 3:**
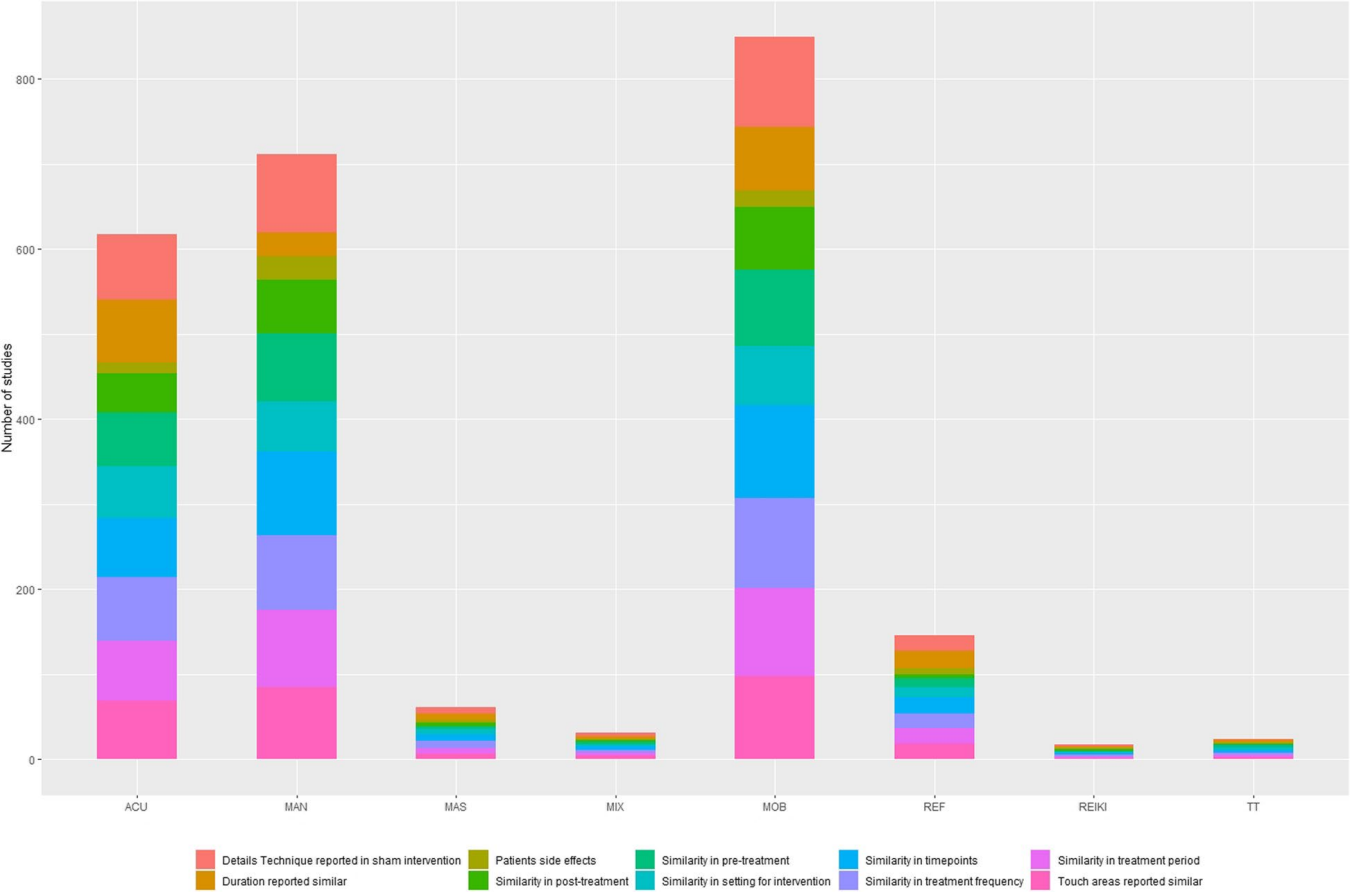
Equality assumption for context-related characteristics of the included studies. *Acu* Acupressure, *Mas* Massage, *Rei* Reiki, *TT* Therapeutic Touch, *Mob* Mobilisation, *Man* Manipulation, *Mix* Mixed-Method

In a total of 315 included studies, 242(76.8%) did not collect or report data on side effects after either sham or experimental intervention (Fig. 3).

The context-related EAs score was 4/10 in 6 studies (1.9%), 5/10 in 8 studies (2.5%), 6/10 in 28 studies (8.93%), 7/10 in 62 studies (19.7%), 8/10 in 109 studies (34.6%), 9/10 in 83 studies (26.3%), 10/10 in 19 studies (6.0%) (Table 3).

**Table 3:** Context-related equality assumption score

	**Acu** (*n* = 77)	**Mas** (*n* = 8)	**Rei** (*n* = 2)	**Ref** (*n* = 20)	**TT** (*n* = 3)	**Mob** (*n* = 109)	**Man** (*n* = 92)	**Mix** (*n* = 4)	**Total** (*n* = 315)
*4/10*	1 (1.3%)	0 (0%)	0 (0%)	1 (5.0%)	0 (0%)	1 (0.9%)	3 (3.3%)	0 (0%)	6 (1.9%)
*5/10*	2 (2.6%)	0 (0%)	0 (0%)	1 (5.0%)	0 (0%)	1 (0.9%)	3 (3.3%)	1 (25.0%)	8 (2.5%)
*6/10*	2 (2.6%)	1 (12.5%)	0 (0%)	2 (10.0%)	0 (0%)	14 (12.8%)	9 (9.8%)	0 (0%)	28 (8.9%)
*7/10*	16 (20.8%)	1 (12.5%)	0 (0%)	4 (20.0%)	1 (33.3%)	20 (18.3%)	20 (21.7%)	0 (0%)	62 (19.7%)
*8/10*	24 (31.2%)	4 (50.0%)	1 (50.0%)	6 (30.0%)	1 (33.3%)	40 (36.7%)	32 (34.8%)	1 (25.0%)	109 (34.6%)
*9/10*	25 (32.5%)	2 (25.0%)	1 (50.0%)	5 (25.0%)	0 (0%)	26 (22.0%)	22 (23.9%)	2 (50.0%)	83 (26.3%)
*10/10*	7 (9.1%)	0 (0%)	0 (0%)	1 (5.0%)	1 (33.3%)	7 (6.4%)	3 (3.3%)	0 (0%)	19 (6.0%)

No studies scored 0/10 to 3/10

*Acu* Acupressure, *Mas* Massage, *Rei* Reiki, *TT* Therapeutic Touch, *Mob* Mobilisation, *Man* Manipulation, *Mix* Mixed-Method

### Practitioner related EA

As expected, the majority of the studies (242/315, 76.8%), declared how many practitioners delivered the different interventions, although 23.2% (73/315) underreported the numbers of operators involved.

Overlapping results were shown for the type of practitioner, where 78.4% (247/315) declared to have enrolled the same type of practitioner for experimental and sham interventions. The Chi-squared showed a significance for reiki, in which both studies used different types of practitioners for intervention and control groups (X^2^ = 249.23, *p* < 0.0001).

Regarding the experience of practitioners 66.0% studies (208/315) reported unclear or no information; the 2 studies investigating the effect of reiki used practitioners with a different experience for intervention and control groups, thus determining a statistical significance imbalance (X^2^ = 290.975, *p* < 0.0001).

208/315 (66.0%) of the research included did not report whether or not practitioners were trained before the study, with a higher prevalence for mobilisation; whereas acupressure reported the practitioner training more than other catergories (X^2^ = 74.084, *p* < 0.0001).

The mean age of practitioners was not reported in 294/315 (93.3%) studies and the only 21 studies (6.7%) that reported the age of the person who intervened were the acupressure trials in which the patients performed a self-treatment, thus determining a statistical significance (X^2^ = 69.55, *p* < 0.001).

**Fig. 4:**
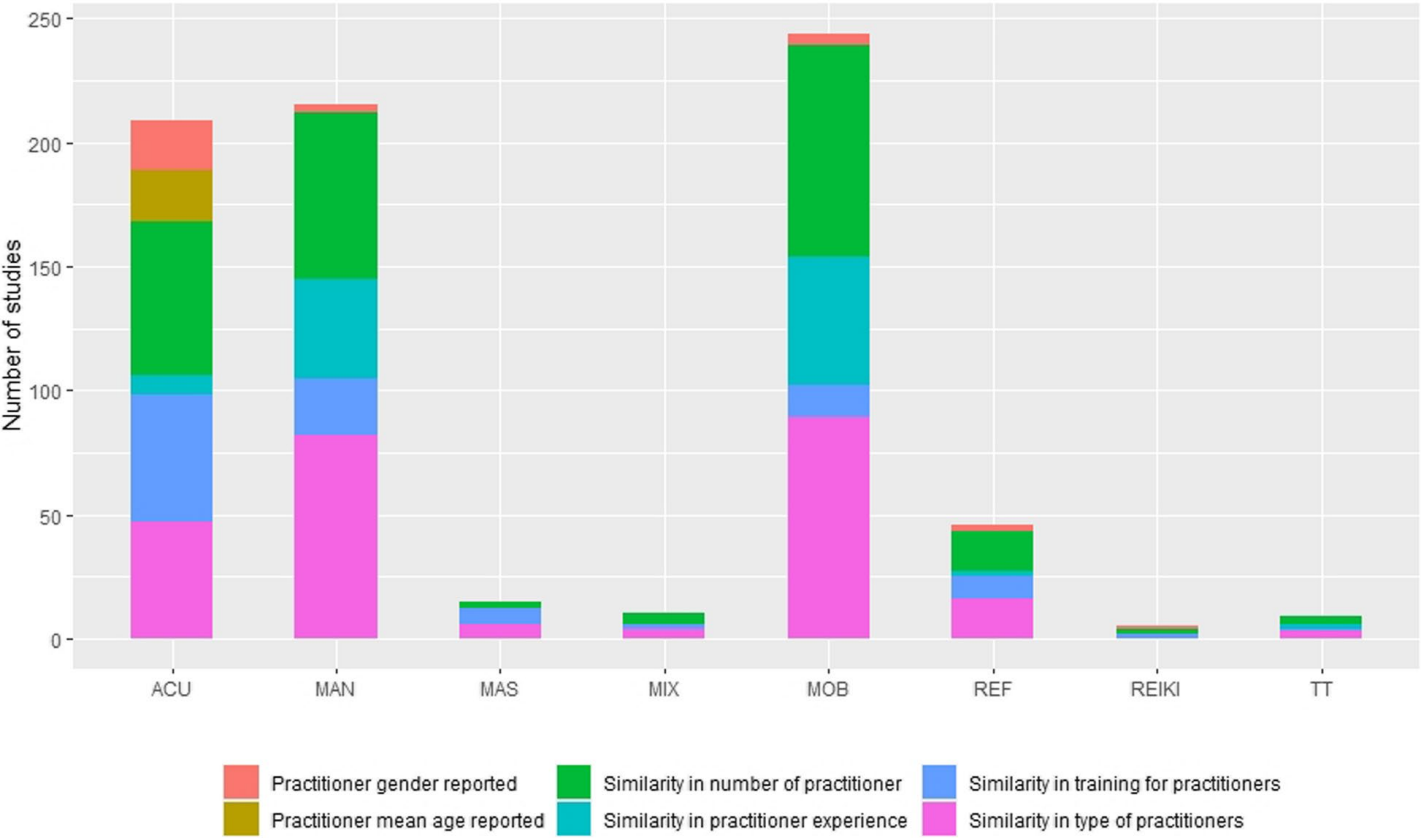
Equality assumption for practitioner-related characteristics of the included studies. *Acu* Acupressure, *Mas* Massage, *Rei* Reiki, *TT* Therapeutic Touch, *Mob* Mobilisation, *Man* Manipulation, *Mix* Mixed-Method

Where the practitioners’ gender is considered, 283/315 studies did not report it (89.8%). The remaining 32 trials reported the gender of the operator, with a prevalence of acupressure (X^2^ = 35.294 *p* < 0.001). The 20 acupressure studies that reported the practitioners’ gender performed a self-administered intervention (Fig. 4).

The practitioner-related EAs score was 0/6 in 28 studies (8.9%), 1/6 in 40 studies (12.7%), 2/6 in 88 studies (27.9%), 3/6 in 112 studies (35.6%), 4/6 in 27 studies (8.6%), 5/6 in 20 studies (6.3%), all of them were acupressure studies. No studies score 6/6 (Table 4).

**Table 4:** Practitioner-related equality assumption score

	**Acu** (*n* = 77)	**Mas** (*n* = 8)	**Rei** (*n* = 2)	**Ref** (*n* = 20)	**TT** (*n* = 3)	**Mob** (*n* = 109)	**Man** (*n* = 92)	**Mix** (*n* = 4)	**Total** (*n* = 315)
*0/6*	10 (12.3%)	1 (12.5%)	0 (0%)	0 (0%)	0 (0%)	11 (10.2%)	6 (6.5%)	0 (0%)	28 (8.9%)
*1/6*	8 (10.4%)	2 (25.0%)	0 (0%)	5 (25.0%)	0 (0%)	12 (11.0%)	13 (14.1%)	0 (0%)	40 (12.7%)
*2/6*	12 (15.6%)	2 (25.0%)	1 (50.0%)	4 (30.0%	0 (0%)	32 (29.4%)	35 (38.0%)	2 (50.0%)	88 (27.9%)
*3/6*	19 (24.7%)	2 (25.0%)	1 (50.0%)	11 (55.0%)	2 (66.7%)	47 (43.1%)	28 (30.4%)	2 (50.0%)	112 (35.6%)
*4/6*	10 (13.0%)	1 (12.5%)	0 (0%)	0 (0%)	1 (33.3%)	6 (5.5%)	9 (9.8%)	0 (0%)	27 (8.6%)
*5/6*	18 (23.4%)	0 (0%)	0 (0%)	0 (0%)	0 (0%)	1 (0.9%)	1 (1.1%)	0 (0%)	20 (6.3%)

*Acu* Acupressure, *Mas* Massage, *Rei* Reiki, *TT* Therapeutic Touch, *Mob* Mobilisation, *Man* Manipulation, *Mix* Mixed-Method

No studies scored 6/6

## Discussion

The present review aimed to systematically report the similarity of non-specific factors between experimental and placebo arms in mtRCTs. Our results showed that there is a general lack of patient- and practitioner- related EA reporting. In contrast, the context-related EA items are well described. Among the patients’ characteristics analysed under the macro-area of patient-related EAs, patients’ expectations are the most decisive element [46, 47], Indeed, it has been demonstrated in physical therapy that expectation could influence clinical outcomes in patients suffering from musculoskeletal pain, in particular neck pain [48], low back pain [45] and cumulative trauma disorders [49]. Despite the availability of expectancy questionnaires [50], the present findings showed that expectancy effects had been considered in only 8.3% of the studies.

Previous experiences highly mediate expectancy in various ways: previous effective active treatments showed a higher likelihood to elicit placebo response [51, 52], whereas ineffective results attenuate them [53, 54], patients with more prolonged treatment exposure showed more significant placebo or nocebo effect [53, 55]. Our results showed that 23% of the included studies investigated patients’ previous experiences, but the quasi-totality enrolled naive participants. Naivety was often related to the investigated technique and/or therapy, but this could be insufficient to ensure similarity between groups. For example, a positive experience with one form of manual therapy can trigger a placebo response when the subject is receiving another manual approach. Notably, generalisation seems to be a fundamental characteristic of conditioning where learning about a specific treatment cue can generalise to other similars [53]. Although there are no validated tools accurately assessing patients’ previous experiences, the latter could be appraised through precise questions (e.g., “have you ever been treated with manual therapy?”; “if so, which one and what kind of experience did you have?”).

However, some authors are currently challenging the importance of expectation and previous experiences in exerting the placebo effect, using the models of prediction and error processing and Bayesian brain: placebo effects appear to be strongly influenced by “what you do, and only secondarily, or not at all, by what you think” [56].

Although interesting, this new conceptual proposal is, as of today, only marginally relevant to the present review. Future developments of the theories and more consistent evidence could lead to an update of the suggestions for planning strategies to control for EAs.

According to the literature, treatment credibility is the measure by which patients believe the intervention to be able to modify illness [26, 57]. This, in turn, would affect their expectation [20] producing a definite functional improvement [58]. It has been found that the placebo response could depend more on patients’ perception than on treatment effect [54, 59, 60]. The present review found that only 7.9% of the studies took this aspect into account. It is possible to control for treatment credibility through deblinding procedures, already structured to control for the success of the blinding process [61]. Deblinding procedures were used only by 13.6% of studies.

Another key feature of placebo response is represented by the patients’ personality traits [62], both in pain [19, 54, 63, 64], and in non-pain paradigms [65, 66] (see Jaksis, et al*.*, 2012 [67]and Darragh, et al*.*, 2014 [65] for a comprehensive review on personality and placebo response). Our results showed that only 10.5% of studies accounted for the personality and psychological traits and state of participants, specifically massage, reflexology and therapeutic touch studies. This significant trend could be explained with a holistic mind–body perspective inherent in the respective disciplines, but also with a second consideration. These CAMs, being relatively new to the evidence-based paradigm, need to increase the level of clinical-based research to prove their effectiveness. We could speculate that researchers are more prone to control for factors that could impact the overall response to the therapy, to enhance the quality of trials. Given their importance in affecting the placebo response, psychological traits should be investigated in all therapies. There are, indeed, several questionnaires regarding traits [68, 69] and mood [70, 71] that could be used at baseline to ensure a homogeneous distribution of patients.

The second macro-area regards the context-related EAs and includes all characteristics of the intervention surrounding patient and operator, going from where the intervention took place to how often, how long, when the outcomes were assessed, which body areas were targeted, and the possible side-effects following the intervention,. Literature suggests that contextual stimuli [54, 72], associated environmental cues [73] and the context in general [74] are critical elements for the placebo response. Environment, architecture, and interior design could also modulate patients’ outcome (see Testa and Rossettini, 2016 for details [74]). Treatments are therefore required to be administered in the same setting [5], also considering the influence of the conditions of the room (i.e. temperature, humidity) on several biological outcome measures.

In addition to the physical context, the operative context can be relevant in determining the effect of a given therapy. The operative context could be described as a ritual, that is a series of formal, repetitive acts or behaviours [32] occurring in association with the therapeutic act. Rituals are essential in eliciting the placebo effect [17, 75, 76] both before, during and after the session. It is worth noting that the simple measurement (i.e. blood pressure readings) can act as treatment [77, 78] and, therefore, a ritual can induce clinical effects.

Strong evidence supports these assumptions about context-related EAs; they are indeed included in the most common RCT guidelines (i.e., CONSORT), making them an already essential part of the study design. Our results showed in fact that the context-related EAs were generally considered more than EAs items relating to the patient and the practitioner. An exception is represented by the reporting of side effects, present only in 23.2% of studies. This prevents, in part, the ability to evaluate the similarity between groups. We could speculate that the presence of side effects could modify the placebo response in patients. For example, side effects could be interpreted as a signal to be part of the experimental treatment group (regardless if it is true or not), and so affect the outcomes.

The better reporting of context-related items seems in contrast with the results of a methodological review by Alvarez and colleagues, [23], that showed a lack of improvements in the methodology of MT trials comparing before and after the publication of CONSORT. With respect to the systematic review of Alvarez and colleagues [23], we analysed studies based on a conceptual paradigm (equality assumption), and we only included studies with at least one sham manual control. Furthermore, there is a distinction between the type of review applied as per methodological vs systematic. The third and last macro-area concerns the role of the practitioner, which is essential for both specific and non-specific effects of therapy. The doctor has been called “a powerful therapeutic agent” and both a “practitioner effect” [79] and a “physiotherapist’s effect” have been estimated in patients with musculoskeletal disorders [74]. It has been argued that a placebo effect is a form of the therapeutic alliance [80], The therapeutic alliance, defined as a working relationship or a positive social connection between the patient and the therapist [81], is particularly relevant in manual therapies. Therapists could shape the placebo response in several ways. For example, the physician’s enthusiasm would result in a significantly higher effect on the patient response [77, 81], communication seems to have a crucial role in eliciting placebo response [72], patients’ perception of the operator’s expertise, professionalism and reputation is of significance in modifying clinical outcomes [74, 82, 83]. It is unclear whether age and gender of the treating practitioner can influence the placebo response [84]. It may, therefore, represent a confounding factor for the treatment effectiveness, when treatments are delivered by different therapists [85].

Our results showed that approximately 80% of the included studies reported the type and the number of operators; whereas their experience, training, gender and age were underreported. To ensure the practitioner-related EAs, it should be recommended that each operator performs the same number of treatments in both experimental and sham intervention; it is advisable to provide training for the practitioners, aimed not only at a homogeneous execution of experimental and sham techniques but also at defining verbal and nonverbal communication with the patient. A useful and valid tool to control for these variables in MT could be the TIDieR checklist [86], as suggested by Alvarez et al. [23].

Training in performing the sham technique is fundamental. The operator should pay attention to avoid any specificity in sham treatments. Sham procedures should be tailored to the therapeutic approach or technique it mimics, as some researchers have already done [30, 61].

86 studies declared to have a double-blind design, borrowing the expression from pharmacological research, in which “both sides” of treatment administration (i.e., patient and clinician) do not know whether the active principle is present in a given drug. On the contrary, in MT scenario, it is impossible to blind who administers the treatment. So, if a second person is blinded (e.g., data analyst, data collector, or outcome assessor) the expression “dual blind” should be preferred [87]).

There are many factors to consider when planning an mtRCT and a large number of tools available to do so. The evidence-based panorama is vast and can be dispersive, even more so in the absence of clear guidelines. It would be useful and efficient to unify the existing tools in a comprehensive, shared and specific checklist for MT. This would offer a structured step-by-step guide giving researchers the possibility to improve the areas that need adjustments, and thus increasing the likelihood of obtaining valid, generalisable and robust results.

### Limitations

We acknowledge some limitations. Firstly, despite the effort to identify all relevant literature, the search strategy may have left out some studies. Secondly, we did not take into account some EA items that may influence the placebo response and should be taken into consideration when planning a sham therapy. Particularly the operators’ empathy [88], the characteristics of the interaction between operators and participants (both verbal [51]) and nonverbal [31]) that can modify the patient perception of the therapy believability [44], including the eventual training of operator aimed at the style of rapport with the patient, and quality of patient-operator interaction [20, 26] through a satisfaction questionnaire. Finally, although a protocol similar to Cerritelli et al. [16] was followed, an a priori protocol for this methodological review has not been published.

## Conclusions

This review showed a moderate quality in the reporting of context-related EA items, potentially because they are primarily included in pre-existent guidelines. In contrast, there is a general lack of attention to the patient- and practitioner- related EAs, that could be controlled through already existing tools. Poor planning and reporting might limit the robustness of the EA, and the validity of the evidence.

## Supplementary Information

**Additional file 1.**

## Data Availability

The datasets used and/or analysed during the current study are available from the corresponding author on reasonable request.

## Abbreviations

EA: Equality assumption
MT: Manual therapy
RCT: Randomised controlled trials
mtRCT: Randomised controlled trials of manual therapy
CAMs: Complementary alternative medicines
PROMs: Patient-reported outcome measures
Acu: Acupressure
Mas: Massage
Rei: Reiki
TT: Therapeutic touch
Mob: Mobilisation
Man: Manipulation
Mix: Mixed-method
N: Number
