# Correction: Differences between experimental and placebo arms in manual therapy trials: a methodological review

**DOI:** 10.1186/s12874-023-01865-0

**Published:** 2023-02-21

**Authors:** Giandomenico D’Alessandro, Nuria Ruffini, Alessandro Aquino, Matteo Galli, Mattia Innocenti, Marco Tramontano, Francesco Cerritelli

**Affiliations:** 1 Clinical-Based Human Research Department, Foundation C.O.ME. Collaboration, 65121 Pescara, Italy; 2 Centre Pour L’Etude, La Recherche Et La Diffusion Ostéopathiques “C.E.R.D.O”, 00199 Rome, Italy; 3 Foundation C.O.ME. Collaboration, National Centre Germany, 10825 Berlin, Germany; 4 Department of Health Sciences, University of Milan, 20142 Milan, Italy; 5 Research Department, SOMA, Istituto Osteopatia Milano, Milan, Italy; 6 Fondazione Santa Lucia IRCCS, 00179 Rome, Italy

**Correction: BMC Medical Research Methodology 22, 219 (2022)**

**https://doi.org/10.1186/s12874-022-01704-8**

Following publication of the original article [1], the authors reported an error in the presentation of author names. The given name and family name were swapped. The correct author names are as follows:

Giandomenico D’Alessandro

Nuria Ruffini

Alessandro Aquino

Matteo Galli

Mattia Innocenti

Marco Tramontano

Francesco Cerritelli

The author group has been updated above and the original article [1] has been corrected.
