# Supplementary material for: Differences between experimental and placebo arms in manual therapy trials: a methodological review
Source: BMC Med Res Methodol. 2022 Aug 8;22:219. doi: 10.1186/s12874-022-01704-8 (PMC9358888; doi:10.1186/s12874-022-01704-8)
Supplement: Supplementary file 1 — Additional file 1. [file 12874_2022_1704_MOESM1_ESM.pdf]

Differences between experimental and placebo arms in Manual Therapy trials: a Systematic Review

D'Alessandro Giandomenico[a,b*], Ruffini Nuria[a, c], Tramontano Marco[d], Aquino Alessandro[a,e], Galli Matteo[a,f], Innocenti Mattia[b], Cerritelli Francesco[a*]

[a] Clinical-Based Human Research Department, Foundation C.O.ME. Collaboration, Pescara, 65121, Italy

[b] Centre pour l'Etude, la Recherche et la Diffusion Ostéopathiques "C.E.R.D.O". Rome, 00199, Italy.

[c] National Centre Germany, Foundation C.O.ME. Collaboration, Berlin, 10825, Germany.

[d] Fondazione Santa Lucia IRCCS, Rome, 00179, Italy.

[e] Department of Health Sciences, University of Milan, Milan, 20142, Italy.

[f] Research Department, SOMA, Istituto Osteopatia Milano, Milan, Italy.

*Corresponding author
Francesco Cerritelli

fcerritelli@comecollaboration.org

Supplementary information S1

Search Strategy

(placebo) OR (sham)

(sham OR placebo) AND (“manual medicine” OR “manual medicines” OR “manual therapy” OR “manual therapies” OR “physiotherap*” OR “chiropractic” OR “spinal manipulative treatment” OR “SMT” OR “massage” OR “acupressure” OR “ANMA” OR “shiatsu” OR “applied kinesiology” OR “Alexander technique” OR “Feldenkrais” OR “Bowen technique” OR “Hakomi” OR “Reflexology” OR “Rolfing structural integration” OR “Somatic experiencing” OR “Trager approach” OR “physical therapy” OR “physical therapies” OR “bone setter” OR “bone setting” OR “dorn method” OR “integrative manipulative treatment” OR “joint manipulation” OR “joint mobilization” OR “spinal mobilization” OR “spinal manipulation” OR “manual lymphatic drainage” OR “muscle energy technique” OR “myofascial release” OR “myofascial therapy” OR “myofascial therapies” OR “myofascial treatment” OR “myofascial treatments” OR “Myotherapy” OR “Naprapathy” OR “polarity therapy” OR “Seitai” OR “sotai” OR “Structural integration” OR “Traction” OR “Tuin na” OR “Zero balancing” OR “counterstrain” OR “Orthopedic Manipulation” OR “Soft Tissue Therapy” OR “Continuous Passive Motion Therapy” OR “Musculoskeletal Manipulations”)

(sham OR placebo) AND (manual medicine OR manual medicines OR manual therapy OR manual therapies OR physiotherap* OR chiropractic OR spinal manipulative treatment OR SMT OR massage OR acupressure OR ANMA OR shiatsu OR applied kinesiology OR Alexander technique OR Feldenkrais OR Bowen technique OR Hakomi OR Reflexology OR Rolfing structural integration OR Somatic experiencing OR Trager approach OR physical therapy OR physical therapies OR bone setter OR bone setting OR dorn method OR integrative manipulative treatment OR joint manipulation OR joint mobilization OR spinal mobilization OR spinal manipulation OR manual lymphatic drainage OR muscle energy technique OR myofascial release OR myofascial therapy OR myofascial therapies OR myofascial treatment OR myofascial treatments OR Myotherapy OR

Naprapathy OR polarity therapy OR Seitai OR sotai OR Structural integration OR Traction OR Tuin na OR Zero balancing OR counterstrain OR Orthopedic Manipulation OR Soft Tissue Therapy OR Continuous Passive Motion Therapy OR Musculoskeletal Manipulations)

Key Words

"manual medicine"

"manual medicines"

"manual therapy"

"manual therapies"
"manual"

"physiotherapy*"

"chiropractic"

"spinal manipulative treatment"
"SMT"

"massage"

"acupressure"

"ANMA"

"shiatsu"

"applied kinesiology"

“Alexander technique”

“Feldenkrais”

“Bowen technique”

“Hakomi”

“Reflexology”
“Rolfing structural integration”

“Somatic experiencing”

“Trager approach”

“physical therapy”

“physical therapies”

“bone setter”

“bone setting”

“dorn method”

“integrative manipulative treatment”

“joint manipulation”

“joint mobilization”

“spinal mobilization”

“spinal manipulation”

“manual lymphatic drainage”

“muscle energy technique”

“myofascial release”

“myofascial therapy”

“myofascial therapies”

“myofascial treatment”

“myofascial treatments”

“Myotherapy”

“Naprapathy”

“polarity therapy”

“Seitai”
“sotai”

“Structural integration”

“Traction”

“Tuin na”

“Zero balancing”

“counterstrain”

“Orthopedic Manipulation”

“Soft Tissue Therapy”

“Continuous Passive Motion Therapy”

“Musculoskeletal Manipulations”

ID	Study ID	Study design	Intervention	Comparison	Primary Outcome	Secondary Outcome	Decision	Notes
	Author_year - title	Multi-RCT, RCT, ITS, CBA	At least 1 Manual intervention	Any kind of comparison	Manual intervention vs Sham Therapy	Data on side effects	Y/N	
1	Abaraogu_2015 - As Acupressure Decreases Pain, Acupuncture May Improve Some Aspects of Quality of Life for Women with Primary Dysmenorrhea: A Systematic Review with Meta-Analysis	N	Y	Y	Y	Y	N	
2	Abbasi_2015 - The effect of acupressure on constipation in patients with chronic renal failure undergoing hemodialysis	Y	Y	Y	Y	Y	N	arabic: first mail to corresponding author on 06.11.2017, second mail on the 20.06.2017, no answer

**3**	Abbien-Denton_1982 - Physiotherapy in the treatment of hyperkinetic children	N	Y	N	N	N	N	
**4**	Abbott_2016 - Clinical predictors of response to exercise & manual therapy interventions for patients with hip osteoarthritis: A independent validation study	N	Y	Y	N	N	N	Comparison between 2 RCTs
**5**	Abdulla_2015 - Is exercise effective for the management of subacromial impingement syndrome and other soft tissue injuries of the shoulder? A systematic review by the Ontario Protocol for Traffic Injury Management (OPTIMa) Collaboration	N	N	N	N	N	N	
**6**	**Abedian_2015 - The Effect of Acupressure on Sleep Quality in Menopausal Women**	**Y**	**Y**	**Y**	**Y**	**N**	**Y**	
**7**	Abu_2015 - The effectiveness of manual versus algometer pressure release techniques for treating active myofascial trigger points of the upper trapezius.	Y	Y	Y	N	N	N	
**8**	Adlan_2015 - Acupressure as an adjunct treatment for inpatient management of nausea and vomiting in early pregnancy: A double-blind-randomized-controlled trial.	Y	N	Y	N	N	N	
**9**	Agarwal_2002 - Acupressure and ondansetron for postoperative nausea and vomiting after laparoscopic cholecystectomy.	Y	N	Y	N	N	N	

**10**	**Agarwal_2005 - Acupressure for prevention of pre-operative anxiety a prospective, randomised, placebo controlled study**	**Y**	**Y**	**Y**	**Y**	**N**	**Y**
**11**	Aghadam_2010 - Evaluation of the effects of acupressure by sea band on nausea and vomiting of pregnancy.	Y	N	Y	N	N	N
**12**	Aguilera_2009 - immediate effect of ultrasound and ischemic compression Techniques for the Treatment of Trapezius Latent Myofascial Trigger Points in Healthy Subjects: A Randomized Controlled Study	Y	Y	Y	N	N	N
**13**	Ahles_1999 - Massage Therapy for Patients Undergoing Autologous Bone Marrow Transplantation.	Y	Y	Y	N	N	N
**14**	Ajimsha_2015 - Effectiveness of myofascial release: Systematic review of randomized controlled trials.	N	Y	Y	N	N	N
**15**	Ajimsha_2014 - Effectiveness of myofascial release in the management of plantar heel pain: a randomized controlled trial.	Y	Y	Y	N	N	N
**16**	Ajimsha_2012 - Effectiveness of myofascial release in the management of lateral epicondylitis in computer professionals	Y	Y	Y	N	N	N
**17**	**Ajimsha_2014 - Effectiveness of myofascial release in the management**	**Y**	**Y**	**Y**	**Y**	**N**	**Y**

	**of chronic low back pain in nursing professionals.**						
**18**	Albacete-García_2011 - Terapia manual en la epicondilitis: una revisión sistemática de ensayos clínicos	N	Y	Y	Y	N	N
**19**	Alburquerque-Sendìn_2007 - Immediate effects of bilateral manipulation of talocrural joints on Standing Stability in Healthy Subjects	Y	Y	Y	N	N	N
**20**	Alcantara_2011 - The chiropractic care of infants with colic: A systematic review of the literature	N	Y	Y	Y	N	N
**21**	Alcantara_2012 - The chiropractic care of patients with asthma: A systematic review of the literature to inform clinical practice.	N	Y	Y	Y	N	N
**22**	Alcantara_2011 - A Systematic Review of the Literature on the Chiropractic Care of Patients with Autism Spectrum Disorder.	N	Y	Y	Y	N	N
**23**	Alcantara_2012 - what is the evidence for chiropractic management of infantile colic? Further considerations.	N	N	N	N	N	N
**24**	Alessandrini_2012 - P6 acupressure effectiveness on acute vertiginous patients: a double blind randomized study.	Y	N	Y	N	N	N
**25**	Alkaissi_2002 - P6 acupressure may relieve nausea and vomiting after	Y	N	Y	N	N	N

	gynecological surgery: an effectiveness study in 410 women							
**26**	Alkaissi_1999 - effect and placebo effect of acupressure (P6) on nausea and vomiting after outpatient gynaecological surgery	Y	N	Y	N	N	N	
**27**	Allen_1994 - P6 acupressure and nausea and vomiting after gynaecological surgery	Y	N	Y	N	N	N	acupressure wristbands
**28**	Allen_2008 - P6 stimulation for the prevention of nausea and vomiting associated with cesarean delivery under neuraxial anesthesia: A systematic review of randomized controlled trials	Y	Y	Y	Y	N	N	
**29**	Alper_2011 - Tai chi, glucosamine, probiotics, acupressure, and pelargonium sidoides	N	N	N	N	N	N	
**30**	Alraek_2011 - Complementary and alternative medicine for patients with chronic fatigue syndrome: A systematic review.	N	N	N	N	N	N	
**31**	Aly_2004 - Physical activity combined with massage improves bone mineralization in premature infants: a randomized trial.	Y	Y	Y	N	N	N	
**32**	Ammer_2014 - Pain reduction by placebo manipulation versus real manual therapy	Y	Y	Y	Y	Y	N	Comment
**33**	Amro_2010 - The effects of Mulligan mobilisation with movement and taping	Y	Y	N	Y	N	N	no manual control

	techniques on pain, grip strength, and function in patients with lateral epicondylitis.							
**34**	Anaya-Terroba_2010 - Effects of Ice Massage on Pressure Pain Thresholds and Electromyography Activity Postexercise: A Randomized Controlled Crossover Study	Y	Y	Y	N	N	N	
**35**	Andersen_2012 - The effect of stimulation therapy and donepezil on cognitive function in Alzheimer's disease. A community based RCT with a two-by-two factorial design.	Y	N	Y	N	N	N	
**36**	Anderson_1992 - A meta-analysis of clinical trials of spinal manipulation.	N	Y	Y	Y	N	N	
**37**	Ang_2012 - A randomized placebo-controlled trial of massage therapy on the immune system of preterm infants	Y	Y	Y	N	N	N	without physical contact
**38**	Antolinos-Campillo_Short-term changes in median nerve neural tension after a suboccipital muscle inhibition technique in subjects with cervical whiplash: A randomised controlled trial	Y	Y	Y	Y	N	N	osteopathy
**39**	Aoyagi_2015 - Determining the level of evidence for the effectiveness of spinal manipulation in upper limb pain: A systematic review and meta-analysis	N	Y	Y	Y	N	N	
**40**	**Aparicio_2009 - Immediate effects of the suboccipital muscle inhibition technique in subjects with short hamstring syndrome.**	**Y**	**Y**	**Y**	**Y**	**N**	**Y**	

**41**	**Arab_2016 - A sham-controlled trial of acupressure on the quality of sleep and life in haemodialysis patients**	**Y**	**Y**	**Y**	**Y**	**N**	**Y**	
**42**	**Arai_2011 - The influence of acupressure at extra 1 acupuncture point on the spectral entropy of the EEG and the LF/HF ratio of heart rate variability**	**Y**	**Y**	**Y**	**Y**	**Y**	**Y**	
**43**	**Arai_2008 - The effect of acupressure at the extra 1 point on subjective and autonomic responses to needle insertion.**	**Y**	**Y**	**Y**	**Y**	**Y**	**Y**	
**44**	Arguisuelas Martínez_2010 - Efectos de la manipulación lumbar y técnica de inducción miofascial toracolumbar sobre el patrón de activación del erector espinal.	Y	Y	Y	Y	N	N	comparison
**45**	**Arguisuelas-Martinez_2016 - Effects of myofascial release in non-specific chronic low back pain: A randomized clinical trial**	**Y**	**Y**	**Y**	**Y**	**N**	**Y**	
**46**	Armijo-Olivo_2016 - Effectiveness of Manual Therapy and Therapeutic Exercise for Temporomandibular Disorders: Systematic Review and Meta-Analysis	N	Y	Y	Y	Y	N	
**47**	Arroyo-Morales-Cantarero-Villanueva_2012 - Effectiveness of core stability exercises and recovery myofascial release massage on fatigue	Y	Y	Y	Y	N	N	

	in breast cancer survivors: A randomized controlled clinical trial							
**48**	Arroyo-Morales_2008 - Psychophysiological effects of massage-myofascial release after exercise: a randomized sham-control study	Y	Y	Y	Y	N	N	
**49**	Arroyo-Morales_2009 - Massage after exercise-responses of immunologic and endocrine markers: a randomized single-blind placebo-controlled study.	Y	Y	Y	Y	N	N	
**50**	Asher_1990 - Effects of chest physical therapy on lung function in children recovering from acute severe asthma	Y	Y	Y	N	N	N	
**51**	Askari_2016 - Effect of back massage with sesame oil on pain and length of delivery in primiparous women."	Y	Y	Y	Y	Y	N	the only difference is the oil
**52**	Asplund_2003 - Manual lymph drainage therapy using light massage for fibromyalgia sufferers: a pilot study	N	Y	N	N	N	N	
**53**	**Assefi_2008 - Reiki for the treatment of fibromyalgia: a randomized controlled trial**	**Y**	**Y**	**Y**	**Y**	**N**	**Y**	
**54**	Assendelft_2013 - WITHDRAWN: Spinal manipulative therapy for low-back pain	N	Y	Y	Y	N	N	
**55**	Atkinson_2001 - The relative effectiveness of combined spinal manipulative therapy and "action potential" therapy versus combined	Y	Y	Y	N	N	N	

	spinal manipulative therapy and placebo "action potential" therapy in the treatment of mechanical low back pain							
**56**	Atkinson_2008 - A randomized controlled trial to assess the efficacy of shoulder manipulation versus placebo in the treatment of shoulder pain due to rotator cuff tendinopathy	Y	Y	Y	N	N	N	
**57**	Atkinson_2002 - Relative effectiveness of combined spinal manipulative therapy and 'action potential' therapy versus combined spinal manipulative therapy and placebo 'action potential' therapy in the treatment of mechanical low back pain	Y	Y	Y	N	N	N	
**58**	Atrian_2013 - Investigatingthe effect of pressure on third liver point on primary dysmenorrhea: A randomized controlled clinical trial.	Y	Y	Y	Y	N	N	they use an instrument for the pressure
**59**	**Aytar_2015 - The effects of scapular mobilization in patients with subacromial impingement syndrome: a randomized, double-blind, placebo-controlled clinical trial**	**Y**	**Y**	**Y**	**Y**	**N**	**Y**	
**60**	Bablis_2011 - A randomized controlled trial of neuro-emotional technique for low back pain.	Y	Y	Y	Y	N	N	NET
**61**	Bablis_2008 - Neuro Emotional Technique for the treatment of trigger point sensitivity in chronic neck pain sufferers: a controlled clinical trial	Y	Y	Y	Y	N	N	NET

**62**	Baghani_2015 - Postoperative effects of reflexology on the physiological parameters of patients with appendicitis	Y	N	Y	N	Y	N	first mail sent to corrsponding author on 16/11/2017. I received the manuscript, the paper is actually Khorsand_2015, which was excluded
**63**	Baillergeau_2012 - Comparaison des effets d'une manipulation de l'articulation crânio-cervicale et du rachis thoracique sur la distance interincisives	Y	Y	Y	N	N	N	
**64**	Bakar_2014 - Short Term Effects of Classic Massage Compared to Connective Tissue Massage on Pressure Pain Threshold and Muscle Relaxation Response in Women With Chronic Neck Pain: A Preliminary Study	Y	Y	Y	N	N	N	
**65**	Bakhtshirin_2015 - The effect of aromatherapy massage with lavender oil on severity of primary dysmenorrhea in Arsanjan students	Y	Y	Y	N	N	N	The difference between groups is the oil
**66**	**Bakris_2007 - Atlas vertebra realignment and achievement of arterial pressure goal in hypertensive patients: a pilot study**	**Y**	**Y**	**Y**	**Y**	**N**	**Y**	
**67**	Baldwin_2013 - Comparison of physical therapy with energy healing for improving range of motion in subjects with restricted shoulder mobility	Y	Y	Y	N	N	N	no touch in sham procedure

**68**	Bale_2005 - Chiropractic for neck pain: A pilot study examining whether the duration of the pain affects the clinical outcome	N	Y	N	N	N	N	
**69**	Balthazard_2011 - Effets d’un traitement combiné de thérapie manuelle suivie d’exercices actifs sur l’amélioration de l’incapacité fonctionnelle de patients lombalgiques chroniques.	Y	Y	Y	N	N	N	placebo = ultrasound
**70**	Balthazard_2012 - Manual therapy followed by specific active exercises versus a placebo followed by specific active exercises on the improvement of functional disability in patients with chronic non specific low back pain: a randomized controlled trial	Y	Y	Y	N	N	N	
**71**	Bao_2011 - The analgesic effect of magnetic acupressure in cancer patients undergoing bone marrow aspiration and biopsy: a randomized, blinded, controlled trial.	Y	N	Y	N	N	N	
**72**	Barbic_2014 - Effects of mechanical stimulation of the feet on gait and cardiovascular autonomic control in Parkinson's disease	Y	N	Y	N	N	N	use of a steel stick with a smooth
**73**	Barra López_2013 - Effectiveness of Diacutaneous Fibrolysis for the treatment of subacromial impingement syndrome: a randomised controlled trial	Y	N	Y	N	N	N	

**74**	Barrett_2016 - Is thoracic spine posture associated with shoulder pain, range of motion and function? A systematic review	N	N	N	N	N	N	
**75**	**Bastani_2015 - Effect of Acupressure on Maternal Anxiety in Women With Gestational Diabetes Mellitus: A Randomized Clinical Trial**	**Y**	**Y**	**Y**	**Y**	**N**	**Y**	
**76**	Bastani_2015 - Effect of foot reflexology on anxiety of mothers of high risk neonates undergone cesarean section: A non randomized controlled trial	Y	Y	Y	Y	N	N	no translation available
**77**	**Bastani_2015 - The effect of acupressure on cancer-related fatigue among school-aged children with acute lymphoblastic leukemia**	**Y**	**Y**	**Y**	**Y**	**N**	**Y**	
**78**	**Bastani_2015 - Effect of acupressure on fatigue in women with multiple sclerosis.**	**Y**	**Y**	**Y**	**Y**	**N**	**Y**	
**79**	Batalha_2013 - Massage in children with cancer: effectiveness of a protocol	Y	Y	Y	Y	N	N	control group has only usual care
**80**	Bay_2011 - Combined therapy using acupressure therapy, hypnotherapy, and transcendental meditation versus placebo in type 2 diabetes.	Y	Y	Y	N	N	N	No manual placebo
**81**	Bayreuther_1994 - A double-blind cross-over study to evaluate the effectiveness of acupressure at	Y	N	Y	N	N	N	

	pericardium 6 (P6) in the treatment of early morning sickness (EMS)							
**82**	Bazarganipour_2010 - A randomized clinical trial of the efficacy of applying a simple acupressure protocol to the Taichong point in relieving dysmenorrhea.	Y	N	Y	N	N	N	
**83**	Belluomini_1994 - Acupressure for nausea and vomiting of pregnancy: a randomized, blinded study	Y	Y	Y	Y	N	N	mail not found, paper not found
**84**	Bennell_2010 - Efficacy of standardised manual therapy and home exercise programme for chronic rotator cuff disease: randomised placebo controlled trial	Y	Y	Y	N	N	N	Application of gel as sham + detuned ultrasound
**85**	Bennell_2014 - Effect of physical therapy on pain and function in patients with hip osteoarthritis: a randomized clinical trial	Y	Y	Y	N	N	N	Application of gel as sham + detuned ultrasound
**86**	Bennell_2010 - Efficacy of a multimodal physiotherapy treatment program for hip osteoarthritis: a randomised placebo-controlled trial protocol	Y	Y	Y	N	N	N	Application of gel as sham + detuned ultrasound
**87**	Bennell_2005 - Efficacy of physiotherapy management of knee joint osteoarthritis: a randomised, double blind, placebo controlled trial.	Y	Y	Y	N	N	N	Application of gel as sham + detuned ultrasound
**88**	Bervoets_2015 - Massage therapy has short-term benefits for people with common musculoskeletal disorders	N	Y	Y	Y	N	N	

	compared to no treatment: a systematic review							
**89**	**Beselga_2016 - Immediate effects of hip mobilization with movement in patients with hip osteoarthritis: A randomised controlled trial**	**Y**	**Y**	**Y**	**Y**	**N**	**Y**	
**90**	Beurskens_1996 - The Efficacy of Lumbar Traction: Design and results of a randomised clinical trial	Y	N	Y	Y	N	N	
**91**	Beurskens_1995 - The efficacy of traction for lumbar back pain: design of a randomized clinical trial	Y	N	Y	Y	N	N	
**92**	Beurskens_1995 - Efficacy of traction for non-specific low back pain: a randomised clinical trial	Y	N	Y	Y	N	N	
**93**	Beurskens_1997 - Efficacy of traction for nonspecific low back pain. 12-week and 6-month results of a randomized clinical trial	Y	N	Y	Y	N	N	
**94**	Bialosky_2011 - Placebo response to manual therapy: Something out of nothing?	N	N	N	N	N	N	conceptual paper
**95**	**Bialosky_2009 - A randomized sham-controlled trial of a neurodynamic technique in the treatment of carpal tunnel syndrome**	**Y**	**Y**	**Y**	**Y**	**N**	**Y**	
**96**	**Bialosky_2014 - Spinal Manipulative Therapy–Specific Changes in Pain Sensitivity in Individuals With Low Back Pain**	**Y**	**Y**	**Y**	**Y**	**N**	**Y**	

**97**	Bishop_2010 - The Chiropractic Hospital-based Interventions Research Outcomes (CHIRO) Study: a randomized controlled trial on the effectiveness of clinical practice guidelines in the medical and chiropractic management of patients with acute mechanical low back pain	Y	Y	Y	N	N	N	
**98**	Blanchette_2011 - Augmented Soft Tissue Mobilization vs Natural History in the Treatment of Lateral Epicondylitis: A Pilot Study	Y	N	Y	N	N	N	
**99**	**Blankfield_2001 - Therapeutic touch in the treatment of carpal tunnel syndrome**	**Y**	**Y**	**Y**	**Y**	**N**	**Y**	
**100**	Bliddal_2015 - Spiritual healing in the treatment of rheumatoid arthritis: An exploratory single centre, parallel-group, double-blind, three-arm, randomised, sham-controlled trial	Y	N	Y	N	N	N	
**101**	Blikstad_2008 - "Immediate effect of activator trigger point therapy and myofascial band therapy on non-specific neck pain in patients with upper trapezius trigger points compared to sham ultrasound: A randomised controlled trial	Y	N	Y	N	N	N	
**102**	Boehler_2002 - Korean hand acupressure reduces postoperative nausea and vomiting after gynecological laparoscopic surgery	Y	N	Y	N	N	N	

**103**	Boët_2013 - High-velocity low-amplitude thrust manipulation of the lumbar spine immediately modifies soleus T reflex in asymptomatic adults	Y	Y	Y	Y	N	N	osteopathy
**104**	Boline_1995 - Spinal manipulation versus amitriptyline for the treatment of chronic tension-type headaches: a randomized clinical trial	Y	Y	Y	N	N	N	
**105**	Borges_2014 - Occupational low back pain in nursing workers: massage versus pain	Y	Y	Y	N	N	N	Application of gel as sham
**106**	Borimnejad_2012 - The effects of acupressure on preoperative anxiety reduction in school aged children	Y	N	Y	N	Y	N	*"an acupressure bead attached to a self-adhesive tape"*
**107**	**Borusiak_2010 - Lack of efficacy of manual therapy in children and adolescents with suspected cervicogenic headache: results of a prospective, randomized, placebo-controlled, and blinded trial.**	**Y**	**Y**	**Y**	**Y**	**N**	**Y**	
**108**	**Botelho_2011 - Effect of cervical spine manipulative therapy on judo athletes' grip strength**	**Y**	**Y**	**Y**	**Y**	**N**	**Y**	
**109**	Bove_1998 - Spinal manipulation in the treatment of episodic tension-type headache: a randomized controlled trial	Y	Y	Y	Y	N	N	sham laser
**110**	Boyd_2016 - The Impact of Massage Therapy on Function in Pain Populations-A Systematic Review and Meta-Analysis of Randomized	N	N	N	N	N	N	

	Controlled Trials: Part II, Cancer Pain Populations.							
**111**	Brantingham_2005 - A pilot study of the efficacy of a conservative chiropractic protocol using graded mobilization, manipulation and ice in the treatment of symptomatic hallux abductovalgus bunion.	Y	Y	Y	N	N	N	
**112**	Bretischwerdt_2010 - Immediate Effects of Hamstring Muscle Stretching on Pressure Pain Sensitivity and Active Mouth Opening in Healthy Subjects	Y	Y	Y	Y	N	N	unilateral, bilateral and no intervention
**113**	**Briem_2007 - Immediate effects of inhibitive distraction on active range of cervical flexion in patients with neck pain: A pilot study**	**Y**	**Y**	**Y**	**Y**	**N**	**Y**	
**114**	Brill_1995 - Acupressure for nausea and vomiting of pregnancy: A randomized, blinded study	Y	Y	Y	Y	Y	N	comment
**115**	**Bronfort_2001 - Chronic pediatric asthma and chiropractic spinal manipulation: a prospective clinical series and randomized clinical pilot study**	**Y**	**Y**	**Y**	**Y**	**Y**	**Y**	
**116**	Bronfort_1993 - A randomized placebo controlled clinical trial of chiropractic spinal adjustive therapy versus use of parental guideline for appropriate parent/infant interaction	Y	Y	Y	N	N	N	

**117**	Bronfort_2001 - A randomized clinical trial of exercise and spinal manipulation for patients with chronic neck pain	Y	Y	N	N	N	N	there is no placebo/control
**118**	Bronfort_1996 - Trunk exercise combined with spinal manipulative or NSAID therapy for chronic low back pain: a randomized, observer-blinded clinical trial.	Y	Y	Y	N	Y	N	no manual control
**119**	**Brooks_2005 - The immediate effects of manual massage on power-grip performance after maximal exercise in healthy adults**	**Y**	**Y**	**Y**	**Y**	**N**	**Y**	
**120**	**Brown_2015 - A biopsychosocial approach to primary hypothyroidism: Treatment and harms data from a randomized controlled trial**	**Y**	**Y**	**Y**	**Y**	**N**	**Y**	
**121**	Brown_2008 - Reflexology treatment for patients with lower limb amputations and phantom limb pain—An exploratory pilot study	N	Y	N	N	N	N	
**122**	Browning_2008 - Comparison of the short-term effects of chiropractic spinal manipulation and occipito-sacral decompression in the treatment of infant colic: A single-blinded, randomised, comparison trial."	Y	Y	Y	N	N	N	
**123**	Bruyneel_2015 - Efficacité de l'étirement du triceps sural sur une plate-forme oscillante motorisée par	Y	Y	Y	Y	Y	N	presented at a congress (published paper not found) but also comparison between one exercise and massage

	rapport à un contracté-relâché manuel et un «placebo»							
**124**	Buchbinder_2007 - Efficacy and cost-effectiveness of physiotherapy following glenohumeral joint distension for adhesive capsulitis: a randomized trial.	Y	Y	Y	N	N	N	
**125**	**Buchmann_2005 - Manual treatment effects to the upper cervical apophysial joints before, during, and after endotracheal anesthesia: a placebo-controlled comparison.**	**Y**	**Y**	**Y**	**Y**	**N**	**Y**	
**126**	**Budgell_2006 - The effects of thoracic manipulation on heart rate variability: a controlled crossover trial**	**Y**	**Y**	**Y**	**Y**	**N**	**Y**	
**127**	Buil Cosiales_2004 - El tratamiento fisioterapéutico convencional mejora más la sensación subjetiva de dolor que sólo el consejo, pero no la capacidad funcional ni la calidad de vida	Y	Y	Y	Y	N	N	no manual therapy
**128**	Buttagat_2011 - The immediate effects of traditional Thai massage on heart rate variability and stress-related parameters in patients with back pain associated with myofascial trigger points	Y	Y	Y	N	N	N	
**129**	Buttagat_2015 - Immediate effects of Thai massage on electromyogram and pain related parameters in patients with upper back pain associated with myofascial trigger points: A randomized controlled study.	Y	Y	Y	N	N	N	

**130**	Buttagat_2016 - Short-term effects of traditional Thai massage on electromyogram, muscle tension and pain among patients with upper back pain associated with myofascial trigger points.	Y	Y	Y	N	N	N	
**131**	Calixtre_2015 - Manual therapy for the management of pain and limited range of motion in subjects with signs and symptoms of temporomandibular disorder: A systematic review of randomised controlled trials	N	N	N	N	N	N	
**132**	Cambron_2014 - A pilot randomized controlled trial of flexion-distraction dosage for chiropractic treatment of lumbar spinal stenosis	Y	Y	Y	N	N	N	sham laser and handheld device to manipulate
**133**	Can Gürkan_2008 - Effect of acupressure on nausea and vomiting during pregnancy	Y	N	Y	N	N	N	
**134**	Can_2015 - Evaluation of the effects of ice massage applied to large intestine 4 (hegu) on postpartum pain during the active phase of labor	Y	Y	Y	N	N	N	
**135**	**Capó-Juan_ - Short term effectiveness of Pressure Release and Kinesiotaping in Cervical Myofascial Pain caused by sternocleidomastoid muscle: A randomized clinical trial**	**Y**	**Y**	**Y**	**Y**	**N**	**Y**	Algometric bilateral pressure as control (manual)
**136**	Capó-Juan_ - Short term effectiveness of Pressure Release and Kinesiotaping in Cervical Myofascial Pain caused by	Y	Y	Y	Y	N	N	same as ID135

	sternocleidomastoid muscle: A randomized clinical trial							
**137**	Carr_2015 - Effects of P6 Stimulation on Postoperative Nausea and Vomiting in Laparoscopic Cholecystectomy Patients	Y	Y	Y	N	N	N	
**138**	Cassidy_2003 - Methodological issues in investigations of massage/ bodywork therapy: Part IV: Experimental research designs	Y	Y	Y	Y	Y	N	
**139**	Castellote-Caballero_2013 - Effects of a neurodynamic sliding technique on hamstring flexibility in healthy male soccer players. A pilot study	Y	Y	Y	N	N	N	confronting two approaches
**140**	Castellote-Caballero_2014 - Immediate Effects of Neurodynamic Sliding versus Muscle Stretching on Hamstring Flexibility in Subjects with Short Hamstring Syndrome	Y	Y	Y	N	N	N	confronting two approaches
**141**	Catalan-Matamoros_2011 - A pilot study on the effect of Basic Body Awareness Therapy in patients with eating disorders: a randomized controlled trial.	Y	Y	Y	N	N	N	
**142**	**Catlin_2011 - Investigation of standard care versus sham Reiki placebo versus actual Reiki therapy to enhance comfort and well-being in a chemotherapy infusion center**	**Y**	**Y**	**Y**	**Y**	**N**	**Y**	
**143**	Cavanaugh_2016 - An acute session of roller massage prolongs voluntary	Y	N	Y	N	N	N	

	torque development and diminishes evoked pain							
**144**	Chaibi_2013 - Chiropractic spinal manipulative therapy for cervicogenic headache: A single blinded, randomized, placebo-controlled study	Y	Y	Y	Y	N	N	results presented at a congress in 2013 but no abstract available, only a protocol from 2015.
**145**	**Chaibi_2016 - Chiropractic spinal manipulative therapy for migraine: a three-armed, single-blinded, placebo, randomized controlled trial**	**Y**	**Y**	**Y**	**Y**	**N**	**Y**	
**146**	Chaibi_2015 - Chiropractic spinal manipulative therapy for cervicogenic headache: a study protocol of a single-blinded placebo-controlled randomized clinical trial	Y	Y	Y	Y	N	N	protocol
**147**	Chaibi_2015 - Chiropractic spinal manipulative therapy for migraine: a study protocol of a single-blinded placebo-controlled randomised clinical trial	Y	Y	Y	Y	N	N	protocol of ID145
**148**	Chaibi_2011 - Manual therapies for migraine: A systematic review	N	N	N	N	N	N	
**149**	**Chang_2011 - Effect of acupressure in treating urodynamic stress incontinence: A randomized controlled trial**	**Y**	**Y**	**Y**	**Y**	**N**	**Y**	
**150**	Chang_2012 - Auricular acupressure for managing postoperative pain and knee motion in patients with total knee replacement: A randomized sham control study	Y	N	Y	N	N	N	bands

151	Chang_2007 - Decrease of hypertonia after continuous passive motion treatment in individuals with spinal cord injury	Y	N	Y	N	N	N	they use a machine to do passive movement
**152**	**Chao_2013 - The beneficial effect of ST-36 (Zusanli) acupressure on postoperative gastrointestinal function in patients with colorectal cancer.**	**Y**	**Y**	**Y**	**Y**	**N**	**Y**	
153	Chatchawan_2014 - Effects of Thai traditional massage on pressure pain threshold and headache intensity in patients with chronic tension-type and migraine headaches.	Y	Y	Y	N	N	N	
**154**	**Chen_2003 - Use of acupressure to improve gastrointestinal motility in women after trans-abdominal hysterectomy**	**Y**	**Y**	**Y**	**Y**	**N**	**Y**	
**155**	**Chen_1999 - The effectiveness of acupressure in improving the quality of sleep of institutionalized residents.**	**Y**	**Y**	**Y**	**Y**	**N**	**Y**	
156	Childs_2004 - Immediate Improvements in Side-to-Side Weight Bearing and Iliac Crest Symmetry After Manipulation in Patients with Low Back Pain	N	Y	N	N	N	N	
157	Chilibeck_2011 - The effect of spinal manipulation on imbalances in leg strength	N	Y	N	N	N	N	

**158**	Chippendale_2013 - Upper cervical manual techniques and measures of autonomic nervous system control	N	N	N	N	N	N	
**159**	Chung_2014 - Acupoint stimulation to improve analgesia quality for lumbar spine surgical patients	Y	Y	Y	N	N	N	difference is the seed
**160**	Chung_2014 - Integrative acupoint stimulation to alleviate postoperative pain and morphine-related side effects: A sham-controlled study	Y	Y	Y	N	N	N	They use embedding-seed
**161**	Çıtak-Karakaya_2006 - Short and Long-Term Results of Connective Tissue Manipulation and Combined Ultrasound Therapy in Patients with Fibromyalgia.	N	Y	N	N	N	N	
**162**	**Cleland_2004 - Short-term effects of thoracic manipulation on lower trapezius muscle strength**	**Y**	**Y**	**Y**	**Y**	**N**	**Y**	
**163**	**Cleland_2005 - Immediate effects of thoracic manipulation in patients with neck pain: a randomized clinical trial**	**Y**	**Y**	**Y**	**Y**	**N**	**Y**	
**164**	Clinton_1993 - The effect of a chiropractic adjustment of the first rib on the electric skin response in ipsilateral and contralateral human forelimbs.	N	Y	N	N	N	N	
**165**	Close_2016 - A pilot randomised controlled trial (RCT) investigating the effectiveness of reflexology for	Y	N	N	N	N	N	footbaths as sham

	managing pregnancy low back and/or pelvic pain							
**166**	Cohen-Mansfield_2007 - Nonpharmacological treatment of agitation: a controlled trial of systematic individualized intervention	N	N	N	N	N	N	
**167**	Collins_2014 - The effectiveness of strain counterstrain in the treatment of patients with chronic ankle instability: A randomized clinical trial	Y	Y	Y	Y	N	N	osteopathy
**168**	Colloca_2004 - Biomechanical and neurophysiological responses to spinal manipulation in patients with lumbar radiculopathy	N	Y	N	N	N	N	using an Activator II Adjusting Instrument
**169**	Cook_2014 - The addition of cervical unilateral posterior–anterior mobilisation in the treatment of patients with shoulder impingement syndrome: A randomised clinical trial.	Y	Y	Y	N	N	N	comparison
**170**	Cowan_2002 - Physical therapy alters recruitment of the vasti in patellofemoral pain syndrome.	Y	N	N	N	N	N	
**171**	Crothers_2016 - Spinal manipulative therapy, Graston technique and placebo for non-specific thoracic spine pain: a randomised controlled trial	Y	Y	Y	N	N	N	
**172**	**Cruz-Díaz_2015 - Effects of joint mobilization on chronic ankle instability: a randomized controlled trial**	**Y**	**Y**	**Y**	**Y**	**N**	**Y**	

**173**	Cutler_2005 - Cranial manipulation can alter sleep latency and sympathetic nerve activity in humans: a pilot study	Y	Y	Y	N	N	N	
**174**	Dagfinrud_2014 - Multimodal physiotherapy may be no better than sham treatment for people with hip osteoarthritis	Y	Y	Y	N	N	N	
**175**	Daligadu_2013 - Alterations in Cortical and Cerebellar Motor Processing in Subclinical Neck Pain Patients Following Spinal Manipulation	Y	Y	Y	N	N	N	
**176**	Darsareh_2012 - Effect of aromatherapy massage on menopausal symptoms using menopausal rating scale	Y	Y	Y	N	N	N	
**177**	Davenport_2010 - Ankle manual therapy for individuals with post-acute ankle sprains: description of a randomized, placebo-controlled clinical trial	Y	Y	Y	Y	N	N	first mail sent to corresponding author on 25/10/2017, second mail sent on 15/11/2017, no answer
**178**	Dawson_2011 - Effectiveness of regular proactive massage therapy for novice recreational runners	Y	Y	Y	N	N	N	Control subjects were given no massage treatments
**179**	De Groef_2016 - Effect of myofascial techniques in addition to standard physical therapy for treatment of pain and upper limb problems in breast cancer survivors: Randomized controlled trial	Y	Y	Y	Y	Y	N	abstract presented at a conference, full texts published in 2017

**180**	de Jong_2013 - Combined arm stretch positioning and neuromuscular electrical stimulation during rehabilitation does not improve range of motion, shoulder pain or function in patients after stroke: a randomised trial	Y	N	Y	N	N	N	
**181**	de Souza Cavalcante_2016 - Effect of the Spiritist "passe" energy therapy in reducing anxiety in volunteers: A randomized controlled trial	Y	N	Y	N	N	N	No touch
**182**	Debbi_2014 - A biomechanical therapy program for patients after total knee arthroplasty - A randomized controlled trial (preliminary results)	Y	N	Y	N	N	N	device
**183**	**Delgado-Gil_2015 - Effects of mobilization with movement on pain and range of motion in patients with unilateral shoulder impingement syndrome: a randomized controlled trial.**	**Y**	**Y**	**Y**	**Y**	**N**	**Y**	
**184**	Demers_2014 - An audiometric study of the effects of paraspinal stimulation on hearing acuity in human subjects - understanding the Harvey Lillard phenomenon	Y	N	Y	N	N	N	tens
**185**	Desai_2013 - Immediate Effect of Scapular Repositioning With Active Cervical Rotation in Acute Spasmodic Torticollis	Y	Y	Y	N	N	N	The comparison group was treated with only conventional physiotherapy (microwave diathermy, submaximal isometrics, and ergonomic advice

**186**	Descarreaux_2004 - Efficacy of Preventive Spinal Manipulation for Chronic Low-Back Pain and Related Disabilities: A Preliminary Study	Y	Y	Y	N	N	N	treat Vs treat
**187**	DeVocht_2013 - A pilot study of a chiropractic intervention for management of chronic myofascial temporomandibular disorder	Y	Y	Y	N	N	N	Activator Method Chiropractic Technique
**188**	Deyle_2000 - Effectiveness of manual physical therapy and exercise in osteoarthritis of the knee. A randomized, controlled trial	Y	Y	Y	N	N	N	subtherapeutic ultrasound
**189**	Dhami_1992 - Modification of blood beta-endorphins and melatonin levels in chronic head and neck pain in male patients by spinal manipulation	Y	Y	Y	Y	Y	N	Poster congress; paper not found;
**190**	Díaz Cerrato_2009 - Modificaciones en la presión intraocular y la presión arterial en pacientes con diabetes mellitus tipo 1 tras la manipulación global occipucio-atlas-axis según Fryette. Ensayo clínico aleatorizado	Y	Y	Y	Y	N	N	osteopathy
**191**	Dimitrova_2011 - Effect of neurodynamic techniques on recovery of upper limb after olecranon fractures	Y	Y	Y	Y	N	N	poster congress
**192**	Dishman_2001 - Comparison of effects of spinal manipulation and massage on motoneuron excitability	Y	Y	Y	N	N	N	osteopathy

**193**	Dougherty_2005 - Chiropractic management of musculoskeletal pain in the multiple sclerosis patient.	Y	Y	Y	N	N	N	conceptual
**194**	Dougherty_2014 - Spinal Manipulative Therapy for Chronic Lower Back Pain in Older Veterans: A Prospective, Randomized, Placebo-Controlled Trial.	Y	Y	Y	N	N	N	detuned ultrasound
**195**	**Dunning_2009 - The effects of cervical high-velocity low-amplitude thrust manipulation on resting electromyographic activity of the biceps brachii muscle**	**Y**	**Y**	**Y**	**Y**	**Y**	**Y**	
**196**	Dwyer_2015 - Manual and Manipulative Therapy in Addition to Rehabilitation for Osteoarthritis of the Knee: Assessor-Blind Randomized Pilot Trial	Y	Y	Y	N	N	N	difference is the exercise program
**197**	Eardley_2013 - Professional kinesiology practice for chronic low back pain: single-blind, randomised controlled pilot study	Y	?	Y	?	N	N	
**198**	**Ebadi_2015 - The effect of foot reflexology on physiologic parameters and mechanical ventilation weaning time in patients undergoing open-heart surgery: A clinical trial study.**	**Y**	**Y**	**Y**	**Y**	**N**	**Y**	
**199**	Ebenbichler_2015 - Twelve-year follow-up of a randomized controlled trial of comprehensive physiotherapy following disc herniation operation.	Y	Y	Y	Y	N	N	follow-up of ID203

**200**	Efstathiou_2015 - Effectiveness of neural mobilization in patients with spinal radiculopathy: A critical review	N	N	N	N	N	N	
**201**	Ekici_2009 - Comparison of Manual Lymph Drainage Therapy and Connective Tissue Massage in Women With Fibromyalgia: A Randomized Controlled Trial.	Y	Y	Y	N	N	N	
**202**	Ellis_2005 - Efficacy of a physical therapy program in patients with Parkinson's disease: A randomized controlled trial	Y	N	Y	N	N	N	difference is the exercise program
**203**	Erdogmus_2007 - Physiotherapy-based rehabilitation following disc herniation operation: results of a randomized clinical trial	Y	N	Y	N	N	N	focused on exercises at home
**204**	**Erhardt_2015 - The immediate effect of atlanto-axial high velocity thrust techniques on blood flow in the vertebral artery: A randomized controlled trial.**	**Y**	**Y**	**Y**	**Y**	**N**	**Y**	
**205**	Espejo Antúnez_2014 - efectos sobre la tensión neural adversa medida mediante test de Slump tras punción seca de punto gatillo miofascial del músculo gastrocnemio	Y	N	Y	N	N	N	No manual therapy
**206**	Espí-López_2014 - Treatment of tension-type headache with articulatory and suboccipital soft tissue therapy: A double-blind, randomized, placebo-controlled clinical trial	Y	Y	Y	N	N	N	The control group received no treatment,

207	Evans_2012 - Do we have any control? The practitioner's influence on the ‘placebo effect’ and therapeutic outcomes within complementary and alternative medicine	N	N	N	N	N	N	
208	Fan_2015 - A randomized, placebo-controlled trial of vertebral mobilization treatment on patients with acute radiculopathy caused by lumbar disc herniation.	Y	Y	Y	N	N	N	the same of ID280
209	Farthing_2006 - The effect of different rates of application of a rib raising technique on cardiovascular and respiratory measures in asymptomatic individuals.	Y	Y	Y	Y	Y	N	Osteopathy
**210**	**Fassoulaki_2007 - Acupressure on the extra 1 acupoint: The effect on bispectral index, serum melatonin, plasma beta-endorphin, and stress**	**Y**	**Y**	**Y**	**Y**	**N**	**Y**	
211	Felhendler_1996 - Pressure on acupoints decreases postoperative pain	Y	N	Y	N	N	N	dentist' tool to make pressure
**212**	**Fernandez-de-Las-Penas_2008 - Changes in pressure pain thresholds over C5-C6 zygapophyseal joint after a cervicothoracic junction manipulation in healthy subjects**	**Y**	**Y**	**Y**	**Y**	**N**	**Y**	
213	Fernández-de-las-Peñas_2006 - The immediate effect of ischemic compression technique and transverse friction massage on tenderness of active	N	N	N	N	N	N	no control

	and latent myofascial trigger points: a pilot study.							
**214**	Fernandez-de-las-Penas_2006 - Methodological quality of randomized controlled trials of spinal manipulation and mobilization in tension-type headache, migraine, and cervicogenic headache	N	N	N	N	N	N	
**215**	Fernández-Lao_2012 - Attitudes towards massage modify effects of manual therapy in breast cancer survivors: a randomised clinical trial with crossover design.	Y	Y	Y	N	N	N	
**216**	**Ferragut-Garcias_2016 - Effectiveness of a Treatment Involving Soft Tissue Techniques and/or Neural Mobilization Techniques in the Management of Tension-Type Headache: A Randomized Controlled Trial**	**Y**	**Y**	**Y**	**Y**	**N**	**Y**	
**217**	Ferreira_2016 - Neurodynamic treatment did not improve pain and disability at two weeks in patients with chronic nerve-related leg pain: a randomised trial	Y	Y	Y	N	N	N	
**218**	Field_2016 - Massage therapy research review	N	N	N	N	N	N	
**219**	Field_2003 - Movement and massage therapy reduce fibromyalgia pain	Y	Y	Y	N	N	N	relaxation therapy

**220**	Field_2007 - Lower back pain and sleep disturbance are reduced following massage therapy	Y	Y	Y	N	N	N	relaxation therapy
**221**	Fisher_2015 - The Effect of Cervical Spine Manipulation on Postural Sway in Patients With Nonspecific Neck Pain	Y	Y	Y	Y	N	N	osteopathy
**222**	Fishman, L. M., C. Anderson, et al. (2002) BOTOX and physical therapy in the treatment of piriformis syndrome. Am J Phys Med Rehabil 936-942 DOI: 10.1097/01.PHM.0000034956.35609.5 E	Y	N	Y	N	N	N	
**223**	Fitzgerald_2001 - Considerations for planning and conducting clinic-based research in physical therapy.	N	N	N	N	N	N	
**224**	Frankel_1997 - The effect of reflexology on baroreceptor reflex sensitivity, blood pressure and sinus arrhythmia	Y	Y	Y	N	N	N	The subjects assigned to the control group received no intervention therapy
**225**	Frey_2008 - Massage Reduces Pain Perception and Hyperalgesia in Experimental Muscle Pain: A Randomized, Controlled Trial	Y	Y	Y	N	N	N	
**226**	Fryer_2004 - The effect of manipulation and mobilisation on pressure pain thresholds in the thoracic spine	Y	Y	Y	N	N	N	
**227**	Fryer_2005 - The effect of manual pressure release on myofascial trigger points in the upper trapezius muscle	Y	Y	Y	Y	N	N	osteopathy

**228**	Furlan Andrea_2015 - Massage for low-back pain. Cochrane Database of Systematic Reviews	N	N	N	N	N	N	
**229**	Galindez-Ibarbengoetxea_2016 - Randomised controlled pilot trial of high-velocity, low-amplitude manipulation on cervical and upper thoracic spine levels in asymptomatic subjects.	Y	Y	Y	Y	N	N	osteopathy
**230**	**Ganesh_2015 - Effect of cervical mobilization and ischemic compression therapy on contralateral cervical side flexion and pressure pain threshold in latent upper trapezius trigger points**	**Y**	**Y**	**Y**	**Y**	**N**	**Y**	
**231**	Garcia_2015 - Efficacy of the McKenzie method in patients with chronic nonspecific low back pain: a protocol of randomized placebo-controlled trial	Y	Y	Y	N	N	N	No manual
**232**	**Geisser_2005 - A randomized, controlled trial of manual therapy and specific adjuvant exercise for chronic low back pain**	**Y**	**Y**	**Y**	**Y**	**N**	**Y**	
**233**	Gemma_2014 - Efficacy of Manual and Manipulative Therapy in the Perception of Pain and Cervical Motion in Patients With Tension-Type Headache: A Randomized, Controlled Clinical Trial	Y	Y	Y	N	N	N	manual therapy Vs manipulative therapy Vs combination
**234**	Gemmell_2008 - Relative immediate effect of ischaemic compression and	Y	Y	Y	N	N	N	comparing techniques

	activator trigger point therapy on active upper trapezius trigger points: A randomised trial							
**235**	Gemmell_2008 - Immediate effect of ischaemic compression and trigger point pressure release on neck pain and upper trapezius trigger points: A randomised controlled trial	Y	Y	Y	N	N	N	TENS
**236**	Gemmell_1992 - Treatment of chronic low back pain with low force manipulation	Y	Y	Y	Y	N	N	first mail to corresponding author on 02.11.2017, second mail sent on the 24.11.2017, no answer
**237**	Genç_2013 - The efficiency of the acupressure in prevention of the chemotherapy-induced nausea and vomiting	Y	N	Y	N	N	N	wristband
**238**	Gentil-Bécoz_2015 - The immediate effects of two muscle energy techniques on quadriceps muscle during vertical jumps: A pilot Study	Y	Y	Y	N	N	N	They used classic massage as sham, not a proper sham
**239**	George_2006 - The effects of active release technique on carpal tunnel patients: A pilot study	N	Y	N	N	N	N	pilot study (no control)
**240**	George_2008 - Effects of Manual Therapy on Pain Perception in Individuals with Carpal Tunnel Syndrome	Y	Y	Y	Y	N	N	requested via Reserach Gate on 01.11.2017 and 22.11.2017 , no answer
**241**	Gerard_2003 - A randomized controlled trial of spiritual healing in restricted neck movement	Y	N	Y	N	N	N	

**242**	**Ghroubi_2007 - Les lombalgiques chroniques et manipulations vertébrales. Étude prospective à propos de 64 cas**	**Y**	**Y**	**Y**	**Y**	**Y**	**Y**	
**243**	Gieron_1993 **-** Acupressure for the prophylaxis of postoperative nausea and vomiting	Y	N	Y	N	N	N	not found
**244**	**Goertz_2016 - Effect of Spinal Manipulation of Upper Cervical Vertebrae on Blood Pressure: Results of a Pilot Sham-Controlled Trial**	**Y**	**Y**	**Y**	**Y**	**N**	**Y**	
**245**	Gomez Conesa_2011 **-** Effectiveness of suboccipital soft tissue therapy in the treatment of tension-type headache	Y	Y	Y	N	N	N	placebo is "resting postion"
**246**	Gonzalez-Alvarez_2015 **-** Effects of a diaphragm stretching technique on pulmonary function in healthy participants: A randomized-controlled trial	Y	Y	Y	N	N	N	osteopathy
**247**	González-Álvarez_2016 **-** effects of diaphragm stretching on posterior chain muscle kinematics and rib cage and abdominal excursion: A randomized controlled trial.	Y	Y	Y	N	N	N	sham-ultrasound
**248**	González-Iglesias_2009 **-** Inclusion of thoracic spine thrust manipulation into an electro-therapy/thermal program for the management of patients with acute mechanical neck pain: A randomized clinical trial	Y	Y	Y	N	N	N	excluded because they just add SMT at a therapy

**249**	Goodsell_2000 - Short-term effects of lumbar posteroanterior mobilization in individuals with low-back pain	Y	Y	Y	N	N	N	each patient lying on the treatment couch in a prone position for 3 minutes
**250**	Goodwin_2007 - Effect of pre-performance lower-limb massage on thirty-meter sprint running	Y	Y	Y	N	N	N	placebo ultrasound
**251**	Gordon_1998 - The effects of therapeutic touch on patients with osteoarthritis of the knee.	Y	Y	Y	Y	Y	N	the email address is inactive, excluded
**252**	**Gozuyesil_2016 - The effect of foot reflexology applied to women aged between 40 and 60 on vasomotor complaints and quality of life**	**Y**	**Y**	**Y**	**Y**	**N**	**Y**	
**253**	**Gregson_2015 - Acupressure for inducing labour for nulliparous women with post-dates pregnancy**	**Y**	**Y**	**Y**	**Y**	**N**	**Y**	
**254**	Grieve_2011 - The immediate effect of soleus trigger point pressure release on restricted ankle joint dorsiflexion: A pilot randomised controlled trial	Y	Y	Y	N	N	N	no therapy
**255**	Grieve_2013 - The immediate effect of triceps surae myofascial trigger point therapy on restricted active ankle joint dorsiflexion in recreational runners: A crossover randomised controlled trial	Y	Y	Y	N	N	N	no therapy
**256**	Grieve_2015 - The immediate effect of bilateral self myofascial release on the plantar surface of the feet on hamstring and lumbar spine flexibility: A pilot randomised controlled trial	Y	Y	Y	N	N	N	no therapy

**257**	Grindstaff_2009 - Effects of lumbopelvic joint manipulation on quadriceps activation and strength in healthy individuals	Y	Y	Y	Y	N	N	no manual placebo
**258**	Groisman_2014 - H-reflex responses to High-Velocity Low-Amplitude manipulation in asymptomatic adults	Y	Y	Y	Y	Y	N	osteopathy
**259**	Gross_2015 - Manipulation and mobilisation for neck pain contrasted against an inactive control or another active treatment.	N	N	N	N	N	N	
**260**	**Guimarães_2016 - Immediate Effects of Mobilization With Movement vs Sham Technique on Range of Motion, Strength, and Function in Patients With Shoulder Impingement Syndrome: Randomized Clinical Trial**	**Y**	**Y**	**Y**	**Y**	**N**	**Y**	
**261**	Gulick_2011 - Effect of ischemic pressure using a Backnobber II device on discomfort associated with myofascial trigger points	Y	Y	Y	N	N	N	device
**262**	Gupta_2015 - Does therapist-assisted exercise improve pregnancy related pelvic girdle pain? A randomised, cross-over, blinded, sham-controlled trial	Y	Y	Y	N	N	N	paper not found
**263**	Gyulai_2015 - BEMER therapy combined with physiotherapy in patients with musculoskeletal diseases:	Y	N	Y	N	N	N	

	A randomised, controlled double blind follow-up pilot study							
**264**	Haas_2004 - Dose-response for chiropractic care of chronic low back pain	Y	Y	Y	Y	N	N	no placebo: the outcome is the observe the dose-response
**265**	Haas_2010 - Dose response and efficacy of spinal manipulation for chronic cervicogenic headache: a pilot randomized controlled trial	Y	Y	Y	Y	N	N	they use a light massage as control intervention but also in the hvla there is a preintervention of light massage
**266**	Haas_2014 - Dose-response and efficacy of spinal manipulation for care of chronic low back pain: a randomized controlled trial	Y	Y	Y	Y	N	N	they use light massage, but THEY SAY "it was not a formal sham"
**267**	Haavik_2011 - Subclinical Neck Pain and the Effects of Cervical Manipulation on Elbow Joint Position Sense	Y	Y	Y	N	N	N	control: 5-minute rest period
**268**	**Haavik_2016 - Effect of Spinal Manipulation on Pelvic Floor Functional Changes in Pregnant and Nonpregnant Women: A Preliminary Study**	**Y**	**Y**	**Y**	**Y**	**N**	**Y**	
**269**	**Haavik-Taylor_2007 - Cervical spine manipulation alters sensorimotor integration: A somatosensory evoked potential study**	**Y**	**Y**	**Y**	**Y**	**N**	**Y**	
**270**	**Habek_2004 - Success of acupuncture and acupressure of the Pc 6 acupoint in the treatment of hyperemesis gravidarum**	**Y**	**Y**	**Y**	**N**	**N**	**Y**	

271	Hage_2016 - Influence of an upper cervical thrust on the motor control of the neck: A single blind randomized trial	Y	Y	Y	Y	N	N	the author said that the paper is not yet available
272	**Haik_2014 - Scapular kinematics pre- and post-thoracic thrust manipulation in individuals with and without shoulder impingement symptoms: a randomized controlled study**	**Y**	**Y**	**Y**	**Y**	**N**	**Y**	
273	**Hains_2010 - Chronic Shoulder Pain of Myofascial Origin: A Randomized Clinical Trial Using Ischemic Compression Therapy**	**Y**	**Y**	**Y**	**Y**	**N**	**Y**	
274	Hains_2000 - A combined ischemic compression and spinal manipulation in the treatment of fibromyalgia: A preliminary estimate of dose and efficacy	N	Y	N	N	N	N	no control
275	**Hains_2010 - Patellofemoral pain syndrome managed by ischemic compression to the trigger points located in the peri-patellar and retro-patellar areas: A randomized clinical trial**	**Y**	**Y**	**Y**	**Y**	**N**	**Y**	
276	Hall_2016 - The effectiveness of complementary manual therapies for pregnancy-related back and pelvic pain: A systematic review with meta-analysis	N	N	N	N	N	N	
277	**Hall_2005 - Mulligan bent leg raise technique--a preliminary randomized**	**Y**	**Y**	**Y**	**Y**	**N**	**Y**	

	**trial of immediate effects after a single intervention**							
**278**	Hamilton_2007 - The effects of high-velocity, low-amplitude manipulation and muscle energy technique on suboccipital tenderness	Y	Y	Y	Y	N	N	osteopathy
**279**	Hammes_2010 - Acupuntura y acupresión auricular para el alivio de los sofocos de la menopausia: ensayo aleatorizado controlado en mujeres chinas sometidas a ovariectomía bilateral	Y	Y	Y	N	N	N	hormone replacement therapy (comparison)
**280**	Han_2015 - Short-term study on risk-benefit outcomes of two spinal manipulative therapies in the treatment of acute radiculopathy caused by lumbar disc herniation: study protocol for a randomized controlled trial	Y	Y	Y	Y	N	N	PROTOCOL
**281**	Han_2003 - Effectiveness of aromatherapy massage on abdominal obesity among middle aged women	Y	Y	Y	Y	Y	N	corean. first mail sent on 12.11.2017 to corresponding author, second mail sent on 2.12.2017. no answer
**282**	Hancock_2008 - Independent evaluation of a clinical prediction rule for spinal manipulative therapy: A randomised controlled trial	Y	Y	Y	N	N	N	The placebo therapy used was detuned pulsed ultrasound
**283**	Hansen_1993 - Intensive, dynamic back-muscle exercises, conventional physiotherapy, or placebo-control treatment of low-back pain. A randomized, observer-blind trial	Y	N	Y	N	N	N	placebo-control treatment involving semihot packs and light traction

**284**	Hansen_2006 - Does aromatherapy massage reduce job-related stress? Results from a randomised, controlled trial	Y	Y	Y	N	N	N	
**285**	Harmon_1999 - Acupressure and the prevention of nausea and vomiting after laparoscopy	Y	N	Y	N	N	N	wristbands
**286**	Hart_2015 - Short-Term Stability of Resting Pulse Rates in Chiropractic Students	Y	Y	Y	Y	N	N	no intervention
**287**	Hashemi_2016 - Studying the Effectiveness of One Type of Iranian Traditional Massage on Lumbar Radiculopathy	Y	Y	Y	N	N	N	They used traditional massage as control so this is a comparison
**288**	**Hawk_1999 - Preliminary study of the effects of a placebo chiropractic treatment with sham adjustments**	**Y**	**Y**	**Y**	**Y**	**N**	**Y**	
**289**	**hawk_2002 - Issues in Planning a Placebo-Controlled Trial of**	**Y**	**Y**	**Y**	**Y**	**N**	**Y**	
**290**	**Hawk_2005 - A randomized trial investigating a chiropractic manual placebo: a novel design using standardized forces in the delivery of active and control treatments**	**Y**	**Y**	**Y**	**Y**	**N**	**Y**	
**291**	He_2013 - Auricular Acupressure for Analgesia in Perioperative Period of Total Knee Arthroplasty	Y	Y	Y	N	N	N	vaccaria seeds
**292**	He_2015 - Effect of Combined Manual Acupuncture and Massage on Body	Y	Y	Y	N	N	N	combination of treatment

	Weight and Body Mass Index Reduction in Obese and Overweight Women: A Randomized, Short-term Clinical Trial.							
293	Heazell_2006 - Acupressure for the in-patient treatment of nausea and vomiting in early pregnancy: a randomized control trial	Y	N	Y	N	N	N	
**294**	**Hedlund_2014 - Effect of chiropractic manipulation on vertical jump height in young female athletes with talocrural joint dysfunction: a single-blind randomized clinical pilot trial**	**Y**	**Y**	**Y**	**Y**	**N**	**Y**	
295	Hemmilä_2005 - Bone Setting for Prolonged Neck Pain: A Randomized Clinical Trial	Y	Y	Y	N	N	N	control: follow-up without therapy
296	Hemmilä_1997 - Does folk medicine work? A randomized clinical trial on patients with prolonged back pain	Y	Y	Y	N	N	N	no manual sham
297	Henschke_2012 - Trends over time in the size and quality of randomised controlled Trials of interventions for chronic low-back pain	N	N	N	N	N	N	
**298**	**Her_2010 - Intraocular pressure-lowering effect of auricular acupressure in patients with glaucoma: A prospective, single-blinded, randomized controlled trial**	**Y**	**Y**	**Y**	**Y**	**N**	**Y**	
**299**	**Heredia Rizo_2012 - Immediate effects of the suboccipital muscle inhibition technique in craniocervical**	**Y**	**Y**	**Y**	**Y**	**N**	**Y**	

	**posture and greater occipital nerve mechanosensitivity in subjects with a history of orthodontia use: a randomized trial**							
**300**	**Heredia-Rizo_2013 - Immediate Changes in Masticatory Mechanosensitivity, Mouth Opening, and Head Posture After Myofascial Techniques in Pain-Free Healthy Participants: A Randomized Controlled Trial**	**Y**	**Y**	**Y**	**Y**	**N**	**Y**	
**301**	Hernandez_1999 - Multiple sclerosis patients benefit from massage therapy	Y	Y	Y	N	N	N	The control group received standard medical care
**302**	Hernandez-Reif_1998 - Multiple sclerosis patients benefit from massage therapy	Y	Y	Y	N	N	N	no manual control
**303**	**Heymann_2013 - Spinal high-velocity low amplitude manipulation in acute nonspecific low back pain: a double-blinded randomized controlled trial in comparison with diclofenac and placebo**	**Y**	**Y**	**Y**	**Y**	**N**	**Y**	
**304**	Hidalgo_2015 - Étude sur l'efficacité des techniques Mulligan auprès de patients présentant une lombalgie non spécifique : étude clinique placebo-controlée et randomisée	Y	Y	Y	Y	N	N	Poster presented at a congress - first mail to corresponding author on 14.11.2017, second mail on 2.12.2017, no answer
**305**	**Hidalgo_2015 - Short-term effects of Mulligan mobilization with movement on pain, disability, and kinematic spinal movements in patients with nonspecific low back**	**Y**	**Y**	**Y**	**Y**	**N**	**Y**	

	**pain: a randomized placebo-controlled trial**							
**306**	Hilbert_2003 - The effects of massage on delayed onset muscle soreness.	Y	Y	Y	N	N	N	no manual control
**307**	**Ho_2010 - Impact of massage therapy on motor outcomes in very low-birthweight infants: Randomized controlled pilot study**	**Y**	**Y**	**Y**	**Y**	**N**	**Y**	
**308**	Hofmann_2013 - Acupressure in Management of Postoperative Nausea & Vomiting (PONV) in High Risk Ambulatory Surgical Patients	Y	N	Y	N	N	N	
**309**	**hoiriis_2004 - a randomized clinical trial comparing**	**Y**	**N**	**Y**	**Y**	**N**	**Y**	
**310**	**Holt_2009 - The effectiveness of foot reflexology in inducing ovulation: a sham-controlled randomized trial**	**Y**	**Y**	**Y**	**Y**	**N**	**Y**	
**311**	Holt_2016 - Effectiveness of Chiropractic Care to Improve Sensorimotor Function Associated With Falls Risk in Older People: A Randomized Controlled Trial	Y	Y	Y	N	N	N	compared with no intervention
**312**	**Hondras_1999 - Spinal manipulative therapy versus a low force mimic maneuver for women with primary dysmenorrhea: a randomized, observer-blinded, clinical trial.**	**Y**	**Y**	**Y**	**Y**	**N**	**Y**	
**313**	hong_1993 - Immediate Effects of Various Physical Medicine	Y	Y	Y	N	N	N	

**314**	**Hosseinabadi_2015 - The Effect of Acupressure on Pain and Anxiety Caused by Venipuncture**	**Y**	**Y**	**Y**	**Y**	**N**	**Y**	
**315**	Howatson_2005 - The efficacy of ice massage in the treatment of exercise-induced muscle damage	Y	Y	Y	N	N	N	An ultrasound machine
**316**	Hsiung_2015 - Acupressure improves the postoperative comfort of gastric cancer patients: A randomised controlled trial	Y	Y	Y	N	N	N	no acupressure
**317**	Hu_1995 - P6 acupressure reduces symptoms of vection-induced motion sickness	Y	Y	Y	Y	Y	N	paper not found, only abstract on PubMed and RG. E-mail not found.
**318**	**Hughes_2009 - Reflexology for the treatment of pain in people with multiple sclerosis: a double-blind randomised sham-controlled clinical trial**	**Y**	**Y**	**Y**	**Y**	**N**	**Y**	
**319**	**Humphries_2013 - Immediate effects of lower cervical spine manipulation on handgrip strength and free-throw accuracy of asymptomatic basketball players: A pilot study.**	**Y**	**Y**	**Y**	**Y**	**N**	**Y**	
**320**	Hur_2007 - Effects of aromatherapy massage on blood pressure and lipid profile in Korean climacteric women	Y	Y	Y	N	N	N	
**321**	Ibanez Garcia_2008 - Effects of a protocol of two manual techniques on latent myofascial trigger points of the masseter muscle	Y	Y	Y	Y	N	N	osteopathy

**322**	Ibánez_2009 - Changes in masseter muscle trigger points following strain-counterstrain or neuro-muscular technique	Y	Y	Y	Y	N	N	osteopathy
**323**	Iqbal_2012 - Whether does acupressure (P6) prevent nausea and vomiting in patients undergoing laparoscopic surgery	Y	Y	Y	Y	Y	N	Full Text requested to all authors through Research Gate on 26.11.2017, it wasn't possible to make a second request. No answer.
**324**	**Ireland_1998 - Therapeutic touch with HIV-infected children: a pilot study.**	**Y**	**Y**	**Y**	**Y**	**N**	**Y**	
**325**	Jane_2011 - Effects of massage on pain, mood status, relaxation, and sleep in Taiwanese patients with metastatic bone pain: A randomized clinical trial	Y	Y	Y	N	N	N	
**326**	Jayson_1981 - Mobilization and manipulation for low-back pain	N	Y	N	N	N	N	
**327**	Johnson_1999 - A controlled investigation of bodywork in multiple sclerosis	Y	Y	Y	Y	N	N	Feldenkrais Method
**328**	Jones_2013 - Is there a specific hemodynamic effect in reflexology? A systematic review of randomized controlled trials	N	N	N	N	N	N	
**329**	Jones_2012 - Reflexology has an acute (immediate) haemodynamic effect in healthy volunteers: A double-blind randomised controlled trial	Y	Y	Y	Y	N	N	same as ID330

**330**	**Jones_2013 - Reflexology has no immediate haemodynamic effect in patients with chronic heart failure: a double blind randomised controlled trial**	**Y**	**Y**	**Y**	**Y**	**N**	**Y**	
**331**	**jowsey_2010 - Sympathetic nervous system effects in the hands following a grade III**	**Y**	**Y**	**Y**	**Y**	**N**	**Y**	
**332**	Ju_2013 **-** Effects of aroma massage on home blood pressure, ambulatory blood pressure, and sleep quality in middle-aged women with hypertension	Y	Y	Y	N	N	N	massage using artificial fragrance oil once a week and body cream once a day.
**333**	**Jun_2007 - Effects of acupressure on dysmenorrhea and skin temperature changes in college students: A non-randomized controlled trial**	**Y**	**Y**	**Y**	**Y**	**N**	**Y**	
**334**	**Kamel_2016 - Efficacy of lumbar mobilization on postpartum low back pain in Egyptian females: A randomized control trial**	**Y**	**Y**	**Y**	**Y**	**N**	**Y**	
**335**	**Kanitz_2015 - A randomised, controlled, single-blinded study on the impact of a single rhythmical massage (anthroposophic medicine) on well-being and salivary cortisol in healthy adults**	**Y**	**Y**	**Y**	**Y**	**N**	**Y**	
**336**	Kannus_1999 **-** An outcome study of chronic patellofemoral pain syndrome. Seven-year follow-up of patients in a randomized, controlled trial	Y	N	Y	N	N	N	

**337**	Kappetijn_2014 - Efficacy of passive extension mobilization in addition to exercise in the osteoarthritic knee: An observational parallel-group study	Y	Y	Y	N	N	N	
**338**	Karason_2003 - Somatovisceral response following osteopathic HVLAT: a pilot study on the effect of unilateral lumbosacral high-velocity low-amplitude thrust technique on the cutaneous blood flow in the lower limb	Y	Y	Y	Y	N	N	osteopathy
**339**	**Kardouni_2015 - Thoracic spine manipulation in individuals with subacromial impingement syndrome does not immediately alter thoracic spine kinematics, thoracic excursion, or scapular kinematics: A randomized controlled trial**	**Y**	**Y**	**Y**	**Y**	**N**	**Y**	
**340**	**Kardouni_2015 - Immediate changes in pressure pain sensitivity after thoracic spinal manipulative therapy in patients with subacromial impingement syndrome: A randomized controlled study.**	**Y**	**Y**	**Y**	**Y**	**N**	**Y**	
**341**	Karpouzis_2011 - Final data of the effects of the Neuro Emotional Technique (NET) for pediatric Attention-Deficit/Hyperactivity Disorder (AD/HD): A randomized controlled trial	Y	Y	Y	Y	N	N	same as ID342
**342**	Karpouzis_2009 - A randomised controlled trial of the Neuro Emotional Technique (NET) for childhood	Y	Y	Y	Y	N	N	NET

	Attention Deficit Hyperactivity Disorder (ADHD): a protocol							
**343**	Kashefi_2010 - Effect of acupressure at the Sanjiao point (SP6) on women's general heath with dysmenorrhea: A randomized controlled trial	Y	Y	Y	Y	Y	N	same as ID344
**344**	**Kashefi_2011 - The efficacy of acupressure at the sanyinjiao point in the improvement of women's general health**	**Y**	**Y**	**Y**	**Y**	**N**	**Y**	
**345**	**Kawchuk_2009 - A True Blind for Subjects Who Receive Spinal Manipulation Therapy**	**Y**	**Y**	**Y**	**Y**	**N**	**Y**	
**346**	**Keller_2000 - Mechanical force spinal manipulation increases trunk muscle strength assessed by electromyography: a comparative clinical trial**	**Y**	**Y**	**Y**	**Y**	**N**	**Y**	
**347**	Kelly_2000 - Use of a mental rotation reaction-time paradigm to measure the effects of upper cervical adjustments on cortical processing: A pilot study	Y	Y	Y	N	N	N	A non-intervention group was used to control for improvement
**348**	Keus_2007 - Effectiveness of physiotherapy in Parkinson's disease: The feasibility of a randomised controlled trial	Y	N	Y	N	N	N	
**349**	Khorsand_2015 - Evaluation of the Effect of Reflexology on Pain Control and Analgesic Consumption After Appendectomy	Y	N	Y	N	N	N	*"For foot reflexology, a special device called a “stick,”*

**350**	Kiernan_2010 - Effects of a Manual Medicine Treatment Procedures on Nitric Oxide Release in 23 Healthy Adult	N	Y	N	N	N	N	
**351**	Kim_2005 - Effect of aromatherapy massage for the relief of constipation in the elderly	N	Y	N	N	N	N	oil
**352**	Kim_2016 - Effect of mulligan's mobilization with movement technique on gait function in stroke patients	Y	Y	Y	N	N	N	lunges as control
**353**	Kim_2011 - Self-aromatherapy massage of the abdomen for the reduction of menstrual pain and anxiety during menstruation in nurses: A placebo-controlled clinical trial	Y	Y	Y	N	N	N	
**354**	kimber_2008 - Massage or music for pain relief in labour: A pilot randomised	Y	Y	Y	N	N	N	placebo is music with relaxation techniques
**355**	Kitay_2009 - Efficacy of combined local mechanical vibrations, continuous passive motion and thermotherapy in the management of osteoarthritis of the knee.	Y	Y	Y	N	N	N	
**356**	Klein_2013 - Strain-counterstrain to treat restrictions of the mobility of the cervical spine in patients with neck pain: a sham-controlled randomized trial.	Y	Y	Y	Y	N	N	osteopathy
**357**	Kochar_2002 - Effectiveness of a Specific Physiotherapy Regimen on	Y	Y	Y	N	Y	N	

	Patients with Tennis Elbow: Clinical study						
**358**	**Kojidi_2016 - Comparison Between the Effects of Passive and Active Soft Tissue Therapies on Latent Trigger Points of Upper Trapezius Muscle in Women: Single-Blind, Randomized Clinical Trial**	**Y**	**Y**	**Y**	**Y**	**N**	**Y**
**359**	**Kokjohn_1992 - The effect of spinal manipulation on pain and prostaglandin levels in women with primary dysmenorrhea.**	**Y**	**Y**	**Y**	**Y**	**N**	**Y**
**360**	Konstantinou_2007 **-** Flexion mobilizations with movement techniques: the immediate effects on range of movement and pain in subjects with low back pain.	Y	Y	Y	N	N	N
**361**	**Krekoukias_2009 - Comparison of surface electromyographic activity of erector spinae before and after the application of central posteroanterior mobilisation on the lumbar spine.**	**Y**	**Y**	**Y**	**Y**	**N**	**Y**
**362**	Krouwel_2010 **-** An investigation into the potential hypoalgesic effects of different amplitudes of PA mobilisations on the lumbar spine as measured by pressure pain thresholds (PPT)	Y	Y	Y	N	N	N
**363**	Kukurin_2002 **-** Chronic pediatric asthma and chiropractic spinal manipulation: A prospective clinical series and randomized clinical pilot study	N	N	N	N	N	N

**364**	Kumar_2011 - Efficacy of impairment-based manual physical therapy intervention for painful stiff shoulder in type-ii diabetes mellitus subjects-a randomized clinical trial.	Y	Y	Y	N	N	N	
**365**	Kurebayashi_2016 - Massage and Reiki used to reduce stress and anxiety: Randomized Clinical Trial	Y	Y	Y	N	N	N	Control group without intervention
**366**	Kwan_2015 - Acupressure for agitation in nursing home residents with dementia: Study protocol for a randomized controlled trial	Y	Y	Y	Y	N	N	PROTOCOL
**367**	**La Touche_2006 - Efecto pos-tratamiento de la reflexoterapia podal en la tensión arterial y la frecuencia cardiaca. Estudio piloto**	**Y**	**Y**	**Y**	**Y**	**N**	**Y**	
**368**	**Laframboise_2016 - Effect of two consecutive spinal manipulations in a single session on myofascial pain pressure sensitivity: a randomized controlled trial**	**Y**	**Y**	**Y**	**Y**	**N**	**Y**	
**369**	Lalanne_2009 - Modulation of the Flexion-Relaxation Response by Spinal Manipulative Therapy: A Control Group Study	Y	Y	Y	N	N	N	no manual control
**370**	Lämås_2016 - Does touch massage facilitate recovery after stroke? A study protocol of a randomized controlled trial.	Y	Y	Y	N	N	N	

**371**	Lascurain-Aguirrebena_2016 - Mechanism of action of spinal mobilizations a systematic review	N	N	N	N	N	N	
**372**	Le Blanc-Louvry_2002 - Does mechanical massage of the abdominal wall after colectomy reduce postoperative pain and shorten the duration of ileus? Results of a randomized study.	Y	N	Y	N	N	N	Use mechanical touch and no humans contact
**373**	**Learman_2009 - Effects of spinal manipulation on trunk proprioception in subjects with chronic low back pain during symptom remission**	**Y**	**Y**		**Y**	**N**	**Y**	
**374**	Lee_2003 - Effects of Qi-therapy on blood pressure, pain and psychological symptoms in the elderly: a randomized controlled pilot trial	Y	Y	Y	Y	N	N	
**375**	Lee_2005 - Effects of Qi-therapy (external Qigong) on cardiac autonomic tone: a randomized placebo controlled study	Y	Y	Y	Y	N	N	
**376**	Lee_2009 - Immediate effects of musculoskeletal physiotherapy and massage on pain and ease of breathing in adults with cystic fibrosis	N	Y	N	N	N	N	
**377**	Lee_2016 - A pragmatic randomised controlled trial of healing therapy in a gastroenterology outpatient setting	Y	Y	Y	Y	N	N	

**378**	Lehman_2001 - Spinal manipulation causes variable spine kinematic and trunk muscle electromyographic responses	N	Y	N	N	N	N	
**379**	Lena_2012 - Improvements of well-being after one single rhythmical massage intervention in stressed adults-a prospective, randomised, three-armed study	Y	Y	Y	Y	N	N	Oral Presentation, full text requested to the author through a private message on Research Gate (on 14.11.2017 and again on 2.12.2017), no answer
**380**	Lewis_2010 - A randomised controlled study examining the short-term effects of Strain-Counterstrain treatment on quantitative sensory measures at digitally tender points in the low back	Y	Y	Y	Y	N	N	osteopathy
**381**	Li_2002 - Use of qigong therapy in the detoxification of heroin addicts	Y	Y	Y	N	N	N	
**382**	Lim_2012 - Connective tissue reflex massage to improve peripheral circulation in type 2 diabetes mellitus	Y	Y	Y	N	N	N	
**383**	**Lin_2016 - Effectiveness of Acupressure on the Taichong Acupoint in Lowering Blood Pressure in Patients with Hypertension: A Randomized Clinical Trial**	**Y**	**Y**	**Y**	**Y**	**N**	**Y**	
**384**	Liu_2010 - Auricular acupressure as a treament for neuropathIc pain in patients with spinal cord injury	Y	Y	Y	Y	N	N	Poster Abstract - Full Text requested through Research gate on 14.11.2017 and again on 2.12.2017, no answer

**385**	López de Celis_2007 - Efectividad de la movilización posteroanterior del raquis lumbar, con la cuña de Kaltenborn, en pacientes con dolor lumbar crónico	Y	Y	Y	N	N	N	
**386**	**López-Sendín_2012 - Effects of physical therapy on pain and mood in patients with terminal cancer: A pilot randomized clinical trial**	**Y**	**Y**	**Y**	**Y**	**N**	**Y**	
**387**	Lougee_2013 - The suitability of sham treatments for use as placebo controls in trials of spinal manipulative therapy: a pilot study	N	N	N	N	N	N	osteopathy
**388**	Lu_2000 - Acupuncture/acupressure to treat gagging dental patients: a clinical study of anti-gagging effects	Y	Y	Y	Y	Y	N	Full text requested via Research Gate on 18.11.2017 and on 2.12.2017, no answer
**389**	Lubbe_2015 - Manipulative Therapy and Rehabilitation for Recurrent Ankle Sprain With Functional Instability: A Short-Term, Assessor-Blind, Parallel-Group Randomized Trial.	Y	Y	Y	N	N	N	
**390**	Lucini_2009 - Complementary medicine for the management of chronic stress: superiority of active versus passive techniques	Y	Y	Y	N	N	N	
**391**	Maa_1997 - Acupressure as an adjunct to a pulmonary rehabilitation program	Y	Y	Y	Y	Y	N	paper not found, only abstract
**392**	Macznik_2013 - Management of pain associated with acute sports injuries—Is acupressure a way to go?	Y	Y	Y	Y	N	N	Conference Abstract - Full Text requested via Research Gate on 14.11.2017 and 2.12.2017, no answer

**393**	Maddocks_2016 - Problematic placebos in physical therapy trials	N	N	N	N	N	N	
**394**	Maduro de Camargo_2011 - Immediate Effects on Electromyographic Activity and Pressure Pain Thresholds After a Cervical Manipulation in Mechanical Neck Pain: A Randomized Controlled Trial	Y	Y	Y	N	N	N	
**395**	Mafetoni_2016 - The effects of acupressure on labor pains during child birth: randomized clinical trial	Y	Y	Y	Y	N	N	same as ID396
**396**	**Mafetoni_2015 - Effects of acupressure on progress of labor and cesarean section rate: Randomized clinical trial**	**Y**	**Y**	**Y**	**Y**	**N**	**Y**	
**397**	Mancinelli_2006 - The effects of massage on delayed onset muscle soreness and physical performance in female collegiate athletes	Y	Y	Y	N	N	N	There is no sham, only time control
**398**	Manso_2010 - The effect on the pain threshold in a myotome after a spinal manipulation... is it influenced by the subject's expectations?	Y	Y	N	N	N	N	They are not testing Manual intervention but verbal suggestion
**399**	Mansour_1999 - A study to test the effectiveness of placebo Reiki standardization procedures developed for a planned Reiki efficacy study	N	N	N	N	N	N	
**400**	The Authors declared RC with Placebo, but this is a trial to investigate the effects of bladder lift manipulation on	Y	Y	Y	N	N	N	

	the pain threshold of the tibialis anterior trigger point (TATP) and fifth lumbar spinous process (5LSP) compared to a pompage of interdigital point of the right hand							
**401**	Marr_2011 - The effects of the Bowen technique on hamstring flexibility over time: A randomised controlled trial	Y	Y	Y	N	N	N	
**402**	**Marrón-Gómez_2014 - The effect of two mobilization techniques on dorsiflexion in people with chronic ankle instability**	**Y**	**Y**	**Y**	**Y**	**N**	**Y**	
**403**	Martínez-Segura_2006 - Immediate Effects on Neck Pain and Active Range of Motion After a Single Cervical High-Velocity Low-Amplitude Manipulation in Subjects Presenting with Mechanical Neck Pain: A Randomized Controlled Trial	Y	Y	Y	Y	N	N	osteopathy
**404**	Marti-Salvador_2016 - Effects of manual therapy diaphragmatic protocol in the treatment of nonspecific chronic low back pain Randomized clinical trial	Y	Y	Y	Y	N	N	osteopathy
**405**	Marzouk_2013 - The effect of aromatherapy abdominal massage on alleviating menstrual pain in nursing students: A prospective randomized cross-over study	Y	Y	Y	N	N	N	
**406**	McCarthy_2011 - The twitch response in spinal muscles with spinal manipulation: Is it clinically relevant?	Y	Y	Y	Y	N	N	

**407**	McChesney_2011 - The effect of thoracic spine high-velocity low-amplitude thrust manipulation on myoelectric activity of the lower trapezius and posterior deltoid muscles during treadmill walking	Y	Y	Y	N	N	N
**408**	**McClatchie_2009 - Mobilizations of the asymptomatic cervical spine can reduce signs of shoulder dysfunction in adults**	**Y**	**Y**	**Y**	**Y**	**N**	**Y**
**409**	**McFadden_2011 - Acupressure's efficacy as a non-pharmacological intervention for traumatic brain injury (TBI)**	**Y**	**Y**	**Y**	**Y**	**N**	**Y**
**410**	**mcfadden_2012 - Efficacy of acupressure for non-pharmacological**	**Y**	**Y**	**Y**	**Y**	**N**	**Y**
**411**	**McFadden_2010 - Cardiovascular benefits of acupressure (Jin Shin) following stroke**	**Y**	**Y**	**Y**	**Y**	**N**	**Y**
**412**	**McGuiness_1997 - Influence of a cervical mobilization technique on respiratory and cardiovascular function**	**Y**	**Y**	**Y**	**Y**	**N**	**Y**
**413**	Mehdikhani_2012 - Immediate effect of muscle energy technique on latent trigger point of upper trapezius muscle	Y	Y	Y	N	N	N
**414**	Menke_2014 - Manual therapy researchers are misled by natural history and placebo effects	N	N	N	N	N	N

**415**	Meseguer_2006 **-** Immediate effects of the strain/counterstrain technique in local pain evoked by tender points in the upper trapezius muscle	Y	Y	Y	Y	N	N	osteopathy
**416**	**Michener_2013 - Development of a sham comparator for thoracic spinal manipulative therapy for use with shoulder disorders**	**Y**	**Y**	**Y**	**Y**	**N**	**Y**	
**417**	**Michener_2015 - Validation of a sham comparator for thoracic spinal manipulation in patients with shoulder pain**	**Y**	**Y**	**Y**	**Y**	**N**	**Y**	
**418**	Midilli_2015 **-** Effects of Reiki on Post-cesarean Delivery Pain, Anxiety, and Hemodynamic Parameters: A Randomized, Controlled Clinical Trial	Y	Y	Y	N	N	N	
**419**	Milanese_2011 **-** The use of RCT's in manual therapy – Are we trying to fit a round peg into a square hole?	N	N	N	N	N	N	
**420**	miller_2011 **-** Reduced crying time for infants with colic presenting for chiropractic treatment	Y	Y	Y	Y	Y	N	paper not found, full text requested on Research gate on 01.11.2017 and 22.11.2017, no answer
**421**	miller_2012 **-** efficacy of chiropractic manual therapy on infnat	Y	Y	Y	N	N	N	
**422**	**Miller_2013 - Evaluation of the effects of reflexology on quality of life and symptomatic relief in multiple sclerosis patients with moderate to severe disability; a pilot study**	**Y**	**Y**	**Y**	**Y**	**N**	**Y**	

**423**	Mirbagher-Ajorpaz_2011 - The effects of acupressure on primary dysmenorrhea: A randomized controlled trial	Y	Y	Y	N	N	N	is a comparison between 2 treatment without placebo
**424**	Mohamadpoor_2015 - The effect of ear acupressure on severity of pain in patients waiting for appendectomy	Y	Y	Y	Y	Y	N	only abstract
**425**	Mohseni-Bandpei_2000 - Effects of Spinal Manipulation Therapy in the Treatment of Chronic Low Back Pain: A Randomised Clinical Trial	Y	Y	Y	N	N	N	ultrasound
**426**	**Molina-Ortega_2014 - Immediate effects of spinal manipulation on nitric oxide, substance P and pain perception**	**Y**	**Y**	**Y**	**Y**	**N**	**Y**	
**427**	**molinscubero_2014 - Changes in Pain Perception after Pelvis**	**Y**	**Y**	**Y**	**Y**	**N**	**Y**	
**428**	Montalva_2006 - The Effects of Massage Therapy on Tension-Type Headaches: A Placebo Controlled Trial.	Y	Y	Y	Y	N	N	Thesis
**429**	Morgan_1997 - A controlled trial of spinal manipulation in the management of hypertension	Y	Y	Y	Y	N	N	comparison
**430**	Morris_2016 - Physiotherapy and a Homeopathic Complex for Chronic Low-back Pain Due to Osteoarthritis: A Randomized, Controlled Pilot Study	Y	Y	Y	N	N	N	They are not testing physiotherapy but homeopathy
**431**	Mosler_2003 - The effect of soft tissue therapy on hip joint range of motion	Y	Y	Y	Y	Y	N	only abstract and there is no info about control!

	and symptoms and eggbeater kick performance in water polo players							
**432**	**Motealleh_2016 - The immediate effect of lumbopelvic manipulation on EMG of vasti and gluteus medius in athletes with patellofemoral pain syndrome: A randomized controlled trial.**	**Y**	**Y**	**Y**	**Y**	**N**	**Y**	
**433**	**Moulson_2006 - A preliminary investigation into the relationship between cervical snags and sympathetic nervous system activity in the upper limbs of an asymptomatic population**	**Y**	**Y**	**Y**	**Y**	**N**	**Y**	
**434**	Moustafa_2014 - Multimodal Treatment Program Comparing 2 Different Traction Approaches for Patients With Discogenic Cervical Radiculopathy: A Randomized Controlled Trial	Y	Y	Y	N	N	N	There is no placebo.
**435**	**moutzouri_2008 - The effects of the Mulligan Sustained Natural Apophyseal Glide**	**Y**	**Y**	**Y**	**Y**	**N**	**Y**	
**436**	**moutzouri_2012 - Investigation of the effects of a centrally applied lumbar sustained natural apophyseal glide mobilization on lower limb sympathetic nervous system activity in asymptomatic subjects**	**Y**	**Y**	**Y**	**Y**	**N**	**Y**	
**437**	Moutzouri_2011 - Effects of mulligan mobilisation technique SNAG applied on the lumbar spine in the sympathetic nervous system activity of lower limbs	Y	Y	Y	Y	N	N	only abstract

**438**	Munday_2007 - A randomized, single-blinded, placebo-controlled clinical trial to evaluate the efficacy of chiropractic shoulder girdle adjustment in the treatment of shoulder impingement syndrome	Y	Y	Y	N	N	N	
**439**	Murphy_1995 - Sacroiliac joint manipulation decreases the H-reflex	N	Y	N	N	N	N	
**440**	**Naeimi_2012 - A Randomized Clinical Trial of the Efficacy of KID21 Point (Youmen) Acupressure on Nausea and Vomiting of Pregnancy**	**Y**	**Y**	**Y**	**Y**	**N**	**Y**	
**441**	**Nansel_1993 - Effect of cervical spinal adjustments on lumbar**	**Y**	**Y**	**Y**	**Y**	**N**	**Y**	
**442**	nasiri_2016 - Effect of aromatherapy massage with lavender essential oil on pain in patients with	Y	Y	Y	N	N	N	
**443**	Navarro_2009 - Eficacia de la técnica osteopática de liberación del hueso lagrimal en la obstrucción congénita del conducto nasolagrimal	Y	Y	Y	Y	N	N	osteopathy
**444**	Nct_2008 - Efficacy of Myofascial Trigger Point Pressure Release on Tinnitus Patients With Both Tinnitus and Myofascial Pain: a Double-blind Placebo Controlled Randomized Clinical Trial.	Y	Y	Y	Y	N	N	paper not found
**445**	niemisto_2003 - A Randomized Trial of Combined Manipulation	Y	Y	Y	N	N	N	

**446**	Noga_2002 - Acupressure as an adjunct to pharmacologic control of nausea, vomiting and retching (N/V) during blood and marrow transplantation (BMT): a randomized, placebo-controlled, algorithm based study.	Y	Y	Y	Y	Y	N	It's a Proceedings not a paper
**447**	nourbakhsh_2008 - The Effect of Oscillating-energy Manual Therapy	Y	Y	Y	Y	N	N	osteopathy
**448**	**Oliveira-Campelo_2013 - Short- and medium-term effects of manual therapy on cervical active range of motion and pressure pain sensitivity in latent myofascial pain of the upper trapezius muscle: a randomized controlled trial**	**Y**	**Y**	**Y**	**Y**	**N**	**Y**	
**449**	O'Mathúna_2009 - Massage-like therapeutic touch produces no significant differences in relieving behavioural symptoms of dementia or Cortisol compared with placebo therapeutic touch	Y	Y	Y	Y	Y	N	Paper not found
**450**	activator instrument	Y	Y	Y	N	N	N	
**451**	Otaño_2010 - Modificaciones radiológicas del espacio entre el occipucio y el cuerpo del atlas tras una manipulación global (OAA) de Fryette	Y	Y	Y	Y	N	N	osteopathy
**452**	otsudo_2016 - The effectiveness of direct stretching on bilateral psoas major to the lumbar lordosis	Y	Y	Y	Y	N	N	paper not available, requested to authors sent on 03.11.17 and again on 25.11.2017, no answer

**453**	**Packer_2014 - Effects of upper thoracic manipulation on pressure pain sensitivity in women with temporomandibular disorder: A randomized, double-blind, clinical trial.**	**Y**	**Y**	**Y**	**Y**	**N**	**Y**	
**454**	Packer_2015 - Effect of upper thoracic manipulation on mouth opening and electromyographic activity of masticatory muscles in women with temporomandibular disorder: a randomized clinical trial	Y	Y	Y	Y	N	N	same as ID453
**455**	**Panagoupulos_2014 - Does the addition of visceral manipulation alter outcomes for patients with low back pain? A randomized placebo controlled trial**	**Y**	**Y**	**Y**	**Y**	**N**	**Y**	
**456**	parker_1980 - Why Does Migraine Improve During a Clinical Trial	Y	Y	Y	Y	N	N	comparison
**457**	**patterson_2008 - A novel clinical-trial design for the study of**	**Y**	**Y**	**Y**	**Y**	**N**	**Y**	
**458**	**Pecos-Martín_2015 - Immediate effects of thoracic spinal mobilisation on erector spinae muscle activity and pain in patients with thoracic spine pain: A preliminary randomised controlled trial.**	**Y**	**Y**	**Y**	**Y**	**N**	**Y**	
**459**	Pedramrazi_2015 - The effect of reflexology on quality of life of iranian patients with breast cancer	Y	Y	Y	Y	N	N	only abstract

460	Perry_2002 - Effects of Unilaterally Applied Lumbar Mobilisation Technique on Peripheral Sympathetic Activity in the Lower Limbs	Y	Y	Y	Y	Y	N	only abstract
461	**perry_2008 - An investigation into the effects of a unilaterally applied lumbar**	**Y**	**Y**	**Y**	**Y**	**N**	**Y**	
462	perry_2011 - A preliminary investigation into the magnitude of effect of lumbar extension	Y	Y	Y	N	N	N	comparison
463	Perry_2015 - A randomised, independent groups study investigating the sympathetic nervous system responses to two manual therapy treatments in patients with LBP	Y	Y	Y	N	N	N	indipendent randomized trial, control between two manual therapies
464	**Pertille_2012 - Immediate effects of bilateral grade III mobilization of the talocrural joint on the balance of elderly women**	**Y**	**Y**	**Y**	**Y**	**N**	**Y**	
465	Petersen_1992 - Foot zone therapy and bronchial asthma--a controlled clinical trial	Y	Y	Y	N	N	N	no manual control
466	**Petersen_1993 - The effects of a cervical mobilisation technique on sympathetic outflow to the upper limb in normal subjects**	**Y**	**Y**	**Y**	**Y**	**N**	**Y**	
467	Pichonnaz_2016 - Effect of Manual Lymphatic Drainage After Total Knee Arthroplasty: A Randomized Controlled Trial	Y	Y	Y	N	N	N	

**468**	Pichonnaz_2015 **-** Effects of manual lymphatic drainage following total knee arthroplasty: a prospective randomised controlled trial	Y	Y	Y	N	N	N	
**469**	**Piekarz_2016 - An investigation into the effects of applying a lumbar Maitland mobilisation at different frequencies on sympathetic nervous system activity levels in the lower limb.**	**Y**	**Y**	**Y**	**Y**	**N**	**Y**	
**470**	Pikula_1999 **-** The effect of spinal manipulative therapy (SMT) on pain reduction and range of motion in patients with acute unilateral neck pain: a pilot study.	Y	Y	Y	Y	Y	N	confronting threating one side versus threating the other side
**471**	**pires_2015 - Immediate and Short-Term Effects of Upper Thoracic Manipulation on Myoelectric Activity of Sternocleidomastoid**	**Y**	**Y**	**Y**	**Y**	**N**	**Y**	
**472**	plaugher_2002 **-** Practice-based randomized controlled-comparison clinical trial of chiropractic adjustments	Y	Y	Y	N	N	N	
**473**	**Pollard_1998 - The effect of upper cervical or sacroiliac manipulation on hip flexion range of motion**	**Y**	**Y**	**Y**	**Y**	**N**	**Y**	
**474**	**Pouresmail_2002 - Effects of acupressure and ibuprofen on the severity of primary dysmenorrhe**	**Y**	**Y**	**Y**	**Y**	**N**	**Y**	

475	Preyde_2000 - Effectiveness of massage therapy for subacute low-back pain: a randomized controlled trial	Y	Y	Y	N	N	N	They use soft-tissue manipulation and laser therapy as sham: excluded
**476**	**Puentedura_2011 - Immediate effects of lumbar spine manipulation on the resting and contraction thickness of transversus abdominis in asymptomatic individuals**	**Y**	**Y**	**Y**	**Y**	**N**	**Y**	
**477**	**Puhl_2012 - Short-term effects of manipulation to the upper thoracic spine of asymptomatic subjects on plasma concentrations of epinephrine and norepinephrine-a randomized and controlled observational study**	**Y**	**Y**	**Y**	**Y**	**N**	**Y**	
478	Puhl_2016 - The quality of placebos used in randomized, controlled trials of lumbar and pelvic joint thrust manipulation - a systematic review	N	N	N	N	N	N	
**479**	**Purdy_1996 - Suboccipital dermatomyotomic stimulation and digital blood flow**	**Y**	**Y**	**Y**	**Y**	**N**	**Y**	
**480**	**Putt_2008 - Muscle Stretching Technique Increases Vital Capacity and Range of Motion in Patients With Chronic Obstructive Pulmonary Disease**	**Y**	**Y**	**Y**	**Y**	**N**	**Y**	
**481**	**Quinn_2008 - Reflexology in the management of low back pain: a pilot randomised controlled trial**	**Y**	**Y**	**Y**	**Y**	**N**	**Y**	

**482**	Reed_1988 - Effects of sequential connective tissue massage on autonomic nervous system of middle aged and eldery adults	Y	Y	Y	N	N	N	sham ultrasound: excluded
**483**	Reed_1994 - Chiropractic management of primary nocturnal enuresis	Y	Y	Y	N	N	N	
**484**	Reid_2014 - Effects of cervical spine manual therapy on range of motion, head repositioning, and balance in participants with cervicogenic dizziness: A randomized controlled trial.	N	Y	Y	N	N	N	Laser therapy as placebo
**485**	ridehalgh_2005 - Effect of straight leg raise examination and treatment on vibration	Y	Y	Y	Y	N	N	
**486**	riley_2015 - Thoracic spinal manipulation for musculoskeletal shoulder pain Can	Y	Y	Y	N	N	N	
**487**	**rocha_2012 - Efficacy of myofascial trigger point deactivation for tinnitus control**	**Y**	**Y**	**Y**	**Y**	**N**	**Y**	
**488**	**Rocha_2015 - The Manual Diaphragm Release Technique improves diaphragmatic mobility, inspiratory capacity and exercise capacity in people with chronic obstructive pulmonary disease: a randomised trial.**	**Y**	**Y**	**Y**	**Y**	**N**	**Y**	
**489**	**Rosa_2013 - Effect of seated thoracic manipulation on changes in scapular**	**Y**	**Y**	**Y**	**Y**	**N**	**Y**	

	**kinematics and scapulohumeral rhythm in young asymptomatic participants: a randomized study**							
**490**	Roshanravan_2016 - Effect of foot reflexology on fatigue in patients undergoing hemodialysis: A sham-controlled randomized trial	Y	Y	Y	Y	Y	N	Persian Language, english full text requested on 24/10/2017 and then again on 14.11.2017 , no answer
**491**	Roudsari_2015 - The effect of acupressure on pain intensity in primary dysmenorrhea	Y	Y	Y	Y	N	N	comparison beetween two acupressure point, no placebo control
**492**	Rowe_2006 - Chiropractic manipulation in adolescent idiopathic scoliosis: A pilot study	Y	Y	Y	N	N	N	
**493**	Roy_2009 - Heart rate variability modulation after manipulation in pain-free patients vs patients in pain	Y	Y	Y	Y	N	N	technique performed using an activator
**494**	**roy_2010 - Paraspinal cutaneous temperature modification after spinal**	**Y**	**Y**	**Y**	**Y**	**N**	**Y**	
**495**	Rubinstein_2012 - Is the methodological quality of trials on spinal manipulative therapy for low-back pain improving?	N	N	N	N	N	N	
**496**	Rubinstein_2014 - The Risk of Bias and Sample Size of Trials of Spinal Manipulative Therapy for Low Back and Neck Pain: Analysis and Recommendations	N	N	N	N	N	N	
**497**	Ruddock_2016 - Spinal Manipulation Vs Sham Manipulation for Nonspecific	N	N	N	N	N	N	

	Low Back Pain: A Systematic Review and Meta-analysis							
**498**	Ruiz-Saez_2007 - Changes in pressure pain sensitivity in latent myofascial trigger points in the upper trapezius muscle after a cervical spine manipulation in pain-free subjects.	Y	Y	Y	Y	N	N	osteopathy
**499**	**Sabouhi_2013 - Effect of acupressure on fatigue in patients on hemodialysis**	**Y**	**Y**	**Y**	**Y**	**N**	**Y**	
**500**	**Saiz-Llamosas_2009 - Changes in neck mobility and pressure pain threshold levels following a cervical myofascial induction technique in pain-free healthy subjects**	**Y**	**Y**	**Y**	**Y**	**N**	**Y**	
**501**	Sanchez Bayle_2012 - Estudio de la eficacia y utilidad de la fisioterapia respiratoria en la	Y	Y	Y	Y	N	N	The Authors, declared a placebo treatment, but it's a control treatment without manual touch.
**502**	Sánchez_2009 - Repercusiones sintomáticas de la técnica dog en extensión bilateral sobre D5D6 en pacientes dispépticos	Y	Y	Y	Y	N	N	osteopathy
**503**	Sanchez_2010 - Influencia de la técnica de bombeo del globo ocular en la presión	Y	Y	Y	Y	N	N	osteopathy
**504**	Sanders_1990 - The effect of spinal adjustive manipulation on subjects with acute low back pain: A comparison of visual analog pain scores and serum beta endorphin levels.	Y	Y	Y	Y	Y	N	full text requested via reserach gate on 03.11.2017 and again on 24.11.2017, no answer

**505**	**Sanders_2015 - Effects of Lumbosacral Manipulation on Isokinetic Strength of the Knee Extensors and Flexors in Healthy Subjects: A Randomized, Controlled, Single-Blind Crossover Trial**	**Y**	**Y**	**Y**	**Y**	**N**	**Y**	
**506**	Sanders_1990 - Chiropractic adjustive manipulation on subjects with acute low back pain: visual analog pain scores and plasma beta-endorphin levels	Y	Y	Y	Y	Y	N	same as ID504
**507**	**Santilli_2006 - Chiropractic manipulation in the treatment of acute back pain and sciatica with disc protrusion: a randomized double-blind clinical trial of active and simulated spinal manipulations.**	**Y**	**Y**	**Y**	**Y**	**N**	**Y**	
**508**	**Saranga_2003 - Effect of a Cervical Lateral Glide on the Upper Limb Neurodynamic Test 1: A blinded placebo-controlled investigation**	**Y**	**Y**	**Y**	**Y**	**N**	**Y**	
**509**	**Sawyer_1999 - A feasibility study of chiropractic spinal manipulation versus sham spinal manipulation for chronic otitis media with effusion in children**	**Y**	**Y**	**Y**	**Y**	**N**	**Y**	
**510**	Schiller_2001 - Effectiveness of spinal manipulative therapy in the treatment of mechanical thoracic spine pain: a pilot randomized clinical trial	Y	Y	Y	N	N	N	detuned ultrasound as sham
**511**	Scholten-Peeters_2013 - Is manipulative therapy more effective	N	N	N	N	N	N	

	than sham manipulation in adults?: A systematic review and meta-analysis						
512	**Selhorst_2015 - Lumbar manipulation and exercise for the treatment of acute low back pain in adolescents: A randomized controlled trial**	**Y**	**Y**	**Y**	**Y**	**N**	**Y**
513	**Selkow_2009 - Short-term effect of muscle energy technique on pain in individuals with non-specific lumbopelvic pain: a pilot study**	**Y**	**Y**	**Y**	**Y**	**N**	**Y**
514	**senna_2011 - Does Maintained Spinal Manipulation Therapy**	**Y**	**Y**	**Y**	**Y**	**N**	**Y**
515	Shearar_2005 - A Randomized Clinical Trial of Manual Versus Mechanical Force Manipulation in the Treatment of Sacroiliac Joint Syndrome	Y	Y	Y	N	N	N
516	**Shin_2016 - The immediate effects of spinal thoracic manipulation on respiratory functions**	**Y**	**Y**	**Y**	**Y**	**N**	**Y**
517	**shin_2007 - Effect of Nei–Guan point (P6) acupressure on ketonuria levels,**	**Y**	**Y**	**Y**	**Y**	**N**	**Y**
518	Shrier_2006 - A pilot study on the effects of pre-event manipulation on jump height and running velocity	Y	Y	Y	N	N	N
519	**sievner_2003 - Reflexology treatment relieves symptoms of multiple sclerosis**	**Y**	**Y**	**Y**	**Y**	**N**	**Y**

**520**	Silva_2015 - Evaluation of the Immediate Effect of Auricular Acupuncture on Pain and Electromyographic Activity of the Upper Trapezius Muscle in Patients with Nonspecific Neck Pain: A Randomized, Single-Blinded, Sham-Controlled, Crossover Study	Y	Y	Y	N	N	N	A physiotherapist attended control group participants for the same period but only answered questions
**521**	**Simon_1997 - The influence of an anteroposterior accessory glide of the glenohumeral joint on measures of peripheral sympathetic nervous system function in the upper limb**	**Y**	**Y**	**Y**	**Y**	**N**	**Y**	
**522**	**Slater_2006 - Effects of a manual therapy technique in experimental lateral epicondylalgia**	**Y**	**Y**	**Y**	**Y**	**N**	**Y**	
**523**	**Smith_2016 - Effect of a lateral glide mobilisation with movement of the hip on vibration threshold in healthy volunteers**	**Y**	**Y**	**Y**	**Y**	**N**	**Y**	
**524**	**Sousa_2015 - Immediate effects of Tuina techniques on working-related musculoskeletal disorder of professional orchestra musicians**	**Y**	**Y**	**Y**	**Y**	**N**	**Y**	
**525**	**Srbely_2013 - Immediate effects of spinal manipulative therapy on regional antinociceptive effects in myofascial tissues in healthy young adults**	**Y**	**Y**	**Y**	**Y**	**N**	**Y**	

**526**	**sterling_2010 - Cervical lateral glide increases nociceptive flexion reflex threshold but not**	**Y**	**Y**	**Y**	**Y**	**N**	**Y**	
**527**	straub_2001 - The Effect of Chiropractic Care on Jet Lag of Finnish Junior Elite Athletes	Y	Y	Y	Y	N	N	non-manual sham
**528**	Suh_2012 - The effects of P6 acupressure and nurse-provided counseling on chemotherapy-induced nausea and vomiting in patients with breast cancer	Y	N	Y	N	N	N	wristband
**529**	**Sun_2010 - Effectiveness of acupressure for residents of long-term care facilities with insomnia: A randomized controlled trial**	**Y**	**Y**	**Y**	**Y**	**N**	**Y**	
**530**	**surenkok_2009 - Acute Effects of Scapular Mobilization**	**Y**	**Y**	**Y**	**Y**	**N**	**Y**	
**531**	Tang_2012 - The effects of acupressure on fatigue of lung cancer patients undergoing chemotherapy: A double-blind experimental study	Y	Y	Y	Y	Y	N	same as ID532
**532**	**Tang_2014 - Effects of acupressure on fatigue of lung cancer patients undergoing chemotherapy: An experimental pilot study**	**Y**	**Y**	**Y**	**Y**	**N**	**Y**	
**533**	Tara_2015 - Effects of pressure stimulation of the nei guan (PC6) point on the nausea and vomiting in pregnant women	Y	Y	Y	Y	Y	N	presented at a congress, not published

**534**	tekeoglu_1996 - Suppression of Experimental Pain	Y	Y	Y	Y	N	N	presented at a congress, not published
**535**	**Teodorczyk-Injeyan_2006 - Spinal manipulative therapy reduces inflammatory cytokines but not substance P production in normal subjects**	**Y**	**Y**	**Y**	**Y**	**N**	**Y**	
**536**	**Teys_2008 - The initial effects of a Mulligan's mobilization with movement technique on range of movement and pressure pain threshold in pain-limited shoulders**	**Y**	**Y**	**Y**	**Y**	**N**	**Y**	
**537**	Thomason_1979 - Effectiveness of spinal manipulative therapy in treatment of primary dysmenorrhea: A pilot study	Y	Y	Y	N	N	N	
**538**	Thomson_2009 - The effects of high-velocity low-amplitude thrust manipulation and mobilisation techniques on pressure pain threshold in the lumbar spine.	Y	Y	Y	N	N	N	They compare two approaches and use sham laser procedure: excluded
**539**	Tian_2006 - Efficacy of auricular acupressure as an adjuvant therapy in substance abuse treatment: a pilot study	Y	Y	Y	N	N	N	Seed: Vaccaria hispancia
**540**	**Torkzahrani_2016 - The effect of acupressure on the initiation of labor: A randomized controlled trial**	**Y**	**Y**	**Y**	**Y**	**N**	**Y**	
**541**	**Touche_2013 - Does Mobilization of the Upper Cervical Spine Affect Pain**	**Y**	**Y**	**Y**	**Y**	**N**	**Y**	

**542**	**Tsay_2004 - Acupressure and fatigue in patients with end-stage renal disease–a randomized controlled trial**	**Y**	**Y**	**Y**	**Y**	**N**	**Y**	
**543**	Valiee_2012 - Effect of Acupressure on Preoperative Anxiety: A Clinical Trial	Y	Y	Y	N	N	N	plastic tool
**544**	Vecino_2009 - Modifi caciones inmediatas en la dinámica uterina tras	Y	Y	Y	Y	N	N	osteopathy
**545**	**Vernon_2009 - A randomized, placebo-controlled clinical trial of chiropractic and medical prophylactic treatment of adults with tension-type headache: results from a stopped trial**	**Y**	**Y**	**Y**	**Y**	**N**	**Y**	
**546**	Vernon_2011 - Systematic review of clinical trials of cervical manipulation: Control group procedures and pain outcomes	N	N	N	N	N	N	
**547**	Vernon_1986 - Spinal manipulation and beta-endorphin: a controlled study of the effect of a spinal manipulation on plasma beta-endorphin levels in normal males.	Y	Y	Y	Y	Y	N	not found
**548**	**Vicenzino_1998 - Cardiovascular and respiratory changes produced by lateral glide mobilization of the cervical spine**	**Y**	**Y**	**Y**	**Y**	**Y**	**Y**	
**549**	Vicenzino_1999 - An investigation of stress and pain perception during manual therapy in asymptomatic subjects	Y	Y	Y	Y	N	N	same data set as ID548

**550**	Vicenzino_1998 - An investigation of the interrelationship between manipulative therapy-induced hypoalgesia and sympathoexcitation	Y	Y	Y	Y	Y	N	not found
**551**	**vicenzino_1996 - The initial effectsof a cervical spine manipulative physiotherapy**	**Y**	**Y**	**Y**	**Y**	**N**	**Y**	
**552**	**vicenzino_2001 - Specific manipulative therapy treatment for chronic lateral epicondylalgia**	**Y**	**Y**	**Y**	**Y**	**N**	**Y**	
**553**	**vieirapellenz_2014 - Short-Term Effect of Spinal Manipulation on Pain Perception, Spinal Mobility, and Full**	**Y**	**Y**	**Y**	**Y**	**N**	**Y**	
**554**	villafane_2013 - The Effectiveness of a Manual Therapy	Y	Y	Y	N	N	N	
**555**	Vincenzo_2016 - Short-Term Effect of BalanceWear Therapy on Mobility in Older Adults With Mobility Limitations	Y	Y	Y	Y	Y	N	balance wear therapy is not a manual treatment
**556**	Volkening_2000 - The short-term effect of spinal manipulation in the treatment ofinfantile colic: A randomized controlled clinical trial with a blindedobserver	N	N	N	N	N	N	comment
**557**	Wakefield - 2000 - Evidence-based Physiotherapy: The case for pragmatic randomised controlled trials	N	N	N	N	N	N	
**558**	walker_2013 - Outcomes of Usual Chiropractic. The OUCH	Y	Y	Y	N	N	N	They use an activator

**559**	Wang_2015 - Manual therapy for hip osteoarthritis: A systematic review and meta-analysis	N	N	N	N	N	N	
**560**	Warwick-Evans_1991 - A double-blind placebo controlled evaluation of acupressure in the treatment of motion sickness	Y	Y	Y	Y	Y	N	not found
**561**	Webb_2016 - Myofascial techniques: What are their effects on joint range of motion and pain? – A systematic review and meta-analysis of randomised controlled trial"	N	N	N	N	N	N	
**562**	Whittingham_2002 - Randomized, placebo-controlled clinical trial of the efficacy of chiropractic treatment for chronic cervicogenic headaches	Y	Y	Y	Y	Y	N	only abstract, even in SR is cited as an abstract
**563**	Whittingham_2001 - Active range of motion in the cervical spine increases after spinal manipulation (toggle recoil)	Y	Y	Y	Y	N	N	non-manual sham, instrument used
**564**	wilder_2011 - Effect of spinal manipulation on sensorimotor	Y	Y	Y	Y	N	N	non-manual sham, they use an activator with a rubber guard
**565**	win_2015 - Effects of Upper and Lower Cervical Spinal	Y	Y	Y	N	N	N	
**566**	Wong_2014 - Strain counterstrain technique to decrease tender point palpation pain compared to control conditions: A systematic review with meta-analysis	N	N	N	N	N	N	

567	**wong_2010 - The effects of manual treatment on rounded shoulder**	**Y**	**Y**	**Y**	**Y**	**N**	**Y**	
568	wong_2015 - Self-administered acupressure	Y	Y	Y	Y	N	N	acupressure applied through a device (Acupen)
569	**woods_2009 - The Effect of Therapeutic Touch on Behavioral**	**Y**	**Y**	**Y**	**Y**	**N**	**Y**	
570	wu_2004 - Effectiveness of acupressure in improving dyspnoea in chronic	Y	Y	Y	Y	N	N	"sham acupoints" are real acupoints involved in intestinal movements and not respiratory function (treatment group)
571	Xu_2016 - Effect of massage therapy on pulmonary functions of pediatric asthma: A systematic review and meta-analysis of randomized controlled trials.	N	N	N	N	N	N	
572	Yamato_2016 - How completely are physiotherapy interventions described in reports of randomised trials?	N	N	N	N	N	N	
573	yang_2010 - Effect of Acupressure on Thirst in	Y	Y	Y	Y	N	N	"stickers" on the interested acupoints are used as sham acupressure
574	**yang_2016 - Effectiveness and safety of**	**Y**	**Y**	**Y**	**Y**	**N**	**Y**	
575	Yates_1988 - Effects of chiropractic treatment on blood pressure and anxiety: a randomized, controlled trial	Y	Y	Y	Y	Y	N	treatment is delivered through device
576	Yeh_2012 - Auricular point acupressure for chronic low back pain: A feasibility study for 1-week treatment	Y	N	Y	N	N	N	manual pressure through a tape that blocks vaccaria seed (excluded as all studied of this

								author that use the same method)
577	Yung_2016 - A randomized, placebo-controlled, cardiovascular response comparison of unilateral posterior-to-anteriorly directed mobilization of the neck in pain-free adults	Y	Y	Y	Y	N	N	same as ID578
**578**	**yung_2014 - Blood Pressure and Heart Rate Response**	**Y**	**Y**	**Y**	**Y**	**N**	**Y**	
579	zegarraparodi_2016 - Effects of Pressure Applied During Standardized Spinal Mobilizations on Peripheral	Y	Y	Y	Y	N	N	osteopathy
580	Touche_2013 - Does Mobilization of the Upper Cervical Spine Affect Pain	Y	Y	Y	Y	N	N	same as ID541
**581**	**Aghdam_2012 - Effect of acupressure at Hugo point (LI4) on the process and outcomes of labor in nulliparous women**	**Y**	**Y**	**Y**	**Y**	**Y**	**Y**	
**582**	**Beneciuk_2009 - Effects of upper extremity neural mobilization on thermal pain sensitivity: A sham-controlled study in asymptomatic participants**	**Y**	**Y**	**Y**	**Y**	**Y**	**Y**	
583	Briggs_1988 - Effects of a chiropractic adjustment on changes in pupillary diameter: a model for evaluating somatovisceral response	Y	Y	Y	Y	Y	N	not found
**584**	**Brygge_2001 - Reflexology and bronchial asthma**	**Y**	**Y**	**Y**	**Y**	**Y**	**Y**	

**585**	**Cardinale_2015 - The acute effects of spinal manipulation on neuromuscular function in asymptomatic individuals: A preliminary study**	**Y**	**Y**	**Y**	**Y**	**Y**	**Y**	
**586**	**Chaibi_2015 - Validation of placebo in a manual therapy randomized controlled trial**	**Y**	**Y**	**Y**	**Y**	**Y**	**Y**	
**587**	Clinch_1986 - The effect of grade II mobilization on range of movement in acutely sprained ankles	N	N	N	N	N	N	not found
**588**	**Diego_2005 - Vagal activity, gastric motility, and weight gain in massaged preterm neonates**	**Y**	**Y**	**Y**	**Y**	**Y**	**Y**	
**589**	**Fernandez-de-las-Penas_2007 - Immediate effects on pressure pain threshold following a single cervical spine manipulation in healthy subjects**	**Y**	**Y**	**Y**	**Y**	**Y**	**Y**	
**590**	Fisher_2009 - Identification of potential neuromotor mechanisms of manual therapy in patients with musculoskeletal disablement: rationale and description of a clinical trial	Y	Y	Y	Y	Y	N	description of a potential trial
**591**	Ghavami_2016 - The effect of applying reflexology massage on Nitroglycerin Induced Migraine Type Headache	Y	Y	Y	Y	Y	N	first mail sent to corresponding author on 16.02.2018, second mail on 10.03.2018, no answer
**592**	Grossi_1981 - Effects of an applied kinesiology technique on quadriceps femoris muscle isometric strength	Y	Y	Y	Y	Y	N	no direct contact but transducers

593	Hebron_2015 - A randomised placebo-controlled study investigating the effects of mobilisation treatment duration on pain in participants with chronic low back pain	Y	Y	Y	N	Y	N	
**594**	**Hodgson_2000 - Does reflexology impact on cancer patients' quality of life?**	**Y**	**Y**	**Y**	**Y**	**Y**	**Y**	
595	Jenkins_1995 - An Investigation into the Effects of a Cervical and Thoracic Mobilisation Technique on Blood Pressure, Heart Rate and Upper Limb Sympathetic Outflow	Y	Y	Y	Y	Y	N	Abstract meeting of the Scottish Physiotherapists Research Workshop on October 6, 1995, at the University of Stirling.
**596**	**Kafaei-Atrian_2016 - The effect of acupressure at third liver point on the anxiety level in patients with primary dysmenorrhea**	**Y**	**Y**	**Y**	**Y**	**Y**	**Y**	
**597**	**Kim_2012 - Improvement of pain and functional activities in patients with lateral epicondylitis of the elbow by mobilization with movement: a randomized, placebo-controlled pilot study**	**Y**	**Y**	**Y**	**Y**	**Y**	**Y**	
**598**	**Lang_2007 - Prehospital analgesia with acupressure at the Baihui and Hegu points in patients with radial fractures: a prospective, randomized, double-blind trial**	**Y**	**Y**	**Y**	**Y**	**Y**	**Y**	
**599**	**Maa_2007 - Self-administered acupressure reduces the symptoms that limit daily activities in**	**Y**	**Y**	**Y**	**Y**	**Y**	**Y**	

	**bronchiectasis patients: pilot study findings**							
600	Maffei_2014 - Intérêt du massage dans le traitement de douleurs postopératoires précoces : étude contrôlée randomisée	N	N	N	N	N	N	comparison between two different post-op massages, no control
601	**Kojidi_2016 - The influence of Positional Release Therapy on the myofascial trigger points of the upper trapezius muscle in computer users**	Y	Y	Y	Y	Y	Y	
602	**Molassiotis_2007 - The management of cancer-related fatigue after chemotherapy with acupuncture and acupressure: a randomised controlled trial**	Y	Y	Y	Y	Y	Y	
603	**Mollart_2016 - A feasibility randomised controlled trial of acupressure to assist spontaneous labour for primigravid women experiencing a post-date pregnancy**	Y	Y	Y	Y	Y	Y	
604	**Olafsdottir_2001 - Randomised controlled trial of infantile colic treated with chiropractic spinal manipulation**	Y	Y	Y	Y	Y	Y	
605	**Oleson_1997 - Randomized controlled study of premenstrual symptoms treated with ear, hand and foot reflexology**	Y	Y	Y	Y	Y	Y	
606	Paumard_2006 - Une manipulation de la colonne thoracique améliore des	N	N	N	N	N	N	comment on ID607

	douleurs cervicales, au moins immédiatement après							
**607**	Cleland_2005-Immediate effects of thoracic manipulation in patients with neck pain: a randomized clinical trial	N	N	N	N	N	N	same as ID163
**608**	Paumard_2006 - Des mobilisations-manipulations des articulations de la cheville éliminent les symptômes séquellaires d’entorse de cheville non améliorés par le traitement conventionnel	N	N	N	N	N	N	comment on ID609
**609**	Whitman_2005-The use of manipulation in a patient with an ankle sprain injury not respondingto conventional management: a case report	N	N	N	N	N	N	case report
**610**	Paumard_2007 - La technique de Mitchell (Muscle energy technique, sorte de contracté-relâché) permet d’obtenir sur des sujets sains une meilleure mobilité au niveau de la région cervicale, contrairement à des manœuvres passives soutenues qui diminuent un peu les amplitudes initiales	N	N	N	N	N	N	comment on ID611
**611**	Burns_2006-Gross range of motion in the cervical spine: the effects of osteopathic muscle energy technique in asymptomatic subjects	N	N	N	N	N	N	osteopathy
**612**	**Paungmali_2003 - Hypoalgesic and sympathoexcitatory effects of mobilization with movement for lateral epicondylalgia**	**Y**	**Y**	**Y**	**Y**	**Y**	**Y**	

**613**	**Rad_2012 - A randomized clinical trial of the efficacy of KID21 point (Youmen) acupressure on nausea and vomiting of pregnancy**	**Y**	**Y**	**Y**	**Y**	**Y**	**Y**	
**614**	Saatsaz_2016 - Massage as adjuvant therapy in the management of post-cesarean pain and anxiety: A randomized clinical trial.	Y	Y	Y	Y	Y	N	There isn't a manual control group
**615**	**Tsirakis_2015 - The effects of a modified spinal mobilisation with leg movement (SMWLM) technique on sympathetic outflow to the lower limbs**	**Y**	**Y**	**Y**	**Y**	**Y**	**Y**	
**616**	Vernon_2013 - Retention of blinding at follow-up in a randomized clinical study using a sham-control cervical manipulation procedure for neck pain: secondary analyses from a randomized clinical study	Y	Y	Y	Y	Y	N	same dataset as ID617
**617**	**Vernon_2012 - Validation of a novel sham cervical manipulation procedure**	**Y**	**Y**	**Y**	**Y**	**Y**	**Y**	
**618**	Vernon_2005 - Validation of a sham manipulative procedure for the cervical spine for use in clinical trials	N	N	N	N	N	N	only sham!
**619**	**Beikmoradi_2014 - Acupressure and anxiety in cancer patients**	**Y**	**Y**	**Y**	**Y**	**Y**	**Y**	
**620**	Blanchard_2015 - In adults with painful hip osteoarthritis physical therapy did	Y	Y	Y	Y	Y	N	comment

	not lead to better improvements in pain or function than sham therapy							
**621**	Kivlan_2015 - The effect of Astym(R) Therapy on muscle strength: a blinded, randomized, clinically controlled trial	Y	N	Y	Y	Y	N	instrument assisted
**622**	Lin_2015 - The Anti-Inflammatory Actions of Auricular Point Acupressure for Chronic Low Back Pain	Y	Y	Y	Y	Y	N	seeds taped
**623**	**Martin-Pintado-Zugasti_2015 - Ischemic Compression After Dry Needling of a Latent Myofascial Trigger Point Reduces Postneedling Soreness Intensity and Duration**	**Y**	**Y**	**Y**	**Y**	**Y**	**Y**	
**624**	Romero Morales_2015 - Efectividad de las técnicas de manipulación cervical vs. técnica de compresión en puntos gatillo en pacientes con cefalea tensional	Y	Y	Y	Y	Y	N	control group
**625**	Yeh_2015 - Acupoint Stimulation on Weight Reduction for Obesity: A Randomized Sham-Controlled Study	Y	Y	Y	Y	Y	N	seed and electrical acupressure
**626**	**Cruz-Montecinos_2016 - Changes in co-contraction during stair descent after manual therapy protocol in knee osteoarthritis: A pilot, single-blind, randomized study**	**Y**	**Y**	**Y**	**Y**	**Y**	**Y**	
**627**	Goertz_2016 - Effects of spinal manipulation on sensorimotor function in low back pain patients -- a randomised controlled trial	Y	Y	Y	Y	Y	N	activator device

**628**	Li_2016 **-** Feasibility of a Randomized Controlled Trial of Self-Administered Acupressure for Symptom Management in Older Adults with Knee Osteoarthritis	Y	Y	Y	Y	Y	N	hand-held device
**629**	Luo_2016 **-** The effect of auricular acupressure on preoperative anxiety in patients undergoing gynecological surgery.	Y	Y	Y	Y	Y	N	adhesive plaster
**630**	Kojidi_2016 **-** The influence of Positional Release Therapy on the myofascial trigger points of the upper trapezius muscle in computer users	Y	Y	Y	Y	Y	N	Same as ID601
**631**	**Mohmadi_2016 - The effect of acupressure on muscle cramps in patients undergoing hemodialysis**	**Y**	**Y**	**Y**	**Y**	**Y**	**Y**	
**632**	**Sohns_2016 Manual trigger point therapy of shoulder pain : Randomized controlled study of effectiveness**	**Y**	**Y**	**Y**	**Y**	**Y**	**Y**	
**633**	Yeh_2016 - Changes in Sleep With Auricular Point Acupressure for Chronic Low Back Pain	Y	Y	Y	Y	Y	N	vaccaria seeds
**634**	Alonso-Perez_2017 **-** Hypoalgesic effects of three different manual therapy techniques on cervical spine and psychological interaction: A randomized clinical trial	Y	Y	Y	Y	Y	N	COMPARISON
**635**	**Arguisuelas_2017 - Effects of myofascial release in non-specific**	**Y**	**Y**	**Y**	**Y**	**Y**	**Y**	

	**chronic low back pain: a randomized clinical trial**							
**636**	Armand_2017 - Effect of Acupressure on Early Complications of Menopause in Women Referring to Selected Health Care Centers." Iran J Nurs Midwifery Res 22(3): 237-242.	Y	Y	Y	Y	Y	N	wrist brace
**637**	**Bautista-Aguirre_2017 - Effect of cervical vs. thoracic spinal manipulation on peripheral neural features and grip strength in subjects with chronic mechanical neck pain: a randomized controlled trial**	**Y**	**Y**	**Y**	**Y**	**Y**	**Y**	
**638**	**Bazarganipour_2017 - The effect of applying pressure to the LIV3 and LI4 on the symptoms of premenstrual syndrome: A randomized clinical trial**	**Y**	**Y**	**Y**	**Y**	**Y**	**Y**	
**639**	Bazarganipour_2017 - A randomized controlled clinical trial evaluating quality of life when using a simple acupressure protocol in women with primary dysmenorrhea	Y	Y	Y	Y	Y	N	same as ID638
**640**	**Bowler_2017 - The effects of cervical sustained natural apophyseal glides on neck range of movement and sympathetic nervous system activity." International Journal of Osteopathic Medicine 25: 15-20.**	**Y**	**Y**	**Y**	**Y**	**Y**	**Y**	
**641**	Capo-Juan_2017 - Short term effectiveness of pressure release and Kinesiotaping in cervical myofascial	Y	Y	Y	Y	Y	N	Application of algometric bilateral pressure for the placebo group

	pain caused by sternocleidomastoid muscle: a randomized clinical trial							
**642**	Cha_2017 - Effects of Auricular Acupressure Therapy on Stress and Sleep Disturbance of Middle-Aged Women in South Korea	Y	Y	Y	Y	Y	N	acupressure needle and skin paper tape
**643**	**Chaibi_2017 - Chiropractic spinal manipulative therapy for migraine: a three-armed, single-blinded, placebo, randomized controlled trial**	**Y**	**Y**	**Y**	**Y**	**Y**	**Y**	
**644**	Chaibi_2017 - Adverse events in a chiropractic spinal manipulative therapy single-blinded, placebo, randomized controlled trial for migraineurs	Y	Y	Y	Y	Y	N	same as ID643
**645**	**Ditcharles_2017 - Short-Term Effects of Thoracic Spine Manipulation on the Biomechanical Organisation of Gait Initiation: A Randomized Pilot Study**	**Y**	**Y**	**Y**	**Y**	**Y**	**Y**	
**646**	**Félix_2017 - The acute effect of Bowen therapy on pressure pain thresholds and postural sway in healthy subjects**	**Y**	**Y**	**Y**	**Y**	**Y**	**Y**	
**647**	Feng_2017 - Auricular Acupressure in the Prevention of Postoperative Nausea and Emesis A Randomized Controlled Trial	Y	Y	Y	Y	Y	N	requested via researchgate on 20.08.2019 and on 10.09.2019, no answer
**648**	Ferragut-Garcias, A., G. Plaza-Manzano, et al. (2017). "Effectiveness of a Treatment Involving Soft Tissue Techniques and/or Neural Mobilization	Y	Y	Y	Y	Y	N	same as ID216

	Techniques in the Management of Tension-Type Headache: A Randomized Controlled Trial." Arch Phys Med Rehabil 98(2): 211-219 e212.							
**649**	Fryer_2017 - Acute electromyographic responses of deep thoracic paraspinal muscles to spinal manual therapy interventions. An experimental, randomized cross-over study	Y	Y	Y	Y	Y	N	OSTEOPATHY
**650**	**Haik_2017 - Short-Term Effects of Thoracic Spine Manipulation on Shoulder Impingement Syndrome: A Randomized Controlled Trial**	**Y**	**Y**	**Y**	**Y**	**Y**	**Y**	
**651**	**Hauswirth_2017 - Immediate effects of cervical unilateral anterior-posterior mobilisation on shoulder pain and impairment in post-operative arthroscopy patients**	**Y**	**Y**	**Y**	**Y**	**Y**	**Y**	
**652**	**Kamali_2017 - The immediate effect of talocrural joint manipulation on functional performance of 15-40 years old athletes with chronic ankle instability: A double-blind randomized clinical trial**	**Y**	**Y**	**Y**	**Y**	**Y**	**Y**	
**653**	**Krekoukias_2017 - Spinal mobilization vs conventional physiotherapy in the management of chronic low back pain due to spinal disk degeneration: a randomized controlled trial**	**Y**	**Y**	**Y**	**Y**	**Y**	**Y**	
**654**	**Lai_2017 - Acupressure, Sleep, and Quality of Life in Institutionalized**	**Y**	**Y**	**Y**	**Y**	**Y**	**Y**	

	**Older Adults: A Randomized Controlled Trial**							
**655**	Lee_2017 **-** The Effects of Auricular Acupressure on Smoking Cessation for Male College Students	Y	Y	Y	Y	Y	N	use of seeds
**656**	**Macznik_2017 - Does Acupressure Hit the Mark? A Three-Arm Randomized Placebo-Controlled Trial of Acupressure for Pain and Anxiety Relief in Athletes With Acute Musculoskeletal Sports Injuries**	**Y**	**Y**	**Y**	**Y**	**Y**	**Y**	
**657**	**Mehyar_2017 - Immediate Effect of Lumbar Mobilization on Activity of Erector Spinae and Lumbar Multifidus Muscles**	**Y**	**Y**	**Y**	**Y**	**Y**	**Y**	
**658**	**Movahedi_2017 - The Effects of Acupressure on Pain Severity in Female Nurses with Chronic Low Back Pain**	**Y**	**Y**	**Y**	**Y**	**Y**	**Y**	
**659**	Oliva Pascual-Vaca_2017 **-** Short-Term Changes in Algometry, Inclinometry, Stabilometry, and Urinary pH Analysis After a Thoracolumbar Junction Manipulation in Patients with Kidney Stones	Y	Y	Y	Y	Y	N	Osteopathy
**660**	Pecos-Martin_2017 **-** Immediate effects of thoracic spinal mobilisation on erector spinae muscle activity and pain in patients with thoracic spine pain: a preliminary randomised controlled trial	Y	Y	Y	Y	N	N	same as ID458

**661**	**Pena-Salinas_2017 - No immediate changes on neural and muscular mechanosensitivity after first rib manipulation in subjects with cervical whiplash: A randomized controlled trial**	**Y**	**Y**	**Y**	**Y**	**Y**	**Y**	
**662**	**Powden_2017 - The Effect of 2 Forms of Talocrural Joint Traction on Dorsiflexion Range of Motion and Postural Control in Those With Chronic Ankle Instability**	**Y**	**Y**	**Y**	**Y**	**Y**	**Y**	
**663**	Randoll_2017 **-** The mechanism of back pain relief by spinal manipulation relies on decreased temporal summation of pain	Y	Y	Y	Y	Y	N	hand-held dynamometer
**664**	**Reneker_2017 - Feasibility of early physical therapy for dizziness after a sports-related concussion: A randomized clinical trial**	**Y**	**Y**	**Y**	**Y**	**Y**	**Y**	
**665**	Sadria_2017 **-** A comparison of the effect of the active release and muscle energy techniques on the latent trigger points of the upper trapezius	Y	Y	Y	N	Y	N	
**666**	**Sharifi Rizi_2017 - The effect of acupressure on pain, anxiety, and the physiological indexes of patients with cancer undergoing bone marrow biopsy**	**Y**	**Y**	**Y**	**Y**	**Y**	**Y**	
**667**	**Shen_2017 - The Effects of Acupressure on Meridian Energy as well as Nausea and Vomiting in Lung**	**Y**	**Y**	**Y**	**Y**	**Y**	**Y**	

	**Cancer Patients Receiving Chemotherapy**							
**668**	**Shen_2017 - The SIESTA Trial: A Randomized Study Investigating the Efficacy, Safety, and Tolerability of Acupressure versus Sham Therapy for Improving Sleep Quality in Patients with End-Stage Kidney Disease on Hemodialysis**	**Y**	**Y**	**Y**	**Y**	**Y**	**Y**	
**669**	**Silva_2017 - Effects of Anteroposterior Talus Mobilization on Range of Motion, Pain, and Functional Capacity in Participants With Subacute and Chronic Ankle Injuries: A Controlled Trial**	**Y**	**Y**	**Y**	**Y**	**Y**	**Y**	
**670**	Soltani_2017 - The effect of acupressure on uterine tone and pain after delivery	Y	Y	Y	Y	Y	N	requested via researchgate on 20.08.2019 and on 10.09.2019, no answer
**671**	Torkzahrani_2017 - The effect of acupressure on the initiation of labor: A randomized controlled trial	Y	Y	Y	Y	Y	N	same as ID540
**672**	Yang_2017 - Effectiveness and safety of Chinese massage therapy (Tui Na) on post-stroke spasticity: a prospective multicenter randomized controlled trial	Y	Y	Y	Y	Y	N	same as ID 574
**673**	Younes_2017 - Effect of spinal manipulative treatment on cardiovascular autonomic control in patients with acute low back pain	Y	Y	Y	Y	Y	N	osteopathy

**674**	**Yuen_2017 - Changes in Lower Limb Strength and Function Following Lumbar Spinal Mobilization**	**Y**	**Y**	**Y**	**Y**	**Y**	**Y**	
**675**	**Abadi_2018 - Effect of Acupressure on Preoperative Cesarean Section Anxiety**	**Y**	**Y**	**Y**	**Y**	**Y**	**Y**	
**676**	**Abbaszadeh_2018 - Effects of foot reflexology on anxiety and physiological parameters in patients undergoing coronary artery bypass graft surgery: A clinical trial**	**Y**	**Y**	**Y**	**Y**	**Y**	**Y**	
**677**	**Ahmedov_2018 - Effect of Meridian Acupressure on Aerobic Performance of Healthy Young Population: A Randomized Controlled Study**	**Y**	**Y**	**Y**	**Y**	**Y**	**Y**	
**678**	**Alvarenga_2018 - The effects of a single session of lumbar spinal manipulative therapy in terms of physical performance test symmetry in asymptomatic athletes: a single-blinded, randomised controlled study**	**Y**	**Y**	**Y**	**Y**	**Y**	**Y**	
**679**	**Araujo_2018 - Autonomic function and pressure pain threshold following thoracic mobilization in asymptomatic subjects: A randomized controlled trial**	**Y**	**Y**	**Y**	**Y**	**Y**	**Y**	
**680**	Arguisuelas Martinez_2018 - Myofascial release improves pain and disability in non-specific chronic low back pain: A randomized clinical trial	Y	Y	Y	Y	Y	N	same as ID45

**681**	Avisa_2018 - Effectiveness of Acupressure on Dental Anxiety in Children	Y	Y	Y	Y	Y	N	requested via researchgate on 20.09.2019 and on 11.11.2019, no answer
**682**	**Baarbe_2018 - Subclinical recurrent neck pain and its treatment impacts motor training-induced plasticity of the cerebellum and motor cortex**	**Y**	**Y**	**Y**	**Y**	**Y**	**Y**	
**683**	Bahr_2018 - Effects of a massage-like essential oil application procedure using Copaiba and Deep Blue oils in individuals with hand arthritis	Y	Y	Y	Y	Y	N	different oil as sham
**684**	**Bashtian_2018 - Evaluation of acupressure effects on self-efficacy and pregnancy rate in infertile women under in vitro fertilization/intracytoplasmic sperm injection treatment: A randomized controlled trial**	**Y**	**Y**	**Y**	**Y**	**Y**	**Y**	
**685**	**Bracht_2018 - Effects of cervical manipulation on pain, grip force control, and upper extremity muscle activity: a randomized controlled trial**	**Y**	**Y**	**Y**	**Y**	**Y**	**Y**	
**686**	Comino_2018 - Effects of myofascial induction on mobility and motor control in head and neck cancer survivors: A randomized, crossover, and single blind study	Y	Y	Y	Y	Y	N	Placebo therapy consisted of an unplugged electrotherapy application

**687**	**da Silva_2018 - Influence of Spinal Manipulation on Autonomic Modulation and Heart Rate in Patients With Rotator Cuff Tendinopathy.**	**Y**	**Y**	**Y**	**Y**	**Y**	**Y**	
**688**	**De Groef_2017 - Effect of myofascial techniques for treatment of upper limb dysfunctions in breast cancer survivors: randomized controlled trial**	**Y**	**Y**	**Y**	**Y**	**Y**	**Y**	
**689**	dehghanmehr_2018 - THE IMPACT OF FOOT REFLEXOLOGY MASSAGE ON ANXIETY CAUSED BY BLOOD TRANSFUSION IN CHILDREN WITH THALASSEMIA	Y	Y	Y	Y	Y	N	placebo is common massage
**690**	**Pecos-Martín_2018 - Effects of an anteroposterior mobilization of the glenohumeral joint in overhead athletes with chronic shoulder pain: A randomized controlled trial**	**Y**	**Y**	**Y**	**Y**	**Y**	**Y**	
**691**	**Farazdaghi_2018 - Effect of sacroiliac manipulation on postural sway in quiet standing: a randomized controlled trial**	**Y**	**Y**	**Y**	**Y**	**Y**	**Y**	
**692**	**Fassoulaki_2018 - Acupuncture and Acupressure Applied on the Extra-1 (Yintang) Acupoint in Healthy Volunteers Do Not Affect Regional Cerebral Blood Flow as Assessed by the Pulsatility Index: A Cohort Observational Study**	**Y**	**Y**	**Y**	**Y**	**Y**	**Y**	

**693**	**Garcia-Perez-Juana_2018 - Changes in Cervicocephalic Kinesthetic Sensibility, Widespread Pressure Pain Sensitivity, and Neck Pain After Cervical Thrust Manipulation in Patients With Chronic Mechanical Neck Pain: A Randomized Clinical Trial**	**Y**	**Y**	**Y**	**Y**	**Y**	**Y**	
**694**	**Haavik_2018 - Chiropractic spinal manipulation alters TMS induced I-wave excitability and shortens the cortical silent period**	**Y**	**Y**	**Y**	**Y**	**Y**	**Y**	
**695**	**Joo_2018 - Immediate Effects of Thoracic Spinal Manipulation on Pulmonary Function in Stroke Patients: A Preliminary Study**	**Y**	**Y**	**Y**	**Y**	**Y**	**Y**	
**696**	**Kachmar_2018 - Influence of Spinal Manipulation on Muscle Spasticity and Manual Dexterity in Participants With Cerebral Palsy: Randomized Controlled Trial**	**Y**	**Y**	**Y**	**Y**	**Y**	**Y**	
**697**	Kim_2018 - The Effects of Posterior Talar Glide and Dorsiflexion of the Ankle Plus Mobilization with Movement on Balance and Gait Function in Patient with Chronic Stroke: A Randomized Controlled Trial	Y	N	Y	Y	Y	N	intervention done with therapeutic belt
**698**	**Lascurain-Aguirrebena_2018 - Immediate effects of cervical mobilisations on global perceived**	**Y**	**Y**	**Y**	**Y**	**Y**	**Y**	

	**effect, movement associated pain and neck kinematics in patients with non-specific neck pain. A double blind placebo randomised controlled trial**							
**699**	Leonés-Macías_2018 - Effects of manual therapy on the diaphragm in asthmatic patients: A randomized pilot study	Y	Y	Y	N	Y	N	placebo is not manual therapy but ultrasound
**700**	Li_2018 - Self-Acupressure for Older Adults With Symptomatic Knee Osteoarthritis: A Randomized Controlled Trial	Y	Y	Y	Y	Y	N	wooden hand-helded device
**701**	**Lohman_2018 - The immediate effects of cervical spine manipulation on pain and biochemical markers in females with acute non-specific mechanical neck pain: a randomized clinical trial**	**Y**	**Y**	**Y**	**Y**	**Y**	**Y**	
**702**	Mansouri_2018 - A placebo-controlled clinical trial to evaluate the effectiveness of massaging on infantile colic using a random-effects joint model	Y	Y	Y	Y	Y	N	placebo is not manual therapy but ultrasound
**703**	**Marizeiro_2018 - Immediate effects of diaphragmatic myofascial release on the physical and functional outcomes in sedentary women: A randomized placebo-controlled trial**	**Y**	**Y**	**Y**	**Y**	**Y**	**Y**	

**704**	**Opavsky_2018 - The effects of sustained manual pressure stimulation according to Vojta Therapy on heart rate variability**	**Y**	**Y**	**Y**	**Y**	**Y**	**Y**	
**705**	Sayari_2018 - Effect of foot reflexology massage on physiological indices in patients with acute myocardial infarction	Y	Y	Y	Y	Y	N	full text requested to Monir Nobahar on 10.10.2019 and again on 31.10.2019, no answer
**706**	Seifert_2018 - Rhythmical massage improves autonomic nervous system function: a single-blind randomised controlled trial	Y	Y	Y	Y	Y	N	same as ID335
**707**	**Sipko_2018 - Effect of Sacroiliac Joint Mobilization on the Level of Soft Tissue Pain Threshold in Asymptomatic Women**	**Y**	**Y**	**Y**	**Y**	**Y**	**Y**	
**708**	Smith_2018 - Effect of a lateral glide mobilisation with movement of the hip on vibration threshold in healthy volunteers	Y	Y	Y	Y	Y	N	same as ID523
**709**	**Tanaka_2018 - Effect of Continuous Compression Stimulation on Pressure-Pain Threshold and Muscle Spasms in Older Adults With Knee Osteoarthritis: A Randomized Trial**	**Y**	**Y**	**Y**	**Y**	**Y**	**Y**	
**710**	Vier_2018 - Effects of spinal manipulation and pain education on pain in patients with chronic low	Y	Y	Y	Y	Y	N	protocol

	back pain: a protocol of randomized sham-controlled trial						
**711**	**Xia_2018 - Effect of Auricular Point Acupressure on Axial Neck Pain After Anterior Cervical Discectomy and Fusion: A Randomized Controlled Trial**	**Y**	**Y**	**Y**	**Y**	**Y**	**Y**

**712**	**Abbasi_2019 - Effect of acupressure on constipation in patients undergoing hemodialysis: a randomized double-blind controlled clinical trial**	**Y**	**Y**	**Y**	**Y**	**Y**	
713	Abbasi_2020 - Short-term effect of kinesiology taping on pain, functional disability and lumbar proprioception in individuals with nonspecific chronic low back pain: a double-blinded, randomized trial	Y	N	Y	N	N	kinesiology taping
714	Abdel-Aal_2020 - Effects of kinesiotaping and exercise program on patients with obesity-induced coccydynia: a randomized, double-blinded, sham-controlled clinical trial	Y	N	Y	Y	N	kinesiology taping
**715**	**Agha_2020 - Effect of Pressure on the Yinmen Point in Relief of Pain After Middle Ear Surgery: A Randomized Clinical Trial**	**Y**	**Y**	**Y**	**Y**	**Y**	
716	Ahmadizadeh_2021 - Immediate and short-term effect of quadriceps kinesio-taping on postural sway in children with spastic cerebral palsy: A before-after trial	Y	N	Y	N	N	kinesio-taping
717	Ajeng_2020 - The effect back massage to the height of uterine fundus in primiparous normal postpartum mothers	?	?	?	?	N	not found

718	Ajimsha_2021 - Effectiveness of external myofascial mobilisation in the management of male chronic pelvic pain of muscle spastic type: A retrospective study	N	Y	N	N	N	
719	Akbaba_2019 - The effectiveness of trigger point treatment in rotator cuff pathology: A randomized controlled double-blind study	Y	Y	Y	N	N	no placebo/sham group
**720**	**Akbarnezhad_2019 - The effect of acupressure therapy on pain, stiffness and physical functioning of knees among older adults diagnosed with osteoarthritis: A pilot randomized control trial**	**Y**	**Y**	**Y**	**Y**	**Y**	
**721**	**Akgun_2020 - The effects of acupressure on post-cesarean pain and analgesic consumption: a randomized single-blinded placebo-controlled study**	**Y**	**Y**	**Y**	**Y**	**Y**	
722	Akhtar_2020 - The effectiveness of routine physiotherapy with and without neuromobilization on pain and functional disability in patients with shoulder impingement syndrome; a randomized control clinical trial	Y	Y	Y	N	N	
723	Akpinar_2021 - Investigation of effectiveness of two different Kinesiotaping techniques in myofascial pain syndrome: an open-label randomized clinical trial	Y	N	Y	N	N	
724	Aksoy_2020 - The immediate effect of neurodynamic techniques on jumping performance: A randomised double-blind study	Y	Y	Y	N	N	
725	Alameri_2020 - Efficacy of Precise Foot Massage Therapy on Pain and Anxiety Following Cardiac Surgery: Pilot Study	Y	Y	Y	Y	N	cream

726	Albin_2019 - Short-term Effects of Manual Therapy in Patients After Surgical Fixation of Ankle and/or Hindfoot Fracture: A Randomized Clinical Trial	Y	Y	Y	Y	Y	
727	Ali_2021 - Ischemic compression technique versus myofascial release of upper trapezius muscle in mechanical neck pain in females of jouf university	Y	Y	Y	N	N	compression technique vs myofascial release
728	Ali_2021 - The role of multi-slice computed tomography for the assessment of bronchiectasis	Y	N	Y	N	N	
729	Alimoradi_2020 - Effects of ear and body acupressure on labor pain and duration of labor active phase: A randomized controlled trial	Y	Y	Y	N	N	no placebo/sham therapy
730	Alimoradi_2019 - Comparing the effect of auricular acupressure and body acupressure on pain and duration of the first stage of labor: Study protocol for a randomized controlled trial	Y	Y	Y	N	N	no placebo/sham therapy
**731**	**Alinaghizadeh_2021 - Effect of Persian acupressure (Ghamz) on Patients with Knee Osteoarthritis: A Single-Blinded Parallel Clinical Trial**	**Y**	**Y**	**Y**	**Y**	**Y**	
732	Alkhadhrawi_2019 - Effects of myofascial trigger point dry cupping on pain and function in patients with plantar heel pain: A randomized controlled trial	Y	Y	Y	N	N	no placebo/sham therapy
**733**	**Alkhawajah_2019 - The effect of mobilization with movement on pain and function in patients with knee osteoarthritis: A randomized double-blind controlled trial**	**Y**	**Y**	**Y**	**Y**	**Y**	

**734**	**Allahbakhhsian_2020 - The effects of foot reflexology on agitation and extubation time in male patients following coronary artery bypass surgery: A randomized controlled clinical trial**	**Y**	**Y**	**Y**	**Y**	**Y**	
735	Alshami_2021 - Effect of Neural Mobilization Exercises in Patients With Low Back-Related Leg Pain With Peripheral Nerve Sensitization: A Prospective, Controlled Trial	Y	Y	Y	N	N	no placebo/sham
736	Altas_2020 - The effect of physical therapy and rehabilitation modalities on sleep quality in patients with primary knee osteoarthritis: A single-blind, prospective, randomized-controlled study	Y	Y	Y	N	N	no placebo/sham
737	Alvarez_2019 - Effects of Massage Therapy and Kinesitherapy to Develop Hospitalized Preterm Infant's Anthropometry: A Quasi-Experimental Study	Y	Y	Y	N	N	control group received regular medical and nursing care.
738	Aminian-Far_2021 - Kinesio taping as an alternative treatment for manual laborers with carpal tunnel syndrome: A double-blind randomized clinical trial	Y	N	Y	N	N	kinesio-taping
**739**	**An_2021 - Effects of Cervical Spine Mobilization on Respiratory Function and Cervical Angles of Stroke Patients: A Pilot Study**	**Y**	**Y**	**Y**	**Y**	**Y**	
**740**	**Araujo_2019 - Short-Term Effects of Different Rates of Thoracic Mobilization on Pressure Pain Thresholds in Asymptomatic Individuals: A Randomized Crossover Trial**	**Y**	**Y**	**Y**	**Y**	**Y**	
741	Arul_2019 - Effectiveness of myofascial release technique versus positional release technique on myofascial pain syndrome	Y	Y	Y	N	N	no placebo/sham

**742**	**Asgari_2020 - The effects of acupressure on the symptoms severity and function status and electrodiagnostic findings in patients with carpal tunnel syndrome**	**Y**	**Y**	**Y**	**Y**	**Y**	**SeaBands were used for each patient**
743	Asgari_2019 - Comparing acupressure with aromatherapy using Citrus aurantium in terms of their effectiveness in sleep quality in patients undergoing percutaneous coronary interventions: A randomized clinical trial	Y	Y	Y	Y	N	SeaBands were used for each patient
744	Asgari_2020 - The effects of acupressure on the symptoms severity and function status and electrodiagnostic findings in patients with carpal tunnel syndrome	Y	Y	Y	Y	N	same ad ID742
745	Aspinall_2019 - The effects of acupressure on the symptoms severity and function status and electrodiagnostic findings in patients with carpal tunnel syndrome	Y	Y	Y	Y	N	same ad ID742
746	Aspinall_2020 - Changes in pressure pain threshold and temporal summation in rapid responders and non-rapid responders after lumbar spinal manipulation and sham: A secondary analysis in adults with low back pain	Y	Y	Y	N	N	
747	Avdeeva_2019 - Risk factors that cause development and progression of degenerative and dystrophic diseases in the spinal column as per results obtained during screening tests on people living in Saint Petersburg	N	N	N	N	N	
748	Awad_2020 - Effect of acupressure on prostaglandin f2a in primary dysmenorrhea: A randomized controlled trial	?	?	?	?	N	not found
749	Aziz_2021 - Effect of muscle energy technique versus aerobic exercise on chronic cyclic pelvic pain	Y	Y	Y	N	N	no placebo/sham

750	Baeske_2020 - The inclusion of mobilisation with movement to a standard exercise programme for patients with rotator cuff related pain: a randomised, placebo-controlled protocol trial	Y	Y	Y	Y	N	Incomplete
751	Bang_2020 - Effect of auricular acupressure on sleep and pruritus in patients undergoing hemodialysis	Y	Y	Y	N	N	
752	Barghamadi_2020 - The effect of ear acupressure (auriculotherapy) on sexual function of lactating women: protocol of a randomized sham controlled trial	Y	N	Y	Y	N	not manual - vaccaria seeds
**753**	**Basiri_2020 - Effects of foot reflexology on neonatal jaundice: A randomized sham-controlled trial**	**Y**	**Y**	**Y**	**Y**	**Y**	
754	Baumbach_2019 - A pilot study of exercise-induced changes in mitochondrial oxygen metabolism measured by a cellular oxygen metabolism monitor (PICOMET)	N	N	N	N	N	
755	Baumgartner_2021 - How Are Discrepant Parent-Child Reports Integrated? A Case of Depressed Adolescents	N	N	N	N	N	
756	Baute_2019 - Complementary and Alternative Medicine for Painful Peripheral Neuropathy	N	N	N	N	N	
757	Bautrant_2019 - Provoked vulvar vestibulodynia: Epidemiology in Europe, physio-pathology, consensus for first-line treatment and evaluation of second-line treatments	Y	N	Y	N	N	
758	Bavaresco_2019 - Carbohydrate mouth rinse improves cycling performance carried out until the volitional exhaustion	Y	N	Y	N	N	

759	Bavalier_2019 - Enhancing Attentional Control: Lessons from Action Video Games	N	N	N	N	N	
760	Baxter_2021 - Perceptions and experiences of a manual therapy trial: a qualitative study of people with moderate to severe COPD	N	N	N	N	N	
761	Bayani_2021 - Effect of probiotics on enteral milk tolerance and prevention of necrotizing enterocolitis in preterm neonates	Y	N	Y	N	N	
762	Bayartai_2020 - Genetic and environmental effects on lumbar posture, flexibility and motion control in healthy adults	Y	N	N	N	N	
**763**	**Bender_2019 - Massage therapy slightly decreased pain intensity after habitual running, but had no effect on fatigue, mood or physical performance: a randomised trial**	**Y**	**Y**	**Y**	**Y**	**Y**	
**764**	**Bernal-Utrera_2020 - Manual therapy versus therapeutic exercise in non-specific chronic neck pain: A randomized controlled trial**	**Y**	**Y**	**Y**	**Y**	**Y**	
**765**	**Baghat_2020 - Immediate effects of Mulligan's techniques on pain and functional mobility in individuals with knee osteoarthritis: a randomized control trial**	**Y**	**Y**	**Y**	**Y**	**Y**	
766	Bicer_2021 - The effect of acupressure on blood pressure level and pulse rate in individuals with essential hypertension: A randomized controlled trial	?	?	?	?	N	not found
767	Bicer_2021 - The Effect of Body Acupressure on Blood Pressure and Fatigue Levels in Individuals Suffering From Hypotension During Hemodialysis: A Randomized Controlled Trial	Y	N	Y	N	N	electrostimulation device

768	Blanco-Diaz_2020 - Manual Physical Therapy in the Treatment of Functional Constipation in Children: A Pilot Randomized Controlled Trial	Y	Y	Y	N	N	no sham group
769	Boff_2020 - Effectiveness of spinal manipulation and myofascial release compared with spinal manipulation alone on health-related outcomes in individuals with non-specific low back pain: randomized controlled trial	Y	Y	Y	N	N	no sham group
770	Bomi_2020 - The effects of auricular acupressure on joint pain and shoulder range of motion in older adults	?	?	?	?	N	not found
**771**	**Bond_2020 - Effect of spinal manipulative therapy on mechanical pain sensitivity in patients with chronic nonspecific low back pain: a pilot randomized, controlled trial**	**Y**	**Y**	**Y**	**Y**	**Y**	
772	Bondi_2021 - Effects of Reiki on Pain and Anxiety in Women Hospitalized for Obstetrical- and Gynecological-Related Conditions	Y	Y	N	N	N	
**773**	**Borji_2021 - Effectiveness of acupressure on anxiety of children undergoing anesthesia**	**Y**	**Y**	**Y**	**Y**	**Y**	
774	Bostrom_2019 - Clinical comparative effectiveness of acupuncture versus manual therapy treatment of lateral epicondylitis: feasibility randomized clinical trial	Y	Y	Y	N	N	no sham group
775	Bourque_2019 - Combining physical therapy and cognitive behavioral therapy techniques to improve balance confidence and community participation in people with unilateral transtibial amputation who use lower limb prostheses: a study protocol for a randomized sham-control clinical trial	Y	N	Y	N	N	cognitive behavioral-physical therapy (CBPT) intervention

776	Brandl_2021 - Immediate Effects of Myofascial Release on the Thoracolumbar Fascia and Osteopathic Treatment for Acute Low Back Pain on Spine Shape Parameters: A Randomized, Placebo-Controlled Trial	Y	Y	Y	Y	N	osteopathy
777	Bruck_2021 - Fascial treatment versus manual therapy (HVLA) in patients with chronic neck pain: A randomized controlled trial	Y	Y	Y	N	N	comparison
778	Brurberg_2019 - Manipulation techniques for infant torticollis	N	Y	Y	Y	N	review
779	Buran_2021 - Effect of Sustained Natural Apophyseal Glides on Stiffness of Lumbar Stabilizer Muscles in Patients With Nonspecific Low Back Pain: Randomized Controlled Trial	Y	Y	Y	Y	N	request 08-02-2022 and 25-02-2022, no response
780	Calixtre_2019 - Effectiveness of mobilisation of the upper cervical region and craniocervical flexor training on orofacial pain, mandibular function and headache in women with TMD. A randomised, controlled trial	Y	Y	Y	N	N	controul group without manual sham
781	Calixtre_2021 - Effects of myofascial release applied to neck muscles and craniocervical flexor training in patients with chronic myofascial TMD: A single arm study	Y	Y	Y	N	N	comparison
782	Caliskan_2021 - Effect of therapeutic touch on sleep quality and anxiety in individuals with chronic obstructive pulmonary disease: A randomized controlled trial	Y	Y	Y	N	N	no info about "control group"
783	Capos de Almeida_2021 - Acute effect of different duration times of application of myofascial release on quadriceps femoris strength: A randomized clinical trial	Y	Y	Y	N	N	placebo = manual contact with gel

784	Carpino_2020 - Does manual therapy affect functional and biomechanical outcomes of a sit-to-stand task in a population with low back pain? A preliminary analysis	N	Y	N	N	N	no control
785	Carralero-Martinez_2020 - Effect of myofascial induction techniques on pain reduction in patients with myofascial chronic pelvic pain	\	\	\	\	N	not found
786	Carrasco-Martinez_2020 - Short-term effectiveness of the flexion-distraction technique in comparison with high-velocity vertebral manipulation in patients suffering from low-back pain	Y	Y	N	N	N	no control
787	Carroll_2021 - The effects of calf massage in boys with Duchenne muscular dystrophy: a prospective interventional study	Y	Y	N	N	N	control rest period (placebo)
788	Castejon-castejon_2019 - Effectiveness of craniosacral therapy in the treatment of infantile colic. A randomized controlled trial	Y	Y	N	N	N	no treatment
789	Castro-Martin_2020 - Effects of a single myofascial induction session on neural mechanosensitivity in breast cancer survivors: a secondary analysis of a crossover study [with consumer summary]	Y	Y	N	N	N	unplugged pulsed 30 minutes of shortwave therapy
790	Castro-Martin_2021 - Myofascial induction therapy improves the sequelae of medical treatment in head and neck cancer survivors: A single-blind, placebo-controlled, randomized cross-over study	Y	Y	N	N	N	unplugged pulsed 30 minutes of shortwave therapy
791	Cecen_2021 - The effect of hand and foot massage on fatigue in hemodialysis patients: A randomized controlled trial	Y	Y	N	N	N	no sham
792	Celenay_2019 - Adding connective tissue manipulation to physiotherapy for chronic low back pain improves pain, mobility, and well-being: a randomized controlled trial	Y	Y	N	N	N	no sham

**793**	**Celik_2019 - The effect of acupressure at the sanyinjiao point on the labor pain relief and duration of labor in Turkish nulliparous women**	**Y**	**Y**	**Y**	**Y**	**Y**	
794	Cen_2021 - Therapeutic effects of auricular point acupressure on the recovery of patients after pterygium surgery: A pilot study	Y	N	Y	N	N	without Cowherb seeds
795	Ceniza-Bordallo_2019 - The placebo effect in the treatment of musculoskeletal neck pain: A randomized clinical trial	Y	N	Y	N	N	taping
796	Ceprnja_2019 - Does muscle energy technique have an immediate benefit for women with pregnancy-related pelvic girdle pain?	Y	N	Y	N	N	sham transcutaneous electrical nerve stimulation
797	Ceyla_2019 - The effect of acupressure on cardiac rhythm and heart rate among patients with atrial fibrillation: the relationship between heart rate and fatigue	Y	N	Y	N	N	specially developed acupressure device
798	Cha_2019 - Effects of auricular acupressure on obesity in adolescents	Y	N	Y	N	N	vaccaria seeds
799	Colombo_2019 - The effects induced by spinal manipulative therapy on the immune and endocrine systems	N	Y	Y	Y	N	review
800	Comachio_2020 - Effectiveness of Manual and Electrical Acupuncture for Chronic Non-specific Low Back Pain: A Randomized Controlled Trial	Y	N	Y	N	N	electroacupuncture
801	Cook_2021 - Chiropractic Management of a Symptomatic Patient Who Previously Had Surgery for Cauda Equina Syndrome	N	Y	N	N	N	case report

802	Creighton_2020 - Fascial Manipulation method as a treatment for pain, atrophy and skin depigmentation after pes anserine bursa corticosteroid injection: A case report	N	Y	N	N	N	case report
803	Dagar_2020 - Effects of spinal immobilization at 20° on end-tidal carbon dioxide	Y	Y	N	N	N	no sham
804	Dalgleish_2021 - Occipitoatlantal decompression and noninvasive vagus nerve stimulation slow conduction velocity through the atrioventricular node in healthy participants	Y	Y	Y	N	N	ime control group (CTR) consisted of 15 min of rest.
805	Davodabady_2020 - Randomized controlled trial of the foot reflexology on pain and anxiety severity during dressing change in burn patients	Y	Y	Y	N	N	no sham.
806	De Marco_2021 - Effects of visceral manipulation associated with pelvic floor muscles training in women with urinary incontinence: A randomized controlled trial	Y	Y	Y	Y	N	not found
807	Dellovo_2019 - Effects of auriculotherapy and midazolam for anxiety control in patients submitted to third molar extraction	Y	Y	Y	Y	N	seed
808	Deng_2019 - Self-massage with Enzyme-rich Ginger Oil in the Treatment of Primary Dysmenorrhea:a Randomized,Double-blind,Controlled Clinical Trial	Y	Y	Y	N	N	the difference is the oil
809	Deniz_2021 - A Randomized Controlled Trial: The Effect of Acupressure and Foot Reflexology on Pain During Heel-Lancing in Neonates	Y	Y	Y	Y	N	request 09-02-2022 and 26-02-2022, no response

810	Derya_2021 - The Effect of Acupressure on Daytime Sleepiness and Sleep Quality in Hemodialysis Patients	Y	Y	Y	N	N	No intervention was applied to the control group
811	Diaz-Pulido_2021 - Efficacy of manual therapy and transcutaneous electrical nerve stimulation in cervical mobility and endurance in subacute and chronic neck pain: A randomized clinical trial	Y	Y	Y	N	N	MT versus TENS
812	Didehdar_2020 - The effect of spinal manipulation on brain neurometabolites in chronic nonspecific low back pain patients: a randomized clinical trial	Y	Y	Y	N	N	no sham.
813	Dogan_2021 - Effects of reflexology on pain, fatigue, and quality of life in multiple sclerosis patients: A clinical study	Y	Y	Y	N	N	no sham
814	Doner_2021 - Effect of massage therapy with lavender oil on severity of restless legs syndrome and quality of life in hemodialysis patients	Y	Y	Y	N	N	the difference is the oil
815	dos Santos_2021 - The effects of functional fascial taping (FFT) associated with conventional physiotherapy on the knee functionality: A quasi-experimental study	Y	Y	Y	N	N	placebo taping
816	Dunning_2021 - Spinal manipulation and electrical dry needling in patients with subacromial pain syndrome: A multicenter randomized clinical trial	Y	N	Y	N	N	no sham
817	Dunning_2021 - Spinal manipulation and perineural electrical dry needling in patients with cervicogenic headache: a multicenter randomized clinical trial	Y	N	Y	N	N	no sham
818	Efe Arslan_2019 - The Effect of Aromatherapy Massage on Knee Pain and Functional Status in Participants with Osteoarthritis	Y	N	Y	N	N	no sham

819	El-shamy_2019 - Ameliorative potential of acupressure on gestational diabetes mellitus: A randomized controlled trial	Y	N	Y	N	N	no sham
820	El-shamy_2020 - Ameliorative potential of black sand therapy on carpal tunnel syndrome during pregnancy: A case report	N	Y	N	N	N	case report
821	Elnagger_2019 - Prospective effects of manual diaphragmatic release and thoracic lymphatic pumping in childhood asthma	Y	N	Y	N	N	no sham
822	Emamverdi_2019 - Comparing the effects of reflexology massage and acupressure on the quality of sleep in hemodialysis patients: A randomized clinical trial	Y	N	Y	N	N	no sham
823	Endamli_2019 - Investigation of fascial treatment effectiveness on pain, flexibility, functional level, and kinesiophobia in patients with chronic low back pain	Y	N	Y	N	N	comparison
824	Espi-Lopez_2020 - The beneficial effects of therapeutic craniofacial massage on quality of life, mental health and menopausal symptoms and body image: A randomized controlled clinical trial	Y	N	Y	N	N	no sham
825	Espi-Lopes_2020 - Comparison Between Classic and Light Touch Massage on Psychological and Physical Functional Variables in Athletes: a Randomized Pilot Trial	Y	N	Y	N	N	comparison
826	Espinoza Diaz_2021 - Relationship between dysmenorrhoea and Myofascial Pain Syndrome. A physiotherapist perspective. Pilot randomized trial	Y	N	Y	N	N	no sham
827	Essawy_2021 - Comparing the effect of acupressure and ginger on chemotherapy gastrointestinal side-effects in children with leukemia	Y	N	Y	N	N	no sham

828	Estes_2021 - Combined Transcutaneous Spinal Stimulation and Locomotor Training to Improve Walking Function and Reduce Spasticity in Subacute Spinal Cord Injury: A Randomized Study of Clinical Feasibility and Efficacy	Y	N	Y	N	N	no sham
829	Fagundes_2020 - Immediate effects of a lumbar spine manipulation on pain sensitivity and postural control in individuals with nonspecific low back pain: a randomized controlled trial	Y	Y	Y	Y	N	osteopathy
830	Fahmy_2021 - Deep cervical flexor pressure biofeedback exercise versus integrated neuromuscular inhibition technique in chronic mechanical neck pain: A randomized controlled trial	Y	N	Y	N	N	no sham
831	Fami_2020 - Evaluation of scapular mobilization and comparison to pectoralis minor stretching in individuals with rounded shoulder posture: A randomized controlled trial	Y	N	Y	N	N	control group received no treatment
832	Fetai_2021 - Self-massage and low-level laser in treatment of Masseter Myalgia: Short-term effect	Y	N	Y	N	N	no sham
**833**	**Fisher_2020 - Short-term effects of thoracic spine thrust manipulation, exercise, and education in individuals with low back pain: a randomized controlled trial**	**Y**	**Y**	**Y**	**Y**	**Y**	
834	Folli_2021 - A single session with a roller massager improves hamstring flexibility in healthy athletes: a randomized placebo-controlled crossover study	Y	N	Y	N	N	no manual
**835**	**Fosberg_2020 - The effects of thrust joint manipulation on the resting and contraction thickness of transversus abdominis in patients with low back pain: a randomized control trial [with consumer summary]**	**Y**	**Y**	**Y**	**Y**	**Y**	

836	Fouda_2021 - Effects of proprioceptive neuromuscular facilitation techniques in treating chronic nonspecific low back pain patients	Y	N	Y	N	N	no sham
**837**	**Fraser_2020 - Effects of midfoot joint mobilization on ankle-foot morphology and function following acute ankle sprain. A crossover clinical trial**	**Y**	**Y**	**Y**	**Y**	**Y**	
838	Freire_2021 - Effects of myofascial release of the ankle plantar flexors on static postural balance of young men: A randomized clinical trial	Y	Y	Y	N	N	roller massager
839	Fritz_2021 - Optimization of Spinal Manipulative Therapy Protocols: A Factorial Randomized Trial Within a Multiphase Optimization Framework	Y	N	Y	N	N	no sham
840	Frontczak_2020 - Impact of physiotherapeutic methods on urinary incontinence in the elderly	N	Y	Y	Y	N	review
841	Fu_2021 - Auricular acupressure for adverse events following immunization related to COVID-19 vaccine injection: study protocol for a multicenter, three-arm, blinded randomized controlled trial	N	N	N	N	N	
842	Fu_2021 - Traditional Chinese medicine auricular point acupressure for the relief of pain, fatigue, and gastrointestinal adverse reactions after the injection of novel coronavirus-19 vaccines: a structured summary of a study protocol for a multicentre, three-arm, single-blind, prospective randomized controlled trial	N	N	N	N	N	
843	Fu_2021 - Auricular acupressure for adverse events following immunization related to COVID-19 vaccine injection: study protocol for a multicenter, three-arm, blinded randomized controlled trial	N	N	N	N	N	

844	Fujita_2019 - How effective is physical therapy for gait muscle activity in hemiparetic patients who receive botulinum toxin injections?	Y	N		N	N	
845	Fung_2021 - The clinical effects of mobilization with passive ankle dorsiflexion using a passive ankle dorsiflexion apparatus on older patients with knee osteoarthritis: A randomized trial	Y	Y	Y	N	N	
846	Fuzari_2019 - Whole body vibration improves maximum voluntary isometric contraction of knee extensors in patients with chronic kidney disease: A randomized controlled trial	Y	N	N	N	N	
847	Galaasen_2021 - The effect of spinal manipulative therapy and home stretching exercises on heart rate variability in patients with persistent or recurrent neck pain: a randomized controlled trial	Y	Y	Y	N	N	
848	Gamelas_2019 - Neural gliding versus neural tensioning: Effects on heat and cold thresholds, pain thresholds and hand grip strength in asymptomatic individuals	Y	N	N	N	N	
849	Gan_2019 - The effect of self- myofascial release on neuromuscular responses of lumber in healthy subjects during trunk flexion-extension	?	?	?	?	N	not found
850	Ganesh_2021 - Physiotherapist management of a patient with spastic perineal syndrome and subsequent constipation: a case report	N	N	N	N	N	
851	Gao_2020 - The effect of auricular therapy on blood pressure: A systematic review and meta-analysis	N	N	N	N	N	
852	Gausel_2019 - Adding Chiropractic Treatment to Individual Rehabilitation for Persistent Pelvic Girdle Pain 3 to 6 Months After Delivery: A Pilot Randomized Trial	Y	N	N	N	N	

853	Gentile_2021 - Pain improvement after healing touch and massage in breast cancer: An observational retrospective study	Y	Y	N	N	N	
**854**	**Ghaderi_2021 - The effect of emotional freedom technique on fatigue among women with multiple sclerosis: A randomized controlled trial**	**Y**	**Y**	**Y**	**Y**	**Y**	
**855**	**Ghan_2021 - Immediate Effect of Cervico-thoracic Mobilization on Deep Neck Flexors Strength in Individuals with Forward Head Posture: A Randomized Controlled Trial**	**Y**	**Y**	**Y**	**Y**	**Y**	
856	Ghasabmahleh_2021 - Spinal Manipulation for Subacute and Chronic Lumbar Radiculopathy: A Randomized Controlled Trial	Y	Y	N	N	N	
**857**	**Ghasemi_2021 - Aromatherapy Massage vs. Foot Reflexology on the Severity of Restless Legs Syndrome in Female Patients Undergoing Hemodialysis**	**Y**	**Y**	**Y**	**Y**	**Y**	
**858**	**Ghillodia_2020 - Effect of visceral manipulation on pain, mobility and functional disability in subjects with right shoulder adhesive capsulitis**	**Y**	**Y**	**Y**	**Y**	**Y**	
859	Ghodrati_2019 - Effect of auriculotherapy on infertile depression in women: A randomized clinical trial	Y	N	N	N	N	
860	Go_2020 - Effects of Auricular Acupressure on Women with Irritable Bowel Syndrome	Y	N	N	N	N	
**861**	**Gogate_2021 - The effectiveness of mobilization with movement on pain, balance and function following acute and sub acute inversion ankle sprain - A randomized, placebo controlled trial**	**Y**	**Y**	**Y**	**Y**	**Y**	

862	Gol_2020 - Effect of massage therapy with and without elastic bandaging on pain, edema, and shoulder dysfunction after modified radical mastectomy: A clinical trial	Y	Y	Y	N	N	
**863**	**Gonzalez_2021 - Validation of a sham novel neural mobilization technique in patients with non-specific low back pain: A randomized, placebo-controlled trial**	**Y**	**Y**	**Y**	**Y**	**Y**	
864	Gopalswami_2019 - Effect of manual therapy over conventional treatment among chronic degenerative joint disease of the knee - A prospective comparative study	Y	Y	Y	N	N	
865	Goroszeniuk_2019 - The Effect of Peripheral Neuromodulation on Pain From the Sacroiliac Joint: A Retrospective Cohort Study	Y	N	N	N	N	
866	Grande-Alonso_2019 - Physiotherapy Based on a Biobehavioral Approach with or without Orthopedic Manual Physical Therapy in the Treatment of Nonspecific Chronic Low Back Pain: A Randomized Controlled Trial	Y	N	N	N	N	
867	Grbovic_2019 - The effects of the physical procedures in patients with diabetic neuropathy	Y	N	N	N	N	
868	Griffiths_2019 - Immediate effects and associations between interoceptive accuracy and range of motion after a HVLA thrust on the thoracolumbar junction: A randomised controlled trial	Y	N	N	N	N	osteopathy
869	Griswold_2019 - A randomized clinical trial comparing non-thrust manipulation with segmental and distal dry needling on pain, disability, and rate of recovery for patients with non-specific low back pain	Y	N	N	N	N	

870	Guan_2019 - Effects of cervical rotatory manipulation on internal carotid artery in hemodynamics using an animal model of carotid atherosclerosis: A safety study	N	N	N	N	N	
871	Gul_2020 - Effects of acupressure on preoperative acute anxiety in cesarean section under spinal anesthesia	Y	N	N	N	N	
872	Han_2021 - Auricular acupressure for myopia prevention and control in children and its effect on choroid and retina: a randomized controlled trial protocol	Y	N	N	N	N	
873	Harper_2019 - Fascial manipulation vs. standard physical therapy practice for low back pain diagnoses: A pragmatic study	N	N	N	N	N	
**874**	**Hasuo_2021 - Effect of ischemic compression performed by family caregivers on myofascial pain syndrome and the care burden of the families of patients: a multicenter open-label randomized comparative study**	**Y**	**Y**	**Y**	**Y**	**Y**	
875	Hawk_2021 - The Role of Chiropractic Care in Providing Health Promotion and Clinical Preventive Services for Adult Patients with Musculoskeletal Pain: A Clinical Practice Guideline	N	N	N	N	N	
876	Hawk_2020 - Best practices for chiropractic management of patients with chronic musculoskeletal pain: A clinical practice guideline	N	N	N	N	N	
877	Ho_2020 - Effectiveness of acupoint pressure on older people with constipation in nursing homes: a double-blind quasi-experimental study	Y	N	N	N	N	

**878**	**Hoang_2021 - Pilot randomized sham-controlled trial of self-acupressure to manage the symptom cluster of insomnia, depression, and anxiety in cancer patients undergoing chemotherapy**	**Y**	**Y**	**Y**	**Y**	**Y**	
879	Hoang_2021 - Correction to: Pilot randomized sham-controlled trial of self-acupressure to manage the symptom cluster of insomnia, depression, and anxiety in cancer patients undergoing chemotherapy	N	N	N	N	N	correction
880	Hohl_2019 - The Effect of Single-Dose Massage Session on Autonomic Activity, Mood, and Affective Responses in Major Depressive Disorder	Y	Y	Y	Y	N	no manual placebo
**881**	**Holt_2019 - The effects of a single session of chiropractic care on strength, cortical drive, and spinal excitability in stroke patients**	**Y**	**Y**	**Y**	**Y**	**Y**	
**882**	**Holt_2021 - The Effects of 4 Weeks of Chiropractic Spinal Adjustments on Motor Function in People with Stroke: A Randomized Controlled Trial**	**Y**	**Y**	**Y**	**Y**	**Y**	
**883**	**Honorè_2020 - What is the effect of spinal manipulation on the pressure pain threshold in young, asymptomatic subjects? A randomized placebo-controlled trial, with a cross-over design**	**Y**	**Y**	**Y**	**Y**	**Y**	
**884**	**Hsieh_2019 - Efficacy of acupressure to prevent adverse reactions to anti-tuberculosis drugs: Randomized controlled trials**	**Y**	**Y**	**Y**	**Y**	**Y**	
885	Hsu_2021 - The effectiveness of acupressure for managing postoperative pain in patients with thoracoscopic surgery: A randomized control trail.	Y	N	N	N	N	

886	Hu_2019 - Acupressure and Therapeutic Touch in Childhood Cancer to Promote Subjective and Intersubjective Experiences of Well-being During Curative Treatment	N	N	N	N	N	
887	Hu_2021 - Self-administered acupressure for chronic severe functional constipation: A study protocol for a randomized controlled trial	Y	Y	Y	Y	Y	protocol
888	Houng_2021 - Pilot randomized sham-controlled trial of self-acupressure to manage the symptom cluster of insomnia, depression, and anxiety in cancer patients undergoing chemotherapy	Y	N	N	N	N	doubled
889	**Hussein_2021 - The immediate effect of sustained natural apophyseal glide on postural stability and pain in individuals presenting with flexion-dominant chronic low back pain: A randomized single-blinded placebo-controlled trial**	**Y**	**Y**	**Y**	**Y**	**Y**	
890	Ibrahim_2021 - The effectiveness of tensioning neural mobilization of brachial plexus in patients with chronic cervical radiculopathy: A randomized clinical trial	Y	N	N	N	N	
**891**	**Inkaya_2020 - Effect of reflexology on the constipation status of elderly people**	**Y**	**Y**	**Y**	**Y**	**Y**	
892	Izgu_2019 - Effect of Aromatherapy Massage on Chemotherapy-Induced Peripheral Neuropathic Pain and Fatigue in Patients Receiving Oxaliplatin: An Open Label Quasi-Randomized Controlled Pilot Study	Y	N	N	N	N	
893	Jeanbart_2021 - Mobilization of the neurodynamic system using proprioceptive neuromuscular facilitation decreases pain and increases mobility in lower extremities and Spine-A case report	N	N	N	N	N	

894	Jordan_2020 - Effects of a 4-week instrumented soft tissue mobilization and sham treatment as rehabilitation strategies for chronic ankle instability	Y	N	Y	Y	N	non manual intervention/placebo
895	Jung_2021 - Efficacy and safety of auricular acupressure for chemotherapy-induced peripheral neuropathy among patients with breast cancer: a study protocol for a randomized controlled trial	Y	Y	Y	Y	N	protocol
896	Juyon_2021 - The effects of auricular acupressure on the sleep of the elderly using polysomnography, actigraphy and blood test: Randomized, single-blind, sham control	Y	Y	Y	Y	N	full-text requested 05/02 and 26/02. No response
897	Kang_2021 - Effects of the suboccipital muscle inhibition technique on the range of motion of the ankle joint and balance according to its application duration: A randomized controlled trial	Y	Y	Y	Y	Y	
898	Kapikiran_2021 - The effect of foot reflexology on pain, comfort and beta endorphin levels in patients with liver transplantation: A randomized control trial	Y	N	N	N	N	
899	Karatas_2021 - Is foot reflexology effective in reducing colic symptoms in infants: A randomized placebo-controlled trial	Y	Y	Y	Y	Y	
900	Karjalian_2020 - The Effect of Acupressure on the Severity of Pruritus and Laboratory Parameters in Patients Undergoing Hemodialysis: A Randomized Clinical Trial	Y	Y	Y	Y	Y	
901	Kawi_2021 - Auricular Point Acupressure Smartphone Application to Manage Chronic Musculoskeletal Pain: A Longitudinal, One-Group, Open Pilot Trial	N	N	N	N	N	

902	Kayiran_2021 - The effectiveness of neural mobilization in addition to conservative physiotherapy on cervical posture, pain and functionality in patients with cervical disc herniation	Y	N	N	N	N	
903	Kemmler_2021 - Changes in body composition and cardiometabolic health after detraining in older men with osteosarcopenia: 6-month follow-up of the randomized controlled franconian osteopenia and sarcopenia trial (frost) study	Y	N	N	N	N	
904	Kerautret_2021 - Evaluating the effects of embedded self-massage practice on strength performance: A randomized crossover pilot trial	Y	N	N	N	N	
905	Kerautret_2021 - Effects of self-myofascial release interventions with or without sliding pressures on skin temperature, range of motion and perceived well-being: a randomized control pilot trial	Y	N	N	N	N	
**906**	**Keskin_2021 - The effect of acupressure applied to individuals receiving hemodialysis treatment on severity of thirst and quality of life**	**Y**	**N**	**Y**	**Y**	**Y**	
907	Khan_2021 - The role of myofascial release in treating patients with tension-type headaches: A critically appraised topic	?	?	?	?	?	full-text requested 05/2 and 27/02. No response
**908**	**Khanghah_2021 - Effects of Acupressure on Fatigue in Patients with Cancer Who Underwent Chemotherapy**	**RCT**	**Y**	**Y**	**Y**	**Y**	
909	Khorsand_2019 - The effect of massage and topical violet oil on the severity of pruritus and dry skin in hemodialysis patients: A randomized controlled trial	RCT	Y	Y	Y	N	different oils

910	Kim_2021 - Effects of acupressure on pain, flexibility, and substance p in middle-age women with chronic neck pain	RCT	Y	Y	N	N	
911	Kim_2021 - The Effect of Auricular Acupressure for Chronic Low Back Pain in Elders: A Randomized Controlled Study	RCT	Y	Y	Y	N	full-text requested 05/2 and 28/02. No response
912	Kim_2020 - Effects of Hand Acupressure on Sleep Quality and Pruritus in Patients on Hemodialysis	RCT	Y	Y	Y	N	not found
913	Kilic_2021 - Aromatherapy massage for pain and xerosis after repeated needle insertion into a fistula arm in hemodialysis	RCT	Y	Y	Y	N	difference is the oil
914	Kostadinovic_2020 - Efficacy of the lumbar stabilization and thoracic mobilization exercise program on pain intensity and functional disability reduction in chronic low back pain patients with lumbar radiculopathy: A randomized controlled trial	RCT	N	Y	N	N	exercise, not MT
915	Kovanur_2021 - Thoracic spinal manipulation effect on neuroendocrine response in people with Achilles tendinopathy: a randomized crossover trial [with consumer summary]	RCT	Y	Y	N	N	no manual
916	Kul_2019 - Comparison of the efficacy of conventional physical therapy modalities and kinesio taping treatments in shoulder impingement syndrome	RCT	Y	Y	N	N	
917	Kurebayashi_2020 - Massage and Reiki to reduce stress and improve quality of life: a randomized clinical trial	RCT	Y	Y	N	N	comparison between therapies + no interventio
918	Lahijanian_2020 - Effect of auriculotherapy on constipation severity in patients undergoing hemodialysis	RCT	Y	Y	?	N	seeds

919	Land_2019 - Effect of manual physiotherapy in homogeneous individuals with subacromial shoulder impingement: A randomized controlled trial	RCT	Y	Y	N	N	comparison
920	Landgren_2021 - Ear acupuncture as an adjunct in a treatment protocol for anorexia nervosa: utilization rate and nurses' experience	observational	Y	N	N	N	
**921**	**Lascurain_2021 - Immediate effects of cervical mobilisations on neck muscle activity during active neck movements in patients with non-specific neck pain. A double blind placebo controlled trial**	**RCT**	**Y**	**Y**	**Y**	**Y**	
**922**	**Lascurain_2019 - Association between sympathoexcitatory changes and symptomatic improvement following cervical mobilisations in participants with neck pain. A double blind placebo controlled trial**	**RCT**	**Y**	**Y**	**Y**	**Y**	
923	Lee_2019 - Auricular Acupressure and Positive Group Psychotherapy With Motivational Interviewing for Smoking Cessation	RCT	Y	Y	Y	N	sticker (acu-pellets)
924	Lee_2020 - The Effect of Auricular Acupressure and Positive Group Psychotherapy with Motivational Interviewing for Smoking Cessation in Korean Adults	RCT	Y	Y	Y	N	sticker (acu-pellets)
925	Lee_2021 - Effect of Acupressure on Pre-Exam Anxiety in Nursing Students	RCT	Y	Y	Y	N	full-text requested 05/2 and 28/02, no response
926	Lee_2021 - The effect of auricular acupressure on sleep in older adults with sleep disorders	RCT	Y	Y	N	N	not found

927	Lee_2019 - Effects of auricular acupressure on pain and disability in adults with chronic neck pain	RCT	Y	Y	Y	N	seeds
928	Lee_2021 - Effects of auricular acupressure on blood pressure and stress responses in adults with prehypertension	RCT	Y	Y	N	N	not found
929	Lee_2021 - The effects of auricular acupressure on stress, anxiety, and depression of outpatient nurses in South Korea	RCT	Y	Y	N	N	seeds
930	Lee_2019 - Effects of auricular acupressure on pain and disability in adults with chronic neck pain	RCT	Y	Y	Y	N	same as 927
931	Leite_2021 - Immediate effects of the high-velocity low-amplitude thrust on the heart rate autonomic modulation of judo athletes	Y	Y	N	N	N	
932	Levin_2019 - Effectiveness of P6 stimulation for reduction of nausea and vomiting during caesarean section under combined spinal-epidural anaesthesia: A randomised controlled trial	RCT	N	Y	N	N	
933	Levy_2020 - The effectiveness of foot reflexology in reducing anxiety and duration of labor in primiparas: An open-label randomized controlled trial	RCT	Y	Y	N	N	
934	Liao_2020 - Efficacy of Acupoints Dual-Frequency Low-Level Laser Therapy on Knee Osteoarthritis	RCT	N	Y	N	N	
935	Lim_2019 - Comparative effectiveness of Chuna manual therapy versus conventional usual care for non-acute low back pain: A pilot randomized controlled trial	RCT	Y	Y	N	N	

936	Lizis_2019 - Manual therapy with cryotherapy versus manual therapy with kinesio taping for males with lumbar discopathy: A pilot randomized trial	RCT	Y	Y	N	N	
937	Lo_2019 - The Effectiveness of Spinal Manipulation in Increasing Muscle Strength in Healthy Individuals: A Systematic Review and Meta-Analysis	SR	N	N	N	N	
938	Lohana_2021 - Comparison of mulligan sustained natural apophyseal glides verses mckenzie extension exercises on disability and functional outcomes in patients with acute nonspecific low back pain	RCT	Y	Y	N	N	
939	Lohman_2019 - The immediate effects of cervical spine manipulation on pain and biochemical markers in females with acute non-specific mechanical neck pain: a randomized clinical trial	RCT	Y	Y	Y	N	the same of 701
940	Longhini_2020 - Chest physiotherapy improves lung aeration in hypersecretive critically ill patients: A pilot randomized physiological study	RCT	Y	Y	N	N	
941	Longo_2020 - Physical therapy and precision rehabilitation in shoulder rotator cuff disease	review	N	N	N	N	
942	Lown_2019 - Acupressure to Reduce Treatment-Related Symptoms for Children With Cancer and Recipients of Hematopoietic Stem Cell Transplant: Protocol for a Randomized Controlled Trial	RCT	N	N	N	N	protocol
943	Luo_2019 - Effect of hand-ear acupuncture on chronic low-back pain: a randomized controlled trial	RCT	Y	Y	N	N	

944	Lynge_2019 - Effectiveness of chiropractic manipulation versus sham manipulation on recurrent headaches in children aged 7-14 years, Protocol for a randomized clinical trial	RCT	N	N	N	N	protocol
945	Mafetoni_2019 - Effectiveness of auricular therapy on labor pain: A randomized clinical trial	RCT	Y	Y	Y	N	crystal microspheres
946	Magfirah_2019 - The effectiveness of acupressure therapy and aromatherapy of lemon on the ability of coping and emesis gravidarum in trimester i pregnant women at langsa city community health centre, aceh, indonesia	RCT	Y	Y	N	N	
947	Mahmooda_2020 - Effects of mulligan's mobilization with movements versus myofascial release in addition to usual care on pain and range in knee osteoarthritis	RCT	Y	Y	Y	N	comparison; no placebo
**948**	**Mahmoudikohani_2019 - Effects of acupressure on the childbirth satisfaction and experience of birth: A randomized controlled trial**	**RCT**	**Y**	**Y**	**Y**	**Y**	
949	Manadhar_2021 - Effect of Mulligan’s mobilization with movement and eccentric exercises for lateral epicondylitis in recreational tennis players	RCT	Y	Y	N	N	
950	Manu_2021 - Effect of massage, passive neural mobilization and transcutaneous electrical nerve stimulation on magnetic resonance diffusion tensor imaging (MR-DTI) of the tibial nerve in a patient with type 2 diabetes mellitus induced neuropathy: a case report	case report	Y	N	N	N	
**951**	**Martinez-Hurtardo_2019 - Effects of diaphragmatic myofascial release on gastroesophageal reflux disease: a preliminary randomized controlled trial**	**RCT**	**Y**	**Y**	**Y**	**Y**	

952	Martinez-Jimenez_2020 - Acute effects of myofascial induction technique in plantar fascia complex in patients with myofascial pain syndrome on postural sway and plantar pressures: A quasi-experimental study	quasi-experimental	Y	N	N	N	
**953**	**Martinez-Jimenez_2020 - Pressure and Traction Technique Improves Postural Control More Than Tactile Stimulation in Foot Plantar Fascia: A Randomized Single-Blind Trial**	**RCT**	**Y**	**Y**	**Y**	**Y**	
**954**	**Martinez-Lema_2021 - Immediate effects of a direct myofascial release technique on hip and cervical flexibility in inactive females with hamstring shortening: A randomized controlled trial**	**RCT**	**Y**	**Y**	**Y**	**Y**	
955	Maust_2021 - The effects of soft tissue flossing on hamstring range of motion and lower extremity power	CBA	N	N	N	N	flossing
956	Mawe_2020 - Responses after spinal interventions in a clinical pain practice - A pragmatic observational study	observational	N	Y	N	N	
957	Maxwell_2020 - The effects of spinal manipulative therapy on lower limb neurodynamic test outcomes in adults: a systematic review	SR	N	N	N	N	
958	McDevitt_2021 - Thoracic spine thrust manipulation for individuals with cervicogenic headache: a crossover randomized clinical trial	RCT	Y	Y	N	N	
959	Mehrizi_2020 - The Effect of 3-Liver Pressure Point on Postpartum Severity of Pain: randomized clinical trial	RCT	Y	Y	Y	N	not found
960	Mehyar_2020 - Effect of Grade III Lumbar Mobilization on Back Muscles in Chronic Low Back Pain: A Randomized Controlled Trial	RCT	Y	Y	Y	N	full-text requested 05/2 and 28/02; no response

961	Menek_2019 - The effect of mulligan mobilization on pain and life quality of patients with rotator cuff syndrome: A randomized controlled trial	RCT	Y	Y	N	N	
962	Metzler_2020 - Effect of suboccipital release on pain perception and autonomic reflex responses to ischemic and cold pain	RCT	Y	Y	Y	N	osteopathy
963	Min_2021 - auricular acupressure on the sleep of the elderly using polysomnography, actigraphy and blood test: The effects of Randomized, single-blind, sham control	RCT	Y	Y	Y	N	No Manual Therapy
964	Miralizadeh_2021 - Comparison of the Effect of Foot and Palm Reflexology Massage on Respiratory Distress Syndrome in Premature Infants under Noninvasive Ventilation	RCT	Y	Y	N	N	full-text requested 05/2 and 28/02; no response
965	Mizrah_2021 - Effect of Massage With Lavender Oil on Postoperative Pain Level of Patients Who Underwent Gynecologic Surgery: A Randomized, Placebo-Controlled Study	RCT	Y	Y	N	N	oil vs gel vs no intervention

966	Mobarakabadi_2020 - The effect of P6 acupressure on nausea and vomiting of pregnancy: a randomized, single-blind, placebo-controlled trial	RCT	Y	Y	Y	N	sea-bands
**967**	**Mohammadi_2020 - Effect of pressure on the yinmen point in relief of pain after middle ear surgery: A randomized clinical trial**	**RCT**	**Y**	**Y**	**Y**	**Y**	
968	Mohammadi_2021 - The effect of H7 acupressure on amniocentesis anxiety in pregnant women: A randomized controlled trial	RCT	Y	Y	N	N	
969	Mohammadpourho_2021 - The Effect of Aromatherapy Massage With Lavender and Citrus Aurantium Essential Oil on Quality of Life of Patients on Chronic Hemodialysis: A Parallel Randomized Clinical Trial Study	RCT	Y	Y	N	N	oil vs no oil
970	Mohan_2021 - Gender based variations of mulligan mobilization with movement on chronic nonspecific low back pain	Y	Y	N	N	N	male vs female, same intervention
**971**	**Molassiotis_2020 - The effectiveness of acupressure in the management of depressive symptoms and in improving quality of life in older people living in the community: a randomised sham-controlled trial**	**Y**	**Y**	**Y**	**Y**	**Y**	
972	Momeni_2020 - The Effect of Foot Massage on Pain of the Intensive Care Patients: A Parallel Randomized Single-Blind Controlled Trial	Y	Y	Y	N	N	different massage + no sham
**973**	**Motealleh_2020 - The immediate effect of lumbopelvic manipulation on knee pain, knee position sense, and balance in patients with patellofemoral pain: A randomized controlled trial**	**Y**	**Y**	**Y**	**Y**	**Y**	
974	Movahedi_2020 - The effect of acupressure on quality of life among female nurses with chronic back pain	Y	Y	Y	Y	N	arabic language

975	Mueller_2019 - The effect of Therapeutic Touch on Back Pain in Adults on a Neurological Unit: An Experimental Pilot Study	Y	Y	Y	N	N	no sham
976	Mueller_2019 - Preterm infant massage: Effects of tactile stimulation on wellbeing, growth, and the immune system	N	N	N	N	N	review
977	Mueller_2019 - Tactile stimulation (massage) during pregnancy and labor: Effects on pregnancy complications, growth, and the immune system	N	N	N	N	N	not found
978	Mugii_2019 - Long-term follow-up of finger passive range of motion in Japanese systemic sclerosis patients treated with self-administered stretching	Y	Y	Y	N	N	no sham
979	Munoz_2021 - Long-term follow-up of finger passive range of motion in Japanese systemic sclerosis patients treated with self-administered stretching	Y	Y	Y	N	N	no sham
980	Nadal_2020 - Effects of Manual Therapy on Fatigue, Pain, and Psychological Aspects in Women with Fibromyalgia	Y	Y	Y	N	N	ultrasound sessions
**981**	**Nahayati_2020 - The effect of acupressure on stress and anxiety among patients with multiple sclrosis: A sham-controlled randomized clinical trial**	**Y**	**Y**	**Y**	**Y**	**Y**	
982	Najafi_2019 - The Effect of Manual Acupressure (Point BL32) on Pain Associated with Intramuscular Injections of Magnesium Sulfate	Y	Y	Y	N	N	no sham
**983**	**Najafi_2021 - The Effect of Acupressure on Fasting Blood Glucose and Glycosylated Hemoglobin Levels in Diabetic Patients: A Randomized Controlled Trial**	**Y**	**Y**	**Y**	**Y**	**Y**	

984	Najjari_2019 - The effect of acupressure at PC6 and REN12 on vomiting in patients undergoing inguinal hernia repair: A double-blind randomized clinical trial	Y	Y	Y	N	N	no sham
985	Nakamaru_2019 - Immediate effects of thoracic spine self-mobilization in patients with mechanical neck pain: A randomized controlled trial	Y	Y	Y	N	N	self-mobilization
986	Navaee_2020 - Effect of pre-cesarean foot reflexology massage on anxiety of primiparous women	Y	Y	Y	N	N	no sham
**987**	**Nguyen_2021 - Effects of Mulligan Mobilization with Movement in Subacute Lateral Ankle Sprains: A Pragmatic Randomized Trial**	**Y**	**Y**	**Y**	**Y**	**Y**	
988	Niazi_2020 - The effect of spinal manipulation on the electrophysiological and metabolic properties of the tibialis anterior muscle	Y	Y	Y	N	N	no sham
989	Nikzad_2021 - Comparing the effect of foot reflexology and massage therapy on serum bilirubin levels in neonates with hyperbilirubinemia treated with phototherapy: A randomized clinical trial	Y	Y	Y	N	N	control group received only phototherapy
990	Nim_2021 - Changes in pain sensitivity and spinal stiffness in relation to responder status following spinal manipulative therapy in chronic low Back pain: a secondary explorative analysis of a randomized trial	Y	Y	Y	N	N	no sham
991	Nim_2021 - Spinal manipulation and modulation of pain sensitivity in persistent low back pain: a secondary cluster analysis of a randomized trial	Y	Y	Y	N	N	same as ID990

**992**	**Nogueira_2020 - The Acute Effects of Manual and Instrument-Assisted Cervical Spine Manipulation on Pressure Pain Threshold, Pressure Pain Perception, and Muscle-Related Variables in Asymptomatic Subjects: A Randomized Controlled Trial**	**Y**	**Y**	**Y**	**Y**	**Y**	
993	Nourmohammadi_2019 - The effects of reflexology on fatigue severity of patients with cancer	Y	Y	Y	N	N	Control group received routine care and the prescribed medication by the oncologist
994	Nuhmani_2020 - Effect of lumbar stabilization exercises and thoracic mobilization with strengthening exercises on pain level, thoracic kyphosis, and functional disability in chronic low back pain	Y	Y	Y	N	N	control: only excercises
**995**	**Nunes_2020 - Acute Effects of Hip Mobilization With Movement Technique on Pain and Biomechanics in Females With Patellofemoral Pain: A Randomized, Placebo-Controlled Trial**	**Y**	**Y**	**Y**	**Y**	**Y**	
996	Odynets_2019 - The effectiveness of two individualized physical interventions on the upper limb condition after radical mastectomy	Y	Y	Y	N	N	no sham
997	Ogul_2021 - Effect of acupressure on procedural pain before heel lancing in neonates	Y	Y	Y	N	N	No intervention was applied to newborns in the control group
998	Oshvandi_2021 - The effects of foot massage on hemodialysis patients’ sleep quality and restless leg syndrome: A comparison of lavender and sweet orange essential oil topical application	Y	Y	Y	N	N	The control group received routine care

999	Oviedo_2021 - Auricular acupressure and auricular acupuncture as an adjunct for pain management during first trimester aspiration abortion: A randomized, double-blinded, three-arm trial	\	\	\	\	\	not found
1000	Park_2019 - Is hand massage with the preferred aroma oil better than lavender on stress and sleep for long-term care facility residents?	Y	Y	Y	N	N	difference: the oil
1001	Park_2020 - Comparative effectiveness of chuna manipulative therapy for non-acute lower back pain: A multi-center, pragmatic, randomized controlled trial	Y	Y	Y	N	N	no sham
1002	Pasin Neto_2020 - Visceral Mobilization and Functional Constipation in Stroke Survivors: A Randomized, Controlled, Double-Blind, Clinical Trial	Y	Y	Y	N	N	no sham
1003	Passmore_2019 - Impact of spinal manipulation on lower extremity motor control in lumbar spinal stenosis patients: A small-scale assessor-blind randomized clinical trial	Y	Y	Y	N	N	no sham
1004	Patterson_2020 - The Effect of Shoulder Mobilization on Scapular and Shoulder Muscle Activity During Resisted Shoulder Abduction: A Crossover Study of Asymptomatic Individuals	Y	Y	Y	N	N	no sham
1005	Pawar_2019 - A comparative study to determine the effectiveness of the taping and mulligan’s mobilization with movement techniques on pain in knee osteoarthritis	Y	Y	Y	N	N	no sham
1006	Pehlivan_2019 - Effects of aromatherapy massage on pain, functional state, and quality of life in an elderly individual with knee osteoarthritis	Y	Y	Y	N	N	control group received no aromatherapy or massage

1007	Pei_2020 - Auricular Acupressure for Insomnia in Patients With Maintenance Hemodialysis: A Systematic Review and Meta-Analysis	N	Y	Y	Y	N	SR
1008	Pellicciari_2019 - Mobilization of the contralateral limb in Slump position: Effects on knee extension in healthy adult subjects	Y	Y	Y	Y	Y	
1009	Perze_Domingue_2020 - The effects of a combined physical therapy approach on Morton's Neuroma. An N-of-1 Case Report	N	Y	N	N	N	case report
1010	Perez-LLanes_2020 - Effectiveness of suboccipital muscle inhibition combined with interferential current in patients with chronic tension-type headache: a randomised controlled clinical trial	Y	Y	Y	N	N	no sham
1011	Perkins_2020 - Does acupressure help reduce nausea and vomiting in palliative care patients? A double blind randomised controlled trial	Y	N	N	N	N	wristband
**1012**	**Pessoa_2021 - The addition of thoracic mobilization to aerobic exercise did not alter autonomic function and pain pressure threshold acutely in asymptomatic young people: A randomized controlled trial**	**Y**	**Y**	**Y**	**Y**	**Y**	
**1013**	**Pfuegler_2021 - The immediate effects of passive hip joint mobilization on hip abductor/external rotator muscle strength in patients with anterior knee pain and impaired hip function. A randomized, placebo-controlled crossover trial**	**Y**	**Y**	**Y**	**Y**	**Y**	
**1014**	**Picchiottino_2021 - The effect of a single spinal manipulation on cardiovascular autonomic activity and the relationship to pressure pain threshold: a randomized, cross-over, sham-controlled trial**	**Y**	**Y**	**Y**	**Y**	**Y**	

1015	Pinar_2021 - The effect of therapeutic touch on labour pain, anxiety and childbirth attitude: A randomized controlled trial	Y	Y	Y	N	N	no sham
1016	Pouy_2019 - Evaluating the effectiveness of acupressure on anxiety of mothers in a pediatric surgical waiting area: A randomized clinical trial	Y	Y	Y	Y	N	not found
**1017**	**Pouy_2019 - Effect of acupressure on post tonsillectomy pain in adolescents: A randomized, single-blind, placebo-controlled trial study**	**Y**	**Y**	**Y**	**Y**	**Y**	
1018	Pouy_2019 - The effect of acupressure on post tonsillectomy nausea and vomiting in pediatrics: A randomized, single-blind, sham-controlled study	Y	Y	Y	Y	N	the same sample of 1017
1019	Provencher_2021 - Effects of chiropractic spinal manipulation on laser-evoked pain and brain activity	Y	N	Y	N	N	no manual
1020	Prymaachenko_2021 - Are manual therapy or booster sessions worthwhile in addition to exercise therapy for knee osteoarthritis: Economic evaluation and 2-year follow-up of a randomized controlled trial	Y	Y	Y	N	N	no sham
1021	Queiroz do Santos_2021 - Immediate effects of myofascial release maneuver applied in different lower limb muscle chains on postural sway	Y	Y	Y	Y	N	request 05-02-2022 and 01-03-2022. No response
1022	Rafii_2020 - The effect of aromatherapy massage with lavender and chamomile oil on anxiety and sleep quality of patients with burns	Y	Y	Y	N	N	The control group was only under daily routine care

**1023**	**Rahimi_2020 - Self-acupressure for multiple sclerosis-related depression and fatigue: A feasibility randomized controlled trial**	**Y**	**Y**	**Y**	**Y**	**Y**	
1024	Rahmani_2019 - Effect of acupressure on anxiety and hemodynamic parameters in female patients with acute coronary syndrome hospitalized in cardiac care unit: A randomized placebo controlled clinical trial	Y	Y	Y	Y	N	ARABIC
1025	Rambod_2019 - The effect of foot reflexology on fatigue, pain, and sleep quality in lymphoma patients: A clinical trial	Y	Y	Y	N	N	The control group received usual care.
1026	Rani_2021 - Acupressure combined with pharmacological treatment in patients with osteoarthritis of the knee: a randomized trial	Y	Y	Y	N	N	control group (n = 106) continued pharmacological treatment only
1027	Ranjkesh_2019 - Effects of acupressure at LI4, HE7, SP6 and neima points on labor anxiety in nulliparous women: Randomized clinical trial	Y	Y	Y	N	N	Control group received routine obstetrics cares
1028	Rejeh_2020 - The effect of hand reflexology massage on pain and fatigue in patients after coronary angiography: A randomized controlled clinical trial	Y	Y	Y	N	N	(routine nursing care)
**1029**	**Reynolds_2020 - Effectiveness of Cervical Spine High-Velocity, Low-Amplitude Thrust Added to Behavioral Education, Soft Tissue Mobilization, and Exercise for People With Temporomandibular Disorder With Myalgia: A Randomized Clinical Trial**	**Y**	**Y**	**Y**	**Y**	**Y**	

**1030**	**Rezaien_2019 - The Impact of Soft Tissue Techniques in the Management of Migraine Headache: A Randomized Controlled Trial**	**Y**	**Y**	**Y**	**Y**	**Y**	
1031	Rezaian_2019 - Effects of Dry Needling Technique into Trigger Points of the Sternocleidomastoid Muscle in Migraine Headache: A Randomized Controlled Trial	Y	N	Y	N	N	no manual
**1032**	**Rio-Gonzalez_2020 - Effects of different neck manual lymphatic drainage maneuvers on the nervous, cardiovascular, respiratory and musculoskeletal systems in healthy students**	**Y**	**Y**	**Y**	**Y**	**Y**	
1033	Rivaz_2021 - The effects of aromatherapy massage with lavender essential oil on neuropathic pain and quality of life in diabetic patients: A randomized clinical trial	Y	Y	Y	N	N	different oils
1034	Roberts_2021 - Effect of occipitoatlantal decompression on cerebral blood flow dynamics as evaluated by doppler ultrasonography	Y	Y	Y	Y	N	osteopathy
1035	Rodrigues_2021 - Effects of myofascial release on flexibility and electromyographic activity of the lumbar erector spinae muscles in healthy individuals	quasi experimental	Y	N	N	N	
1036	Rodrigues_2021 - One Session of Spinal Manipulation Improves the Cardiac Autonomic Control in Patients with Musculoskeletal Pain: A Randomized Placebo-Controlled Trial	RCT	Y	Y	N	N	ultrasound as placebo
1037	Rodrigueza_2020 - Treatment of Neck Pain With Myofascial Therapies: A Single Blind Randomized Controlled Trial	RCT	Y	Y	N	N	
1038	Rostron_2021 - The effects of massage therapy on a patient with migraines and cervical spondylosis: A case report	case report	Y	N	N	N	

1039	Sajadi_2019 - The effect of foot reflexology on constipation and quality of life in patients with multiple sclerosis. A randomized controlled trial	RCT	Y	Y	Y	Y	
1040	Sajadi_2020 - Randomized clinical trial comparing of transcranial direct current stimulation (tDCS) and transcutaneous electrical nerve stimulation (TENS) in knee osteoarthritis	RCT	Y	Y	N	N	
**1041**	**Salmani_2021 - The effect of acupressure on fasting blood glucose, glycosylated hemoglobin and stress in patients with type 2 diabetes**	**RCT**	**Y**	**Y**	**Y**	**Y**	
1042	Samarehfekri_2020 - Effect of Foot Reflexology on Pain, Fatigue, and Quality of Sleep after Kidney Transplantation Surgery: A Parallel Randomized Controlled Trial	RCT	Y	Y	N	N	
1043	Satpute_2021 - Effectiveness of Mulligan manual therapy over exercise on headache frequency, intensity and disability for patients with migraine, tension-type headache and cervicogenic headache – a protocol of a pragmatic randomized controlled trial	RCT	Y	Y	Y	N	protocol, no data
1044	Satpute_2019 - The Effect of Spinal Mobilization With Leg Movement in Patients With Lumbar Radiculopathy—A Double-Blind Randomized Controlled Trial	RCT	Y	Y	N	N	
1045	Satpute_2021 - Effectiveness of Mulligan manual therapy over exercise on headache frequency, intensity and disability for patients with migraine, tension-type headache and cervicogenic headache - a protocol of a pragmatic randomized controlled trial					?	same as 1043
1046	Savva_2020 - Cervical traction combined with neural mobilization for patients with cervical radiculopathy: a randomized controlled trial	RCT	Y	Y	Y	N	only one technique gets a sham

1047	Savva_2021 - The effect of lumbar spine manipulation on pain and disability in Achilles tendinopathy. A case report	case report	Y	N	N	N	
1048	Savva_2021 - Cervical traction combined with neural mobilization for patients with cervical radiculopathy: A randomized controlled trial						same as 1046
1049	Sayari_2021 - Effect of foot reflexology on chest pain and anxiety in patients with acute myocardial infarction: A double blind randomized clinical trial	RCT	Y	Y	N	N	control is routine care
1050	Sayari_2021 - Effect of foot reflexology on sleep quality and severity of fatigue in patients with acute myocardial infarction: A double-blind randomized clinical trial	RCT	Y	Y	Y	Y	no full text in english, translation requested on RG. no response
1051	Seyedi_2021 - Efficacy of acupressure on intensity of acute migraine in patients attending an emergency department: A randomized clinical trial	RCT	Y	Y	Y	N	no full text in english, didn't find contact of authors
**1052**	**Silva_2019 - Immediate effects of spinal manipulation on shoulder motion range and pain in individuals with shoulder pain: A randomized trial**	**RCT**	**Y**	**Y**	**Y**	**Y**	
1053	Silva_2019 - Visceral manipulation decreases pain, increases cervical mobility and electromyographic activity of the upper trapezius muscle in non-specific neck pain subjects with functional dyspepsia: Two case reports	case report	Y	N	N	N	
1054	Silva_2019 - Immediate Effects of Spinal Manipulation on Shoulder Motion Range and Pain in Individuals With Shoulder Pain: A Randomized Trial						same as 1052

1055	Simon_2021 - Safety considerations when managing gastro-esophageal reflux disease in infants	review	NA	NA	NA	N	
1056	Simoni_2021 - Effectiveness of standard cervical physiotherapy plus diaphragm manual therapy on pain in patients with chronic neck pain: A randomized controlled trial	RCT	Y	Y	Y	N	unclear if by osteopaths + osteo technique as sham
1057	Stefannson_2019 - Using Pressure Massage for Achilles Tendinopathy: A Single-Blind, Randomized Controlled Trial Comparing a Novel Treatment Versus an Eccentric Exercise Protocol	RCT	Y	Y	N	N	
1058	Stroppa-Marques_2021 -Plantar Myofascial Mobilization: Plantar Area, Functional Mobility, and Balance in Elderly Women: A Randomized Clinical Trial	RCT	Y	Y	Y	N	placebo with oil + not sham, just soft technique
1059	Sun_2018- Auricular Acupressure Improves Habit Reversal Treatment for Nail Biting	pragmatic pilot	Y	Y	Y	N	seeds
1060	Tan_2020 - Spinal Manipulative Therapy Alters Brain Activity in Patients With Chronic Low Back Pain: A Longitudinal Brain fMRI Study	longitudinal study	Y	Y	N	N	
1061	Tanabe_2021 - Immediate effect of mechanical lumbar traction in patients with chronic low back pain: A crossover, repeated measures, randomized controlled trial	RCT	Y	Y	N	N	
**1062**	**Tara_2019 - The Effect of Acupressure on the Severity of Nausea, Vomiting, and Retching in Pregnant Women: A Randomized Controlled Trial**	**RCT**	**Y**	**Y**	**Y**	**Y**	

1063	Thomas_2020 - Effect of spinal manipulative and mobilization therapies in young adults with mild to moderate chronic low back pain: a randomized clinical trial [with consumer summary]	RCT	Y	Y	N	N	
**1064**	**Tomruk_2020 - Immediate Effects of Ankle Joint Mobilization With Movement on Postural Control, Range of Motion, and Muscle Strength in Healthy Individuals: A Randomized, Sham-Controlled Trial**	**RCT**	**Y**	**Y**	**Y**	**Y**	
1065	Topcu_2020 - The impact of reflexology and homeopathy added to conventional asthma treatment on markers of airway inflammation–a randomised study	randomised study	Y	Y	N	N	
1066	Topdemir_2021 - The effect of Acupressure and Reiki application on Patient's pain and comfort level after laparoscopic cholecystectomy: A randomized controlled trial	RCT	Y	Y	N	N	
1067	Topraghlou_2019 - Comparing the effects of acupressure at the hugo point and hyoscine on the duration of labor stages and fetal-neonatal outcomes in nulliparous women: A controlled randomized clinical trial	RCT	Y	Y	N	N	
1068	Torkyian_2020 - Effect of gallbladder 21 (Gb21) acupressure on length of delivery in nulliparous women	RCT	Y	Y	Y	N	different outcome, but same set of patients and intervention as 1069
1069	Torkyian_2021 - The effect of GB21 acupressure on pain intensity in the first stage of labor in primiparous women: A randomized controlled trial	RCT	Y	Y	Y	Y	different outcome, but same set of patients and intervention as 1068

1070	Tornatore_2020 - Effects of combining manual lymphatic drainage and Kinesiotaping on pain, edema, and range of motion in patients with total knee replacement: A randomized clinical trial	RCT	Y	Y	N	N	
1071	Toscano_2019 - Short-term effects of focal muscle vibration on motor recovery after acute stroke: A pilot randomized sham-controlled study	RCT	N	Y	N	N	
1072	Toygar_2020 - Effect of reflexology on anxiety and sleep of informal cancer caregiver: Randomized controlled trial	RCT	Y	Y	Y	N	can't understand if the foot was "wrapped in a towel" the entire time
1073	Tsai_2021 - Yoga versus massage in the treatment of aromatase inhibitor-associated knee joint pain in breast cancer survivors: a randomized controlled trial	RCT	Y	Y	N	N	
1074	Tsai_2021 - Immediate effect of non-invasive auricular acupoint stimulation on the performance and meridian activities of archery athletes: A protocol for randomized controlled trial	RCT (protocol)	Y	Y	Y	N	seeds and tap
1075	Vagedes_2019 - Efficacy of rhythmical massage in comparison to heart rate variability biofeedback in patients with dysmenorrhea—A randomized, controlled trial	RCT	Y	Y	N	N	
**1076**	**Vagharseyyedin_2018 - The impact of self-administered acupressure on sleep quality and fatigue among patients with migraine: A randomized controlled trial**	**RCT**	**Y**	**Y**	**Y**	**Y**	
1077	Valenzuela_2019 - Spinal Manipulative Therapy Effects in Autonomic Regulation and Exercise Performance in Recreational Healthy Athletes: A Randomized Controlled Trial	RCT	Y	Y	Y	N	activator

1078	Villalta_2019 - Active Visceral Manipulation Associated With Conventional Physiotherapy in People With Chronic Low Back Pain and Visceral Dysfunction: A Preliminary, Randomized, Controlled, Double-Blind Clinical Trial	RCT	Y	Y	Y	N	osteopathy
1079	Vining_2020 - Effects of Chiropractic Care on Strength, Balance, and Endurance in Active-Duty U.S. Military Personnel with Low Back Pain: A Randomized Controlled Trial	RCT	Y	Y	N	N	
1080	Visconti_2020 - Comparison of the effectiveness of manual massage, long-wave diathermy, and sham long-wave diathermy for the management of delayed-onset muscle soreness: a randomized controlled trial	RCT	N	Y	N	N	
1081	Vurul_2021 - The Effect of Foot Reflexology Applied before Coronary Angiography and Percutaneous Transluminal Coronary Angioplasty on Anxiety, Stress, and Cortisol Levels of Individuals: A Randomized Controlled Trial	RCT	Y	Y	N	N	
1082	Vurul_2021 - Effect of therapeutic touch on daytime sleepiness, stress and fatigue among students of nursing and midwifery: A randomized sham-controlled trial	RCT	Y	Y	Y	N	no skin conract, hands 5 cm away from the body
1083	Weerasekara_2019 - Effects of mobilisation with movement (MWM) on anatomical and clinical characteristics of chronic ankle instability: A randomised controlled trial protocol	RCT	Y	Y	N	N	
1084	Xu_2021 - The acupoint herbal plaster for the prevention and treatment of postoperative nausea and vomiting after PLIF with general anesthesia: study protocol for a multicenter randomized controlled trial	RCT (protocol)	N	Y	Y	N	plaster, no skin contact

1085	Xu_2021 - Effectiveness of self-myofascial release combined with biofeedback and electrical stimulation for the management of myofascial pelvic pain: A randomized controlled trial	RCT	Y	Y	N	N	
1086	Yang_2021 - Acupressure: An Effective and Feasible Alternative Treatment for Anxiety During the COVID-19 Pandemic	review/commentary	NA	NA	NA	N	
1087	Yangdol_2021 - Effect of visceral manipulation on forward head posture in subjects with chronic non-specific neck pain – a pilot study	pilot study	Y	Y	Y	N	Barral technique
1088	Yildrim_2021 - The effect of acupressure applied to individuals receiving hemodialysis treatment on severity of thirst and quality of life	observational	Y	N	NA	N	
1089	yildrim_2021 - The Effect of Acupressure Applied to Individuals Receiving Hemodialysis Treatment on Severity of Thirst and Quality of Life					N	same as 1088
1090	Yoshimura_2021 - The acute mechanism of the self-massage-induced effects of using a foam roller	case control	N	Y	N	N	
1091	You_2019 - Effects of Auricular Acupressure on Pain Management: A Systematic Review	SR	NA	NA	NA	N	
**1092**	**Young_2019 - Immediate and Short-term Effects of Thoracic Spine Manipulation in Patients With Cervical Radiculopathy: A Randomized Controlled Trial**	**RCT**	**Y**	**Y**	**Y**	**Y**	
1093	Yuan_2021 - Efficacy of the acupressure wrist-ankle strap in mild insomnia patients with anxiety disorders: study protocol for a randomized controlled trial	RCT (protocol)	N	Y	N	N	

1094	Yung_2020 - Non-thrust cervical manipulations reduce short-term pain and decrease systolic blood pressure during intervention in mechanical neck pain: a randomized clinical trial	RCT	Y	Y	N	N	comparison
**1095**	**Zeidabadinejad_2021 - Effect of Foot Reflexology on Sexual Function of Patients under Hemodialysis: A Randomized Parallel Controlled Clinical Trial**	**RCT**	**Y**	**Y**	**Y**	**Y**	
1096	Zhang_2020 - Acupressure therapy and Liu Zi Jue Qigong for pulmonary function and quality of life in patients with severe novel coronavirus pneumonia (COVID-19): A study protocol for a randomized controlled trial	RCT (protocol)	Y	Y	N	N	
1097	Zhang_2021 - Auricular Acupressure for treating early stage of knee osteoarthritis: a randomized, sham-controlled prospective study	randomised prospective study	Y	Y	Y	N	seeds
1098	Zhang_2021 - Acupoint catgut embedding reduces insulin resistance in diabetic patients undergoing open cardiac surgery	RCT	Y	Y	Y	N	seeds
1099	Zhao_2019 - Auricular acupressure as assistant in primary insomnia management: a randomized single-blind controlled clinical trial	RCT	Y	Y	Y	N	seeds
1100	Zhong_2021 - Ear Acupressure for Allergic Rhinitis: A Systematic Review and Meta-Analysis of Randomized Controlled Trials	SR	NA	NA	NA	N	
**1101**	**Chiropractic spinal manipulative therapy for cervicogenic headache: a single-blinded, placebo, randomized controlled trial**	**Y**	**Y**	**Y**	**Y**	**Y**	

Supplementary Table S1: included and excluded studies with reasons for exclusion.

	ACU (77)	MAS (8)	REIKI (2)	REF (20)	TT (3)	MOB (108)	MAN (89)	MIX (4)	TOT (311)
Total studies									
parallel	74	8	2	19	2	79	76	4	264
crossover	3	0	0	1	1	29	13	0	47
5 arms	0	0	0	0	0	1	0	0	1
4 arms	2	1	1	0	0	6	5	0	15
3 arms	33	3	1	5	1	33	21	1	98
2 arms	42	4	0	15	2	68	63	3	197
3 arms of manual intervention	0	0	0	0	0	2	0	0	2
2 arms of manual intervention	5	2	0	1	0	11	11	0	30
1 arm of manual intervention	72	6	2	19	3	95	78	4	279
2 arms of manual control	0	0	0	0	0	3	4	0	7
1 arm of manual control	77	8	2	20	3	105	85	4	304
2 arms of "other"	1	0	1	0	0	0	1	0	3

1 number of "other"	30	2	1	4	1	32	11	1	82
0 arms of "other"	46	6	0	16	2	76	77	3	226

Supplementary Table 2(S2): methodological characteristics of the included studies.

	**ACU**				**MAS**				**REIKI**				**REF**				**TT**				**MOB**				**MAN**				**MIX**			
	L	M	H	U	L	M	H	U	L	M	H	U	L	M	H	U	L	M	H	U	L	M	H	U	L	M	H	U	L	M	H	U
Sequence generation	48	2	1	26	5	1	1	1	2	0	0	0	16	0	0	4	0	1	0	2	69	4	4	31	62	5	0	22	2	0	0	2
Allocation concealment	31	0	1	45	5	0	0	3	0	0	0	2	9	0	0	11	1	0	0	2	45	7	4	52	41	4	2	42	4	0	0	0
Blinding to personnel	26	6	7	38	4	1	1	2	1	1	0	0	8	3	3	6	1	0	1	1	63	17	8	20	46	10	8	25	3	0	0	1
Blinding to outcome analysis	18	2	2	55	1	0	1	6	0	0	0	2	8	0	0	12	0	0	0	3	24	1	5	78	23	0	6	60	0	0	1	3
Incomplete outcome data	77	0	0	0	8	0	0	0	2	0	0	0	20	0	0	0	3	0	0	0	106	1	1	0	88	0	1	0	4	0	0	0
Selective outcome reporting	77	0	0	0	8	0	0	0	2	0	0	0	20	0	0	0	3	0	0	0	107	0	1	0	89	0	0	0	4	0	0	0
Contamination	18	0	0	59	7	0	0	1	0	0	0	2	6	0	0	14	0	0	0	3	34	0	0	74	23	0	1	65	0	0	0	4
Valid tools	70	1	0	6	8	0	0	0	2	0	0	0	20	0	0	0	2	0	0	1	101	0	0	7	81	2	0	6	4	0	0	0

Supplementary Table 3(S3): Risk of Bias of the included studies. L = low; M = medium; H = high; U = unclear
